# Cyclic Isothiocyanate Goitrin Impairs *Lotus japonicus* Nodulation, Affects the Proteomes of Nodules and Free *Mesorhizobium loti*, and Induces the Formation of Caffeic Acid Derivatives in Bacterial Cultures

**DOI:** 10.3390/plants13202897

**Published:** 2024-10-16

**Authors:** Seungwoo Jeong, Vadim Schütz, Fatih Demir, Matthias Preusche, Pitter Huesgen, Laurent Bigler, Filip Kovacic, Katharina Gutbrod, Peter Dörmann, Margot Schulz

**Affiliations:** 1 IMBIO Institute of Molecular Biotechnology, University of Bonn, 53115 Bonn, Germany; s6sejeon@uni-bonn.de (S.J.); v_schuetz@snu.ac.kr (V.S.); m.preusche@hs-osnabrueck.de (M.P.); k.gutbrod@uni-bonn.de (K.G.); doermann@uni-bonn.de (P.D.); 2 Department of Biomedicine, Aarhus University, 8000 Aarhus, Denmark; fatih.demir@biomed.au.dk; 3 Faculty of Agricultural Sciences and Landscape Architecture, University of Applied Sciences Osnabrueck, 49090 Osnabrueck, Germany; 4 Faculty of Biology, University of Freiburg, 79104 Freiburg, Germany; pitter.huesgen@biologie.uni-freiburg.de; 5 Department of Chemistry, University of Zurich, CH-8057 Zurich, Switzerland; laurent.bigler@chem.uzh.ch; 6 Institute of Molecular Enzyme Technology, Heinrich-Heine-University of Düsseldorf, Forschungszentrum Jülich, 52428 Jülich, Germany; fkovacic@mgh.harvard.edu

**Keywords:** *Brassica* glucosinolate, caftaric acid, chicoric acid, cyclic isothiocyanate goitrin, *Mesorhizobium loti*, *Lotus japonicus*, nodulation, proteomics

## Abstract

The continuous release of glucosinolates into the soil by Brassicaceae root exudation is a prerequisite to maintaining toxic levels of breakdown products such as isothiocyanates (ITCs). ITCs influence plant and microbial diversity in ecosystems, while fungi and Rhizobiaceae are particularly injured. Studies explaining the molecular mechanisms of the negative effects are presently limited. Therefore, we investigated the early effects of cyclic ITC goitrin on proteomes of the host and symbiotic *Mesorhizobium loti* in the nodules of *Lotus japonicus* and of free-living bacteria. In the nodules, many host proteins had a higher abundance, among them, peroxidases and pathogenesis-related PR-10 proteins functioning in the abscisic-acid-activated signaling pathway. In the microsymbiont, transporter proteins as a prominent group are enhanced; some proteins involved in N-fixation decreased. The proteomes give a report about the loss of immunity suppression resulting in the termination of symbiosis, which initiates nodule senescence. Free-living *M. loti* are severely damaged, indicated, i.a., by a decrease in transporter proteins, the assumed candidates for goitrin protein complex formation, and high proteolysis. The production of chicoric acid by the accompanying bacteria is inhibitory for *M. loti* but connected to goitrin elimination, as confirmed by mass spectrometric (MS) analysis. In summary, the nodulation process is severely affected by goitrin, causing nodule dysfunction and failed nodule development. N deficiency conditions leads to yellowish leaves and leaf abscission.

## 1. Introduction

Glucosinolates are the characteristic, glucosylated secondary metabolites of the Brassicaceae and a few other dicotyledonous families. The compounds have a large structural diversity across the different species, but quality and quantity can vary between organs and depend on plant age, nutrient availability, and further environmental conditions [1,2,3,4,5,6,7,8]. For instance, other plant species in the neighborhood can stimulate the biosynthesis and root exudation of glucosinolates into the soil. The removal of the sugar moiety by myrosinase and myrosinase-like enzymes leads to the formation of isothiocyanates, thiocyanates, and nitriles. These toxic degradation products are often converted into further downstream products with insufficiently investigated bioactive properties [9].

Many isothiocyanates are volatile compounds detectable in the air and in the soil after *Brassica* cultivation [10]. Although isothiocyanates have a short lifetime, spanning from hours to days, they exert a strong allelopathy against many plants and microorganisms in natural habitats. The continuous release into the soil, for instance, by living plants via root exudation, is a prerequisite to maintaining toxic levels of the bioactive, short-lived breakdown products. For example, *Alliaria petiolata*, a Brassicaceae native to Eurasia, is an aggressive invader in the USA. The allelopathic success of *A. petiolata*, based on the volatile allyl isothiocyanate, is derived from sinigrin that destructively interferes with soil fungi living in a mutualistic lifestyle with native plants [2,11,12]. Additionally, Lankau [12] reported significant but temporary shifts in the soil bacterial species composition when exposed to *A. petiolata*. The suppression of native species is thought to be maintained until co-evolutionary processes create a new adaptive balance between the species [13].

The decline in the total microbial biomass encompassing mycorrhizal and other fungi, Gram-positive and -negative bacteria, concomitant with changes in microbial community structures, have been often described in agricultural systems and were also recognized by in vitro soil experiments [8,14,15,16,17]. Aside from the fungal communities, species of bacteria belonging to the Rhizobiaceae are particularly affected by isothiocyanates. The effects are only short-lasting when the compounds or glucosinolate-rich plant material are not continuously added. Specialized soil microorganisms may degrade the toxins, paving the way for the recovery of more sensitive bacteria and fungi.

Recently, Portales-Reyes et al. [18] investigated the effects of synthetic allyl isothiocyanate (0.017 μg g^−1^ soil) and benzyl isothiocyanate (1.19 μg g^−1^ soil) on the legume *Amphicarpaea bracteata* inoculated with *Bradyrhizobium* sp. The treatment caused a reduced number of nodules. Portales-Reyes et al. [18] assumed disrupted interactions between *Amphicarpaea bracteata* and its mutualistic rhizobia. However, under greenhouse conditions, nodulation was not affected, although the application of the isothiocyanates were repeated once a week over a period of two months. Regarding the results of Portales-Reyes et al. [18], it can be speculated that a rapid loss of the applied isothiocyanates, due to their volatile nature, could be a reason for the failed effects under greenhouse conditions. For instance, the half-life of 67 nmol benzyl-ITC/g soil was determined to be only 0.2 days [19]. Under non-sterile conditions, the microbial degradation of glucosinolate breakdown products may be even faster [20].

In contrast, the cyclic isothiocyanate goitrin ((R)-5-vinyl oxazolidine-2-thione, epigoitrin) from glucosinolate progoitrin degradation, is more stable. Epi/Progoitrin is widespread in the *Brassica* genus. In cold-pressed rapeseed cake, Miklavčič Višnjevec et al. [21] determined progoitrin and goitrin in remarkable amounts (1014 ± 56 mg/kg, 22 ± 1.5 mg/kg). In turnip (*Brassica rapa* subsp. *rapa*), 1.32–2.55 μmol goitrin/g dw was determined [22]. Progoitrin is a major glucosinolate of broccoli (*Brassica oleracea* convar. *botrytis* var. *cymosa*) and brussels sprouts (*Brassica oleracea* var. *gemmifera*). It is also present in Chinese kale (*Brassica oleracea* var. *alboglabra*), Ethiopian kale (*Brassica carinata*), kale (*Brassica oleracea* var. *acephala*) and rape kale (*Brassica napus*) [23], or in *Eruca vesicaria* (rucola) [24]. Goitrin is degraded slower by soil bacteria than other isothiocyanates, as was found in the strains of *Bacillus megaterium*, *Aminobacter aminovorans, Paenibacillus polymyxa*, or *Bacillus cereus* [16]. The pathway of goitrin degradation is largely unknown.

**Figure 1:**
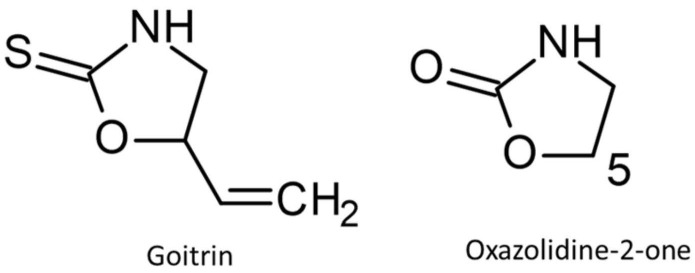
Structures of (R)-5-vinyl oxazolidine-2-thione (goitrin) and oxazolidine-2-one.

In *Barbarea vulgaris* leaf extracts, Agerbirk et al. [25] measured oxazolidine–thionase activity. Thus, goitrin, (R)-5-vinyl oxazolidine-2-thione), can be enzymatically converted into the corresponding oxazolidine-2-one, a class of compounds known for their inhibitory effect on bacterial translation [25,26] (Figure 1). According to Agerbirk et al. [25], the conversion of goitrin to the corresponding oxazolidine-2-one also occurs spontaneously in the presence of 1% H_2_O_2._

The main action of all oxazolidinone-based antibiotic drugs, such as linezolid, is binding to the A-site pocket of the 50S subunit at the peptidyl transferase center (PTC), which results in the inhibition of the initiation complex and of the translocation of peptidyl-tRNA from A site to P site [27]. The carbon atom C-5 of the oxazolidin-2-one ring is crucial for antibacterial activity [28]. A linezolid variant possessing the thio-analog (oxazolidine-2-thione) as a core structure was unable to inhibit protein biosynthesis. The influence of a vinyl group at C-5 on interactions with the 23S rRNA—which is necessary to inhibit translation—is unknown. A direct interaction of goitrin with suitable peptides is likely. For instance, glutathione binding is possible via the N atom of the heterocycle [29]. In feces extracts of the kale- and cabbage-fed herbivore *Helicoverpa armigera*, a cyclic product of goitrin-Cys, probably derived from goitrin-CysGly, was indicated [30]. The fate of the cyclic product, existing in very low amounts in the samples, is not known.

The growth of selected bacterial species that survive rapeseed extract treatments was hardly affected by goitrin over a period of 5 days [16]. However, this study did not include isothiocyanate sensitive *Rhizobium*/*Mesorhizobium* species. If sensible strains of mutualistic *Rhizobia* are affected, consequences of the nodulation and N-supply of the host legume can be expected. To obtain insights in the possible interferences of goitrin with the proteins of *Mesorhizobium loti*, we started a proteomic study with goitrin-treated *M. loti* cultures and with nodules from *Lotus japonicus* plants treated with goitrin after the first inoculation. In silico molecular docking analysis disclosed possible candidates for goitrin complex formation.

## 2. Results

### 2.1. Effects on Nodulation and Plant Phenotype

**Figure 2:**
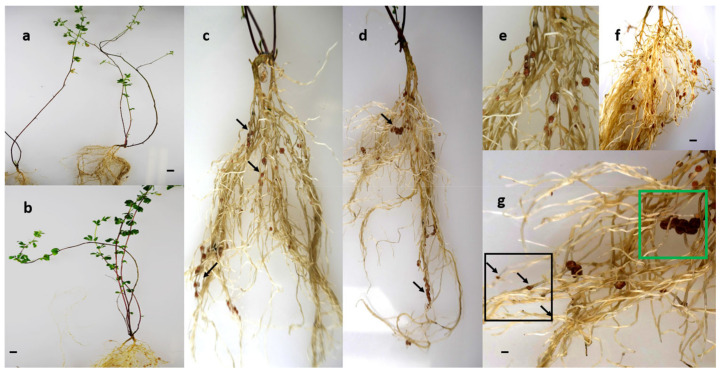
(**a**) Goitrin-treated *L. japonicus* plant. (**b**) Control plant. (**c**,**d**) Distribution of large nodule clusters (arrows, green-lined section). (**e**) Enlarged part of (**c**). (**f**) Distribution of nodules at the root system of a control plant. (**g**) Arrows in the black-lined section point to very small nodules at the roots of a goitrin-treated plant. Scale bars: (**a**,**b**) 1 cm; (**f**) (control) 2 mm; (**g**) (goitrin-treated) 1 mm.

In comparison to the controls, goitrin treatment of inoculated *Lotus japonicus* affected nodule growth and distribution. The nodules from goitrin-treated plants considerably varied in size. Large, dark-colored nodules were clustered only at a few roots, while the very small, even pinhead like nodules, were more evenly spread (Figure 2). Goitrin-treated plants had predominately yellowish leaves at the young shoot part while the older parts lost the leaves.

The phenotype of the treated plants suggested a negative influence of goitrin on the plant and on nodulation. From these results, we assumed that the already free-living bacteria were severely damaged by goitrin.

The large nodules are attributed to the first inoculation prior to the treatment. Subsequently, goitrin applications, which started with a second inoculation, exacerbated disturbed nodulation and nodule development. When goitrin enters the cells, proteins are among the first contacted molecules. These proteins, directly altered by goitrin or indirectly by the resulting oxidative stress, may be responsible for initiating signal transduction pathways that subsequently alter the proteome. Our intention was to first dissect features of the large nodule proteomes to elucidate whether these nodules have a changed protein abundancy which could have consequences for metabolic functions, particularly for N-fixation. Secondly, the proteomes of the free-living *M. loti* were investigated for goitrin-induced alterations that may explain the disturbed nodulation. The total dataset and tables presenting the distinct functional classes of proteins, protein abundance, and the significance of changes as well as their estimation as putative goitrin docking candidates, are parts of the Supplementary Materials. Multifunctional proteins can be listed in more than one table and figure. Proteins belonging to important and affected functional classes are presented with their UniProt protein IDs and the therein annotated function(s), if known [31].

### 2.2. Goitrin Terminates the Symbiotic Lifestyle

#### 2.2.1. Large Nodule Proteome

**Figure 3:**
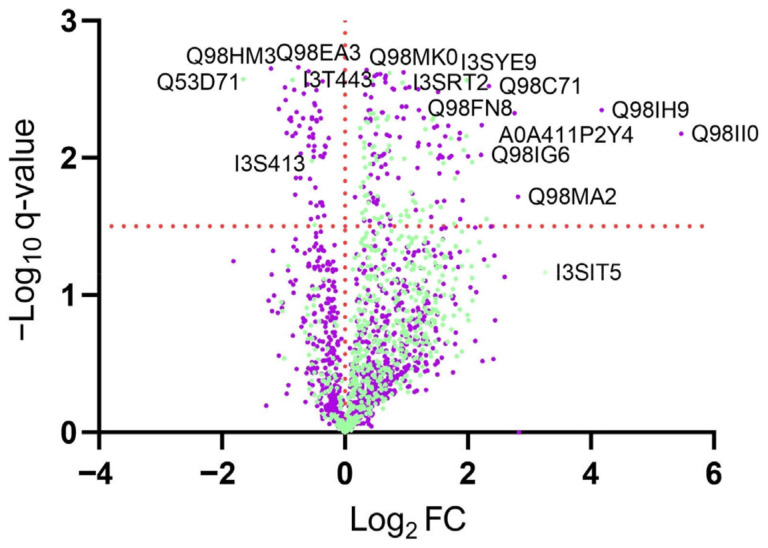
The volcano plot of the entire protein dataset illustrates a higher increase in plant proteins (green dots) and a preferential decrease in defined microsymbiont proteins (purple dots) in the large nodules, while microsymbiont chaperones Q98IH9 and Q98110 are the proteins with the highest increase in abundance. Extracellular space protein Q98HM3 containing a DUF domain and aconitate hydratase Q98EA3 is downregulated with high significance. The two host proteins Q53D71, superoxide dismutase and profilin I3T443, are reduced with highest significance. Alpha-L-fucosidase I3S413 is the third significantly downregulated host protein. I3SYE9, an uncharacterized host protein, and I3SRT2, one of the seven upregulated PR-10 group Bet v I/Major latex proteins, belong to the proteins with the highest increase in abundance.

In the large nodules, a total of 1744 protein groups were identified, composed of 629 plant and 1115 *M. loti* proteins (Figure 3). Comparisons of the group data (control vs. goitrin-treated), obtained from five repetitions, revealed differentially abundant proteins (log_2_ fold-change > 0.3 or <−0.3). Changes where −log_10_ (*t*-test q value) ≥ 1.5 were considered significant. In the plant protein group, 384 proteins were upregulated, 96 of them with significance, and 38 proteins were downregulated, only three of them with significance, and 205 were unchanged or almost unchanged without significance (specified range: 0.3 to −0.3 log_2_ FC). In the microsymbiont group, 509 proteins were upregulated, 123 of them with significance, 195 proteins were downregulated, 72 with significance, and 216 proteins did not, or almost did not, change in abundance. The affected proteins of the large nodule proteome are presented in Tables S1A–S15A, S17A (*M. loti*), and Tables S1B–S17B (host).

#### 2.2.2. Transcription

In the host cell, proteins involved in transcription were hardly changed in abundance, but changes in variant histones H3, H2A, and H2B were insignificant. Variant H3 showed the highest increase, displaying gene activations upon goitrin treatment. Proteins from hormone-related signal transduction systems, which have functions in transcription, are separately mentioned below.

In contrast, symbiont proteins with functions in transcription were more affected. The Mll3846 protein for the regulation of DNA-templated transcription, and Q98M59, a probable transcription regulator, were significantly more abundant. Thirteen others showed a tendency to accumulate, among them, two members of the phosphorelay signal transduction system (histidin kinase Q988T3, Q98FM8 sensory transduction regulatory protein). Q987Y5, a member belonging to the less complex two-component system, is slightly downregulated. Two-component signal transduction systems and the phosphorelays are important for stress adaption but have additional functions [32]. A higher number of affected proteins involved in transcription and signal transduction discloses a stronger response of the symbiont on the transcription level.

#### 2.2.3. Ribosomal Proteins and Translation

The implementation of stress coping programs proceeds on the protein level. The proteome also reports on failed stress adaption. As a response to goitrin, plant and symbiont ribosomal proteins show differences in comparison to the control nodules, while bacterial ribosomes and translation processes are heavily affected. In the bacteroid, two ribosomal proteins were depleted (elongation factor Tu, uS8), seven without significance. Fourteen others marginally or moderately increased in abundance, among them ribosome hibernation promoting factor Q98GS6, but all upregulations were insignificant. The downregulations point to starting dysfunctions in protein synthesis, although the process of translation was not yet hindered, and still allows the upregulation of defined proteins.

All plant ribosomal proteins increased, and the highest FCs were found with uL11, S17, and L3. One protein, eukaryotic translation initiation factor 5A (I3SPM6), increased with significance but with low FC. In plants, translation initiation factor 5A is essential for the first peptide bond and also improves stress tolerance by increasing protein synthesis [33]. The increase in many plant proteins, while only two were downregulated with higher significance (superoxide dismutase and actin polymerization affecting profilin), points to a shift in metabolic processes and alterations in the plant–symbiont relationship.

#### 2.2.4. Chaperones

Chaperones support correct protein folding and protect proteins from irreversible aggregation during synthesis and stress. Chaperone enhancement is a known stress reaction in bacteria [34,35]. Co-chaperonin GroES 2 and chaperonin GroEL 2, absent in the free-living bacteria, are the uppermost increased proteins in the large nodules. Also, chaperone proteins HtpG and GrpE, which have functions in stress protection, were significantly enhanced. Another six additional proteins with chaperone/chaperonine functions were upregulated without significance. Three chaperones were unchanged and chaperonin GroEL 3, a candidate for goitrin docking (see below), was the only one which tended to decrease. Peptidyl-prolyl-cis-trans isomerase Q98M53 was slightly but significantly increased, and peptidyl-prolyl-cis-trans isomerase Q98LE8 insignificantly increased. These proteins have chaperone functions. The significant increase in phasin and phasin-related proteins Q98MA2, Q988T6, and Q988Y4 is notable. The increase is in accordance with the significantly upregulated assumed PHA synthesis regulator protein, Q98FB9. Among other functions, phasins promote bacterial growth, enhance fitness and resilience, and probably possess tolerance to toxic compounds. Phasins, PHA (polyhydroxyalkanoate) granule surface associated proteins, have been shown to perform chaperone-like activities for counteracting stress reactions [36,37,38].

Six plant chaperones and proteins with chaperone functions were slightly upregulated, but none with significance. I3SD49, a calnexin homolog at the ER membrane with chaperone and signaling function, was significantly increased in abundance. It undergoes distinct post-translational modifications which enable the coordination of ER functions with proteins localized at other cellular compartments [39].

#### 2.2.5. Transporters and Outer Membrane Proteins

Bacterial ABC importers usually contain up to two transmembrane permease proteins, up to two nucleotide-binding proteins, and a specific periplasmic solute-binding protein. Predominantly, the solute-binding proteins of ABC transporters were affected.

In the microsymbiont, a highly affected functional class are transporters and membrane proteins. None of the identified transporter proteins were significantly reduced and eighteen increased in abundancy. Six of them function in amino acid transport (Q987A7, Q98MK0, Q98H18, Q98CD0, Q98D26, and Q98EE6) and one in dipeptide transport (Q98L42). Q98JY5, a binding protein component of the sugar ABC transporter, is significantly upregulated. Q98IL8, a protein related to the type VI secretion system-associated lipoprotein, was not significantly upregulated. The type VI secretion system is important for the secretion of toxins during pathogenesis and for the suppression of bacterial competitors. Genes encoding the Type IV secretion system are part of the *M. loti* strain-specific symbiosis island [40]. Q98GF4 and Q98FL2 for phosphate/phosphite/phosphonate transport significantly increased, as did periplasmic binding protein Q98M72, and Q98DQ2 for manganese transport as well as the iron transporter Q983Q2. Sulfate transporter Q98H21 increased in abundance but without significance. The outer membrane protein NodT candidate Q98LA8, which exhibits efflux transmembrane transporter activity, and Q98MN8, a lipoprotein encoded within an ATP-binding cassette, slightly increased with significance. Q987M5/Q988Z1, a putrescine-binding periplasmic protein for putrescine transport, spermidine/putrescine ABC transporter Q98JX0, and the spermidine/putrescine binding protein of ABC transporter Q98JB0 had a significantly higher abundance. Polyamines are thought to support nodulation and have roles in bacterial stress response. Recently, Solmi et al. [41] demonstrated putrescine for reducing the susceptibility to H_2_O_2_, which was correlated with a higher catalase gene expression in *Pseudomonas syringae*. However, in *M. loti*, catalases were not increased in abundance, and in the host, catalase I3T276 was negligibly increased without significance.

Stress-induced LPS-assembly protein LptD (Q984S4), a protein important for membrane integrity, was significantly increased. Outer membrane protein assembly factor BamA Q98MC3 was significantly upregulated (see below). Efflux transmembrane transporter Q98BL6 was slightly enhanced, as was Q986P8, a probable sugar transporter. Q98DB8, a secreted sugar-binding protein involved in sugar transport, was slightly but significantly higher in abundance, similar to outer membrane acid phosphatase Q98HU7. Q98GK5, an RND (resistance-nodulation-division) efflux membrane fusion protein for the active efflux of antibiotics, was another upregulated *M. loti* transporter. In contrast to *M. loti*, plant transporters and outer membrane proteins are almost not affected. Only ADP/ATP translocase I3T6D5 was slightly and insignificantly enhanced.

#### 2.2.6. Cell Surface Polysaccharides and Carbohydrate Metabolism

Cell surface polysaccharides have diverse functions in plant–microbe interactions and in the establishment of symbiosis, for instance, in infection thread formation [42]. The regulation of the biosynthesis of the exopolysaccharide (EPS) group is complex and depends on many factors, for instance, nutrient availability. EPS also have reactive oxygen species (ROS) scavenging properties [43].

In microsymbiont enzymes involved in the synthesis/transport of exopolysaccharides, lipopolysaccharide (LPS) and peptidoglycan are enhanced. Also, endo-1,3-1,4-beta-glycanase ExoK (Q98C78) and plant pectin acetylesterase, acting in cell wall organization, are upregulated, as well as (Uridine diphosphate)- UDP-sulfoquinovose:(Diacylglycerine) DAG sulfoquinovosyltransferase. However, Q98KB6, a D-alanine-D-alanine ligase involved in peptidoglycan-biosynthesis by forming UDP-N-acetylmuramoyl pentapeptide, is downregulated. The altered abundance of these enzymes upon goitrin treatment point to cell wall reorganizations. Since pectin acetylesterase is significantly upregulated, the plant cell wall is also probably affected. Noteworthy to mention, Chungopast et al. report on upregulated genes involved in cell wall biosynthesis during *L. japonicus* nodule senescence [44].

Only a few proteins with roles in glycolysis and glyconeogenesis showed an altered abundance. In the microsymbiont, fructose-bisphosphate aldolase Q986N8 was significantly upregulated and fructose-bisphosphate aldolase Q98FJ0 was significantly downregulated. Dihydrolipoyl dehydrogenase Q98MY4 was slightly upregulated and dihydrolipoyl dehydrogenase Q98ED5 was significantly downregulated. In the host cells, enzymes involved in glycolytic processes were upregulated, except for phosphoglycerate kinase I3SY55, which was significantly downregulated. Since PGK is a key enzyme in glycolytic processes, its loss in abundance could reduce glycolysis and glyconeogenesis although other enzymes of the pathways increased. *M. loti* glucuronate isomerase Q98CW1, necessary for inositol catabolism, was significantly upregulated. Inositol catabolism was suggested to be related to bacterial root colonization [45].

#### 2.2.7. Tricarboxylic Acid Cycle and Energy

In the microsymbiont, succinate Co-A ligase and two citrate synthases are upregulated, while aconitate hydrolase Q98EA3 is significantly downregulated. Aconitase catalyzes the conversion of citrate to isocitrate via aconitate. Aconitase is a moonlighting protein that turns to an iron-responsive-element binding protein (IRE-BP) after losing its 4Fe–4S FeS cluster under low iron conditions. The downregulation of aconitase abundancy and upregulation of citrate synthase enzymes may increase citrate concentration. Citrate activates the glyoxylate cycle, which is important for stress defense (see below, free-living *M. loti*). In the host cells, at least four enzymes of the TCA cycle are upregulated (isocitrate dehydrogenase I3T5L9 and I3SPT9 for the oxidative decarboxylation of isocitrate to 2-oxoglutarate; dihydrolipoyllysine-succinyl transferase for lysine degradation; malate dehydrogenase; and succinyl-CoA ligase).

In plant cells, members of the NADH:ubiquinone oxidoreductase (complex I) and other proteins of the respiratory chain are all upregulated, and I3SPJ0 is significantly upregulated. NADH:ubiquinone oxidoreductases couple the electron transfer between NADH and quinone to proton translocation [46]. Also, subunits of the ATP synthase are upregulated. Thus, goitrin treatment elicits energy consuming metabolic alterations in the host cells. In the symbiont, NADH dehydrogenase [ubiquinone] 1 alpha subcomplex subunit Q98NC7, a member of bacterial complex I, is significantly upregulated. However, all other proteins associated with the respiratory chain are significantly downregulated, such as cytochrome oxidases and NADH dehydrogenase Q98CD7. Also, subunits of the ATP synthase decreased in abundance.

#### 2.2.8. Nitrogen Fixation, Amino Acids, and Polyamines

The proteome analysis disclosed dysfunctions in nitrogen fixation, while imbalanced protein abundancy is thought to be a key factor. Numerous proteins of the fixation complex are downregulated, prominently, nitrogenase molybdenum-iron protein alpha and beta chain. Nitrogenase iron protein Q98AP7 was less but significantly downregulated. Other not significantly downregulated proteins are, for instance, two iron-sulfur ferredoxin proteins, Nif-specific regulatory protein, or protein FixC. Another group of proteins involved in N-fixation were less affected or not affected, while some were slightly upregulated, such as nitrogenase molybdenum-iron protein NifX or nitrogenase-stabilizing/protective protein NifW. Leghemoglobins were not affected, except for insignificantly downregulated atypical leghemoglobin 2-1, which protects nitrogenase from inhibition by O_2_ and other inhibitory molecules.

Protein P-II Q98EH0, a member of the two-component regulatory system NtrB/NtrC, which controls the expression of nitrogen-regulated (ntr) genes in response to nitrogen limitation, was significantly upregulated. PII proteins are key regulators of cellular metabolism, including nitrogen-related processes [47]. Therefore, the upregulation of Q98EH0 reports malfunction in the nodule nitrogen metabolism and the necessity to start counteractions.

Several proteins involved in amino acid biosynthesis are differentially changed in abundance. Asparagine synthase Q98AR2, glutamate synthase Q98H51, Q98KK7 for branched amino acid biosynthesis, Q98E57 for leucine biosynthesis, and Q98E71 for phenylalanine and tyrosine biosynthesis are upregulated, while aspartate aminotransferase Q98KB6, serine ammonia lyase Q98D64, and Q98NN0 possessing glutaminase activity are downregulated. Notably, five polyamine binding proteins are upregulated, three of them with significance, while Q98AL8, a protein with functions in amine and polyamine biosynthesis, is significantly downregulated. Legume root nodules are known to accumulate high levels of polyamines, mainly putrescine, during nodule development [48]. In contrast to former assumptions, a function in nitrogen fixation was recently suggested [49]. A decline in free polyamine content is argued as a stress and senescence marker [50].

In the host, two aspartate aminotransferases, cyanoalanine nitrilase, 2-isopropylmalate synthase for L-leucine biosynthesis and chorismate mutase, involved in the biosynthesis of phenylalanine and tyrosine, are slightly enhanced without significance.

#### 2.2.9. Lipid Metabolism and Lipid Transport Proteins

Several proteins with functions in lipid metabolism and transport were affected in the host cells but none were affected significantly. A protein acting in the transfer of phosphor and galactolipids (I3SY44), a protein possessing a MD-2-related lipid recognition domain (I3SA64), phospholipase D (I3S9D4, 3-hydroxyacyl-[acyl-carrier-protein] dehydratase I3T5Z3, plastid lipid-associated protein I3S0B9, and annexin (I3SXY1) increased in abundance. Plant annexins regulate Ca^2+^ channels, bind calcium-dependently to phospholipids and other membrane lipids, and possess peroxidase and ATPase/GTPase activities. They are also involved in stress adaption [51].

In the microsymbiont, nine lipid-related proteins were changed upon goitrin treatment. Three proteins with transfer/transport functions were upregulated, one of them (Q98G53) significantly. Another two proteins involved in lipid and fatty acid biosynthesis were upregulated, the acyl-carrier protein AcpP (Q988Y6) significantly. Two proteins acting in lipid catabolism were enhanced in abundance (Q9898FS8 significantly). Protein Q983L8 acting in lipopolysaccharide biosynthesis and Q98ET6 (fatty acid synthase cyclopropane-fatty-acyl-phospholipid synthase) were considerably downregulated but without significance.

#### 2.2.10. Hormones and Signal Transduction

The down regulation of two cytochrome P450 enzymes involved in the bacterial GA-synthetic pathway was significant. In GA biosynthesis, Q989L9, a candidate for goitrin docking, catalyzes the oxidation of ent-kaurene into ent-kaurenoic acid, and P450 (Q989M2) converts GA12 into GA9, which is believed to be secreted by the bacteroids for conversion to GA4. GA4 formation is performed in cooperation with the plant [52]. While inactive in plant growth bioassays, GA9 can prevent new nodulation [52]. However, according to Nett et al. [53], the influence of GA9 on nodulation is questionable and GA9 is thought to serve only as the precursor of plant-produced GAs. GA4 was found to increase nodule size. A downregulation of the two P450s would restrict GA9 production. Moreover, gibberellins inhibit nodule senescence. A preliminary experiment revealed that nodules from goitrin-treated plants contained a 3.5-fold higher content of GA9 and a 1.5-fold higher content of GA4, compared to the control (Figure S2). The higher GA4 content agrees with a function in promoting the size of the nodules, which were developed prior to goitrin treatment. The finding needs further investigation, particularly as gibberellins are also antagonist actors of the ethylene-JA and ABA pathways [54]. Gibberellins seem to play roles in regulating brassinosteroids biosynthesis, a class of phytohormones with many functions in plant physiology [55]. In this context, it is interesting that plant delta(24)-sterol reductase I3SM85, necessary for brassinolide biosynthesis, is one of the few downregulated plant proteins.

The plant PR-10 group/Bet v 1 family was highly affected with seven upregulated members (I3SSP7, I3SPM5, I3SL56, I3T2N7, I3SGW8, I3SRT2, and I3SQK0). The PR-10 proteins have functions in the abscisic-acid-activated signaling pathway and show promiscuous ligand binding, including caffeic acid, flavonoids, sterols, and cytokinins [56]. H_2_O_2_, most likely produced during the response to goitrin treatment, also functions as a signal molecule. As H_2_O_2_ interplays with abscisic acid [57], the increase in ABA-responsive proteins is not surprising. Recently, Wen et al. described that increased ABA levels in apples are important in N deficiency-induced leaf senescence [58]. The significantly enhanced 14-3-3 proteins I3T321 and I3S2P9 function in signal transduction via the recognition of phosphorylated target proteins. The 14-3-3 proteins activate Em gene expression by interacting with the basic leu-cine zipper factor EmBP1 and with VIVIPAROUS1 [59].

#### 2.2.11. Glutathione

Four *M. loti* glutathione-S-transferases increased in abundance, one of them (Q983T8) with significance. Glutamate-cysteine ligase with a function in glutathione biosynthesis, and two enzymes acting in glutathione metabolic processes, were upregulated. Lactoylglutathione lyase, essential in methylglyoxal detoxification, decreased. Toxic methylglyoxal, produced by different pathways such as threonine and glycine catabolism and lipid oxidation, affects DNA and proteins by the deletion and transversions of base pairs and the glucation of amino acid side chains in proteins, which leads to the malfunctioning of proteins. Methylglyoxal detoxification is, therefore, essential.

In plants, methylglyoxal was also reported to possess beneficial functions in remodeling cellular redox homeostasis and it influences plant growth and signal transduction [60]. However, two plant enzymes acting in methylglyoxal degradation were upregulated. An upregulation was further found in four glutathione-S-transferases and five enzymes involved in glutathione-related processes, including glutathione dehydrogenase I3SE57, which belongs to the ascorbate glutathione cycle.

#### 2.2.12. Oxidative Stress, Peroxidase, Superoxide Dismutase, and Flavonoids

Detoxification of ROS, when ROS production is out of balance, is a crucial step in counteracting ROS-caused damage [61]. Superoxide dismutase Q985K3 was the only upregulated symbiont enzyme for superoxide detoxification. The upregulation was significant, but FC was low. In the symbiont, no peroxidase increased in abundance. Two peroxidases were downregulated, one significantly (Q98AY3 cytochrome c peroxidase).

In the plant cells, three glutathione peroxidases and nine peroxidases were upregulated, many of them with significance. Four of them are secreted and others are sited to specific membranes and organelles. Another group of at least four peroxidases stayed unchanged, such as ascorbate peroxidase or a non-heme chloroperoxidase. Unchanged abundancy may, nevertheless, point to functions in coping stress. Two superoxide dismutase proteins increased, one stayed unchanged, and another one, localized to the extracellular space, was downregulated. Several significantly upregulated plant proteins are stress responsive or are associated with environmental stress reactions, for instance, plant ozone-responsive stress-related protein I3T2A1, plant peroxidase I3T056, a Bet v I/Major latex protein domain-containing I3SQK0 involved in stress response and the induction of PR genes (see plant hormones), or heme oxygenase 1 (I3SL57). Another functional class of plant proteins with upregulated members are enzymes involved in flavonoid biosynthesis, which is known to be triggered by biotic and abiotic stress including nutrient deficiency [62]. Isoflavone reductase A0A411P2Y4 and pterocarpan reductase Q05JY0, enzymes with functions in the biosynthesis of isoflavonoid phytoalexins, chalcone-flavanone isomerase 3 Q8H0F6, isoflavone 4′-O-methyltransferase Q84KK4, and chalcone-flavonone isomerase family protein I3STD3 belong to the proteins with increased abundance. Q985Y4, a protein with quercetin 2,3-dioxygenase activity, is insignificantly upregulated in the symbiont. The enzyme is involved in quercetin degradation. Quercetin is known to modulate the number of nodules in a concentration-dependent manner [63]. On the other hand, flavonoids are important for symbiosis. Special flavonoids induce the expression of rhizobial genes, such as the *nod* genes, which are required for nodulation. Phytoalexin flavonoids have been described to increase during nodulation [64]. Interestingly, a protein with similarity to nodE (Q98CY0) was slightly enhanced in the symbiont. The NodE protein is responsible for Nod factor decoration with polyunsaturated C18 fatty acid [65].

#### 2.2.13. Proteases

In the host cells, only a few proteases changed in abundance. Cysteine endopeptidase I3S747 was significantly upregulated, while I3SLH5 cysteine-type endopeptidase inhibitor was downregulated. Also, serine carboxypeptidase I3SYA1 was downregulated. Two proteins belonging to the ubiquitin-dependent protein catabolism increased. The increase in proteasome subunit beta was significant, while ubiquitinyl hydrolase B0BLB2 was slightly reduced. Secreted subtilisin-like protease 4 (A9QY38) was significantly upregulated, indicating that host–symbiont interactions are affected by goitrin-elicited processes as A9QY38 protease is required for arbuscular mycorrhiza development. Several subtilase genes are also responsive to Nod factor treatment [66]. Interestingly, Nod factor binding lectin-nucleotide phosphohydrolase Q9SPM8 was significantly increased.

In the microsymbiont, the proteolytic machinery is affected more strongly. Six serine proteases were upregulated, two of them significant, and one was downregulated. Serine type signal peptidase Q985A7 was downregulated. Two Clp proteases were affected; one was enhanced, and another one was reduced. Further upregulated proteases are metalloproteases, proline peptidase, oligoendopeptidase, carboxyterminal protease, and an ATP dependent protease. Aminopeptidase Q986E5 was significantly upregulated. Three proteins with functions in protease activity control were affected, one serine protease inhibitor was enhanced, a PepSy domain containing protease inhibitor was significantly upregulated, and protease regulator protein HflC (Q98KI9) was enriched.

#### 2.2.14. Miscellaneous Proteins

We want to only focus on two host proteins with functions in cytoskeleton organization. Significantly decreased I3T443 modulates the polymerization of actin, and insignificantly increased I2RZN5 acts in tubulin polymer formation. Thus, goitrin treatment may generate an imbalanced or misstructured cytoskeleton. The downregulation of profilin I3T443 may have fatal consequences as profilins are important for cell wall maintenance, nucleation, and cytokinesis and are, therefore, involved in many developmental processes [67]. Except for actin-binding, profilins bind to polyphosphoinositides [68].

### 2.3. Goitrin Impacts Growth and Proteome of Free-Living Mesorhizobium loti

#### 2.3.1. Death of Free-Living *Mesorhizobium loti*

**Figure 4:**
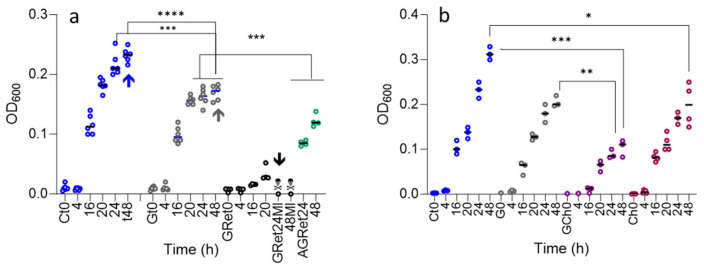
(**a**) Growth curves of *M. loti* in presence of goitrin (G) and control (C). Grey dots: goitrin-series (G); blue dots: control-series (C), n = 6. OD_600_: cell density, bars: mean values. Growth inhibition by goitrin is significant. The GRe-series presents a *M. loti* growth curve in the presence of 2.5 mg of goitrin using cultures after four previous serial goitrin incubation; n = 4. Validation of the microorganism identity of the culture after 24 or 48 h by PCR disclosed the death of *M. loti* (GRet24Ml, 48Ml), and confirmed the presence of a *Sphingomonas* species and *Ochrobactrum* species; after 24 and after 48 h, only *Ochrobactrum* species (AGRet24, 48). Blue and grey arrows: 48 h culture time points for proteome analyses. Black arrow, skulls: death of *M. loti* confirmed by PCR. Green symbols: growing *Sphingomonas* and *Ochrobactrum* species. (**b**) Growth curves of *M. loti* (purified stock) in the presence of goitrin (grey, G-series) and the control without goitrin (blue, C-series); growth curve of GCh-series: additive inhibition in the presence of goitrin and chicoric acid (dark purple); purple: growth curve of Ch-series: cultures with chicoric acid. Growth is significantly inhibited in the presence of chicoric acid and goitrin+chicoric acid. Significance: *p* < 0.0001 = ****; *p* < 0.001 = ***; *p* < 0.01 = **; *p* ≤ 0.05 = * (unpaired *t*-test).

The disturbed nodulation and nodule development after goitrin application lets us realize that the free-living bacteria are already damaged by goitrin. The growth curves of the free-living bacteria (stock BN154) in presence and without goitrin did not differ over the first day, while in the progressing cultures, growth retardation was unambiguous after 24 h. After 48 h, goitrin-supplemented cultures reached a cell density of maximal 80% of the controls (Figure 4). Additional growth curves were performed with bacteria obtained after four serial goitrin incubations. Inoculation of fresh medium containing 2 mg of goitrin with these cells and culturing them for 24 and 48 h led to almost no growth, but during the next two days, an increase in the cell density was stated. PCR studies disclosed that *M. loti* was no longer present in these cultures. The bacteria, which grew instead, were identified as a *Sphingomonas* species and a *Ochrobactrum* species. These species, undetectable by PCR checks of the stock 154 prior to culturing, were apparent contaminants. They are less sensitive to goitrin and, therefore, were favored during culturing; nevertheless, only the *Ochrobactrum* species finally survived.

#### 2.3.2. Decrease in Goitrin in the Culture Medium Is Concomitant with the Accumulation of Caffeic Acid Derivatives

In the presence of repeated the addition of 1mg and higher amounts of goitrin, the cultures already start to emit an extremely unpleasant odor after one day, while some odor development with 0.5 mM occurred later. Two new compounds accumulated in the culture medium, concomitant with the complete disappearance of goitrin. The new compounds were synthesized in high amounts when the incubation was performed with 2.0–2.5 mM goitrin, but their accumulation differed depending on the culture time. In cultures with 500 μM goitrin, traces of one compound were detectable after two days in the organic phase when the culture medium was extracted with EtOAc. The UV spectrum and the retention time of the new compounds point to caffeic acid derivatives. The identification of one of the compounds as caftaric acid was verified by LC-MS. The second compound was identified as chicoric acid. To our best knowledge, neither caftaric nor chicoric acid have been described as compounds produced by *M. loti*.

**Figure 5:**
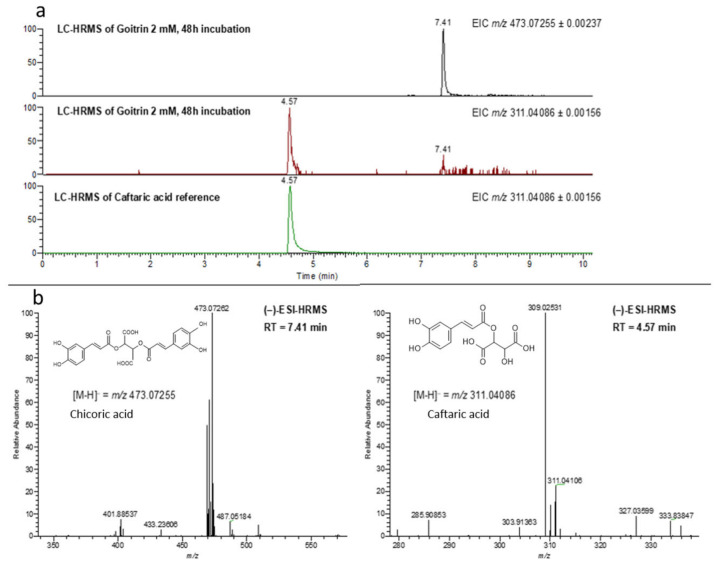
(**a**) LC-HRMS analyses of the goitrin incubation experiment revealing the presence of chicoric acid (EIC *m/z* 473.07255, RT = 7.41 min) and caftaric acid (*m/z* 311.04086, RT = 4.57 min) in the medium. (**b**) Mass spectra and compound structures.

The identification of caftaric and cichoric acid succeeded with the medium obtained after 48 h of incubation with 2 mM goitrin. Ultra-high performance liquid chromatography high-resolution mass spectrometry (LC-HRMS) equipped with an electrospray ion source was chosen to increase the grade of confidence (Figure 5). The analyses of the medium revealed the presence of peaks at 7.41 min corresponding to chicoric acid (C_22_H_17_O_12_, [M-H]^–^ = 473.07262, 0.18 ppm mass deviation, 100% relative abundance) and at 4.57 min corresponding to caftaric acid (C_13_H_9_O_9_, [M-H]^−^ = *m*/*z* 311.04109, 0.75 ppm, 22%). The presence of chicoric acid is supported by the formation of mono- and di-quinone derivatives oxidation products during the ionization process (*m/z* 471.05700, 0.22 ppm, 62%, and 469.04122, −0.06 ppm, 47%, respectively). Such oxidation reactions were also observed for the detection of caftaric acid from the medium (*m/z* 309.02531, −0.59 ppm, 22%) as well as from a standard solution (309.02501, −0.62 ppm, 100%; 311.04057, −0.93 ppm, 19%). No goitrin was detected in any of the samples containing high amounts of the caffeic acid derivatives, especially chicoric acid.

The precursor caffeic acid is a known allelochemical, and chicoric acid has been described to possess antimicrobial properties. The antibiotic properties of caftaric acid are insufficiently investigated. Therefore, we performed growth curves in the presence of caffeic, caftaric, and cichoric acid to elucidate the possible additive or synergistic inhibitory effects of these compounds. Chicoric acid (Figure 4b) inhibited the growth of *M. loti* in a similar range as found for goitrin. A combination of the compounds had a much stronger inhibitory effect. There was no clear influence of caffeic acid and caftaric acid as growth inhibition was not continuous during the course of incubation.

#### 2.3.3. Proteomes of Goitrin-Treated Free-Living *Mesorhizobium loti*

The proteome of *M. loti* under symbiotic conditions is known to differ considerably from the one of free-living bacteria [69]. Thus, different responses of the free-living *M. loti* were also expected upon goitrin exposure. The proteomes of two fractions F1 and F2 were compared. Fraction 1 consisted of pelletable cells at 2000 g and fraction 2 was the supernatant containing a composition of the secretome, dying and dead cells, and lysates. In total, 612 proteins were identified, with 590 in F1 and 219 in F2, due to some proteins found in both fractions. Comparisons of the group data (control vs. goitrin-treated) from three repetitions, disclosed up- and downregulated proteins. Significance was given with ≥log_10_ q values larger than 1.5. In fraction 1, 130 proteins were upregulated, 95 of them with significance, 298 proteins were downregulated, only three of them with significance, and 292 were unchanged or almost unchanged (specified range: 0.3 to −0.3 log_2_ FC, with exceptions). In fraction 2, 11 proteins were upregulated, only one of them significantly, 4 were unchanged, and 204 were downregulated, with 94 with significance.

**Figure 6:**
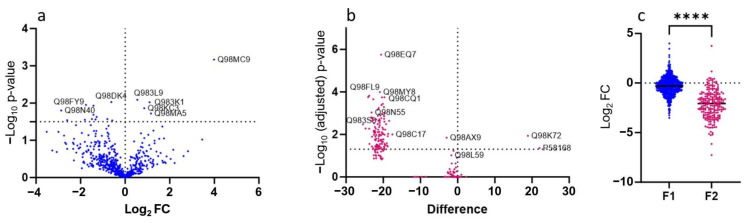
(**a**) A volcano plot of fraction 1 (F1, pelleted cells, blue dots) and of fraction 2 (F2, supernatant with secretome, dying and dead cells, red dots). The cultures were incubated with goitrin for 2 days. (**b**) The graph demonstrates the portions of lost proteins, almost unchanged proteins, and of two significantly increased proteins in F2 (Q98K72: translational regulatory factor EttA. P58168: bL19, binds to 23S rRNA; necessary for the assembly process of the 50S ribosomal subunit). (**c**) The boxplot illustrates the difference in protein abundancy of the pelleted cells (F1, blue) and F2 (supernatant, red) after goitrin incubation. Mean of F1: −0.2266; mean of F2: −0.8333; significance: *p* < 0.0001 = **** (unpaired *t*-test).

The proteomes of F1 and F2 displayed alterations triggered by goitrin treatment. Fraction 1 was less affected than fraction 2, which exhibited a dramatic loss of proteins in comparison to the control (Figure 6). As shown in more detail in Tables S18C–S25C, most affected proteins belong to chaperones, ribosomal proteins, transporters, proteins with functions in transcription and translation, and primary metabolism. Fold changes in F1 were mostly not significant after 2 days of incubation. Other functional classes, such as proteins involved in glutathione-related processes or proteolytic enzymes in F1 were less or almost not changed. Figure 6 depicts the decreased abundancy of goitrin-treated F1 proteins (versus the control) and the dramatic loss of protein abundancy in F2. Thus, both fractions are dominated by downregulations.

#### 2.3.4. Transcription

Eight proteins with functions in transcription were significantly downregulated in F2 (Table S18C). DNA-directed RNA polymerase alpha unit (Q98N33), a single-stranded DNA-binding protein, single-stranded DNA-binding protein Q98M41, transcription termination factor Rho (Q98DY8), and Q98MA8, a protein with an endoribonuclease domain, had the highest loss in abundancy. RecA (Q98NQ6), which is part of the SOS response system [70], tended to be downregulated in F1, but in F2, it was reduced with high significance. Six transcription-related proteins were considerably, but not significantly reduced, in contrast to the highly reduced Q98I77, a probable cold shock protein involved in stress tolerance and the regulation of gene expression. None of the identified proteins with functions in transcription were upregulated.

In F1, only two proteins were significantly reduced: 5′-nucleotidase Q98H62 and ribonuclease E (Q98NB6) acting in 5S and 16S rRNAs and the majority of tRNAs maturation and in mRNA degradation. Five proteins have an insignificantly lower abundance, among them, (anti)termination protein NusG, GMP synthetase, a protein involved in nucleoside triphosphate synthesis, and pseudouridine-5′-phosphate glycosidase.

The DNA repair protein RecN for double strand break repair (Q98KC3) and stress response transcriptional regulator Q983K1 were significantly increased. Five proteins were insignificantly increased, including the two-component system response regulator Q98CL8 for DNA-templated transcription, and Q988L2, another member of the two-component signal transduction system, which alters gene expression. Q988L2 belongs to the two-component signal transduction system, which enables bacteria to adapt to changed environments [71]. The results point to the stress-induced differential regulation of defined transcriptional processes and the effects on nucleotide metabolism in F1. However, dissection of the transcriptome profiles during goitrin treatment was not intended in this study.

#### 2.3.5. Ribosomal Proteins and Translation

In F2, all identified ribosomal proteins were reduced in abundance, except for the insignificantly increased bL19 (P58168) necessary for the assembly of the 50S ribosomal subunit. Eight proteins were reduced with significance (Table S19C), among them, asparagine, methionine, and phenylalanine-tRNA ligases. In F1, seven proteins of the large subunit and three proteins of the small subunit were reduced, but only uL1 was reduced significantly. Protein uL1 has a function in E site t-RNA release. Other ribosomal proteins (bS6, uS19, uS2, S9, uL15, and bL19) increased without significance. A third group was not or almost not affected (S4, S1, S8, S11, S3, and L21). Isoleucine- and valine-tRNA decreased, as did ribosome-binding ATPase YchF, which binds to the large subunit and to the ribosome-recycling factor. The ribosome-recycling factor Q98MB8, downregulated in both fractions without significance, releases ribosomes from the mRNA for the termination of protein biosynthesis. Three elongation factors stayed unchanged. Considering the multiple interactions of ribosomal proteins among each other and with rRNAs, the changed abundancies led to disturbed, imbalanced, or downregulated protein biosynthesis, as expected. Worthy of mention, the impacts on the moonlighting functions of ribosomal proteins, such as S1, S4, S9, S10, L2, L4, and L14, may occur [70]. Although located in the periplasmic space, downregulated spermidine/putrescine binding protein Q98JB0 indicates reduced translation and is, therefore, also mentioned here. Spermidine was recently described to be essentially required for efficient translational elongation and cell growth [72,73].

#### 2.3.6. Chaperones

In F2, four chaperones were significantly reduced: co-chaperonin GroES 4 (Q983S3), chaperonin GroEL 3 (Q98AX9), chaperone GrpE (Q98GQ5), and the DegP-like periplasmic serine endoprotease (Q98KJ1), possessing chaperone activity (Table S20C). SecB (Q98DU8), a chaperone with functions in preprotein transport, was insignificantly lowered in F1 and not detected in F2. The Sec system is one of the two major bacterial pathways for transports of unfolded proteins across the inner membrane [34,74]. Three chaperones were insignificantly changed in F1, Q98LE8 and Q98G68 were both reduced, and Q98ME5 was increased. In F2, Q98LE8 was insignificantly reduced, and Q98ME5 was significantly reduced while Q98G68 was not detected. Q98LE8, Q98G68, and Q98ME5 function as peptidyl-prolyl cis-trans isomerase plp (PPIases) by regulating protein folding at proline residues. Q98LE8 is also a trigger factor chaperone [75]. PPIases represent accessory proteins in multiprotein complexes, for instance in pre-ribosomal ribonucleoprotein complexes [76], and are involved in processes related to signal transduction, transcription, and translation, and probably contribute to protein secretion [77].

Reduced chaperone abundancy, even when in low ranges, may have essential implications as the correct folding and repair of proteins can be slowed down with consequences for subsequent protein–protein interactions. As an example, co-chaperonin GroES 4 interacts with heat shock proteins and ribosomal proteins L7/L12. L7/L12 act in the translation initiation, elongation, and termination processes. Disturbances in metabolic processes due to reduced chaperone GrpE, which must cooperate with chaperones DnaJ, DnaK, and GrpE for full folding competence, may be severe when some of the partners are diminished and improper ratios of the proteins occur [78]. Moreover, misfolded and damaged protein can be toxic [79].

#### 2.3.7. Transporter Proteins, Cell Wall and Outer Membrane Proteins

Transporters were one of the protein groups most affected by goitrin (Table S21C). Again, solute-binding proteins of the ABC transporters were predominantly affected. They belong to solute-binding protein family 1, 2, 3, or 5 and to the leucine-binding protein family, while the SBP superfamily encompasses at least 33 Pfam families [80]. After substrate binding, SBPs have been reported to stimulate signal transduction cascades [81]. 

In F1, 18 identified ABC transporter proteins were down regulated. Most affected proteins were Q98FL9, Q98BA6, Q98FA8, Q98MD4. Seven of these proteins act in amino acid transport, ten in sugar transport. Two transporters/transporter binding proteins were not changed in abundance, one is responsible for thiamine/iron transport, the second one functions in ribose transport. None of the transporter proteins was significantly changed, and except for polyamine transporter Q98JB0, no increased transporter protein was found. 

In F2, 26 transporters were downregulated. Fourteen of them were decreased with significance. Eight transporters with amino acid /peptide binding proteins, ten transporters with sugar binding proteins were affected. Moreover, phosphate transporter protein Q98FL2 was reduced. Transport of thiamine, betaine, ATP, iron, and polyamine is impaired by downregulated Q98JB0, Q98H18, Q983A7, Q98G45 and Q98NR5.

In F2, OMPs Q98KC1 and Q98NC0 were significantly reduced proteins (Table S21C). Q98KC1, the outer membrane protein assembly factor BamD, has functions in assembly and insertion of beta-barrel proteins. Beta-barrel proteins have many functions including ones in transport and signaling. The entire outer membrane protein assembly Bam complex consists of protein BamA, and the four lipoproteins BamB, BamC, BamD and BamE), [48,49,82,83,84]. Outer membrane protein assembly factor BamA, Q98MC3, is reduced but without significance. OMP Q98NC0 belongs to the DsbA family. DsbAs, part of the oxidative folding process in Gram-negative bacteria, are oxidoreductases that recognize a variety of protein substrates [50,85]. 

Reduced peptidoglycan-associated lipoprotein Q98F85 acts in cell division and outer membrane integrity. Also, Q98F84, another protein of the Tol-Pal system, is insignificantly reduced in F2. Members of Tol-Pal system are outer membrane lipoproteins belonging to the NodT family of the RND (Resistance-Nodulation-cell Division) type efflux systems [86]. These proteins work with an inner membrane ABC transporter ATPase and an adapter membrane fusion protein. Most members of this family are likely to export primarily small molecules rather than proteins, but are related to the type I protein secretion outer membrane proteins TolC and PrtF- Beta-lactamase. This protein, the reduced abundancies of BAM complex members and members of the DsbA family point to a loss of outer membrane integrity in F1 and F2.

#### 2.3.8. Primary Metabolic Processes

Diverse proteins acting in primary metabolic processes were affected (Table S22C). Nine proteins with functions in carbohydrate-related processes were lost in abundance and three of them were involved in glycolytic steps. The reduction in pyrophosphate-fructose 6-phosphate 1-phosphotransferase was significant. Endo-1,3-1,4-beta-glycanase ExoK increased. Q98N63, a protein necessary for galactose utilization, decreased. Two proteins with functions in methionine and branched amino acid biosynthesis were slightly upregulated in F1. Q9836B, a protein for the interconversion of serine and glycine was downregulated, as was aminotransferase Q98I67. Also, nitrogen regulatory protein Q98N18 was reduced. Q98M73, with a function in glycerophospholipid biosynthesis, Q98BH8, an enzyme for the introduction of *cis* unsaturation into fatty acids, and Q984T2, with a role in the type II fatty acid elongation cycle, decreased. Except for the TCA enzyme citrate synthase Q98MC9, which was significantly upregulated, and respiratory chain protein Q98KQ7, the affected proteins of the TCA cycle and those of the respiratory chain were insignificantly downregulated. In fraction 2, all proteins with a changed abundancy were reduced, most heavily, pyrophosphate-fructose 6-phosphate 1-phosphotransferase which catalyzes the first step of glycolysis, Q98MY8 for acetyl-CoA production, succinate-CoA ligase, and isocitrate dehydrogenase. Thus, glycolysis and the TCA cycle belong to the strongly affected pathways which results in a reduction in the synthesis of primary metabolites and a restricted energy production.

#### 2.3.9. ROS Detoxification and Miscellaneous Proteins

Regarding proteins assigned to ROS detoxification and to a group of miscellaneous proteins, six proteins in F2 displayed a significant loss in abundancies (Table S23C), among them, a putative antifreeze protein (Q98F90), which was also reduced in F1. Other proteins with a strong reduction were a kinesin-like protein, and flagellin for motility. Bacterial kinesin-like proteins co-localize with tubulin. The kinesin-like protein was also reduced in F1 where flagellin was not detected. For Q98EQ7, a protein with unknown functions possessing a putative LemA-like domain, exhibited some loss with high significance in F2. The LemA-like domain points to a transmembrane protein. Superoxide dismutase Q985K3 and catalase/peroxidase Q987S0 were almost unchanged.

#### 2.3.10. Glutathione/Sulfur-Related Proteins

In F1, two glutathione-S-transferases are almost not affected, and three were downregulated (Table S24C). Proteins Q98DE8 and Q98M60, which are involved in glutathione syntheses, are reduced, as are the proteins Q98N11 and Q98DP1 which act in cysteine biosynthesis. Lactoylglutathione lyase Q98NF2 was upregulated, protein Q98LU5 for the glutathione-dependent detoxification of formaldehyde was downregulated. All other identified proteins related to glutathione/sulfur metabolism were slightly downregulated or unchanged. None of the alterations were significant. Notably, 8 of the 18 proteins were molecular docking candidates for goitrin (see below). In F2, six of the proteins found in F1 were identified, (Q98N11 and Q98DP1 for cysteine biosynthesis, methionine adenosyl-transferase Q98BJ4, thiol-specific peroxidase Q985V8, and thioredoxins Q98MC9 and Q98E31). Thioredoxins Q98CM9 and Q98E31 were downregulated in both fractions, and in F2 with significance. In bacteria, thioredoxins serve as general protein disulfide oxidoreductases. Among their many functions, they act in oxidative stress responses [87].

#### 2.3.11. Proteases, Peptidases, and Proteinase Inhibitors

None of the 22 identified proteases and peptidases were significantly changed in F1. Two proteolytic proteins (a component of the Clp protease complex responsible for organizing specificity of protein degradation and a membrane peptidase) increased with an FC higher than 1, while in F2, only two proteolytic enzymes were found in low abundance. One of them tended to be upregulated, while the other one was reduced (Table S25C). The result indicates that proteolytic processes still occur in functioning cells and almost do not occur in the supernatant.

### 2.4. Summary of Up- and Downregulated Proteins in Nodules and Free-Living M. loti

**Figure 7:**
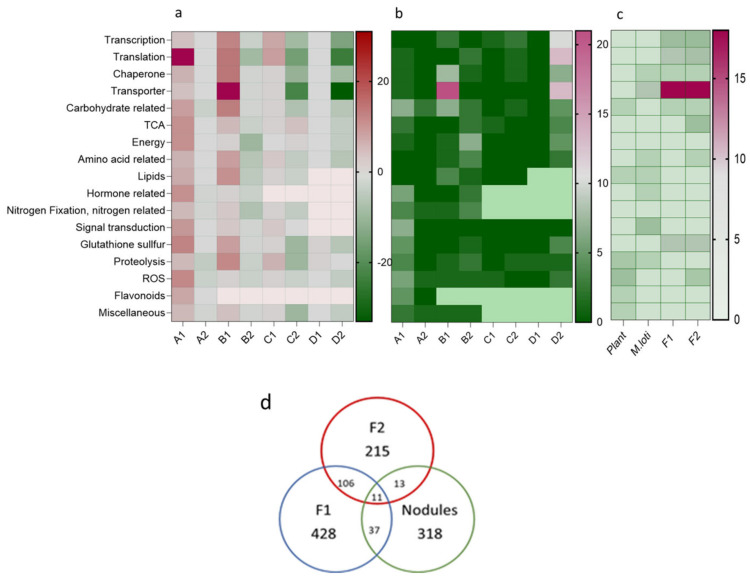
(**a**) Numbers of up- and downregulated proteins belonging to important functional classes preferentially affected by goitrin treatment. A: host; B: symbiont; C: cultured free-living *M. loti* (F1); D: culture supernatant (F2). 1: Upregulated proteins, 2: downregulated proteins. Bright cells: no protein present/inserted. Signal transduction: proteins with an assumed function in signal transduction are not included. (**b**) Corresponds a, but only the numbers of significantly changed proteins are pictured. (**c**) Illustrates the numbers of candidates for goitrin docking. Legend bars to the right of a, b, and c heatmaps: gradient red–green: number of proteins decreases from red to green; consider different scaling of the legend bars. (**d**) Venn diagram for the illustration of shared proteins.

The most striking differences in up- and downregulated proteins belonging to important functional classes of the F1, F2, and nodule proteomes are shown in Figure 7a,b. The host and symbiotic *M. loti* are characterized by upregulations and the free-living *M. loti* by the downregulations of proteins. The most affected proteins of the host are found in the functional classes related to hormones and signal transduction, glutathione, translation, carbohydrate, TCA, and energy. In the symbiont, the highest number of affected proteins are substrate-binding proteins of transporters, followed by proteins belonging to the functional classes of transcription, translation, chaperones carbohydrate-related processes, lipids, and proteolysis. The highest number of reduced proteins belong to the functional classes related to energy, nitrogen fixation, and translation. In the free-living *M. loti*, the largest number of downregulated proteins are involved in translation or are the substrate-binding proteins of transporters. The substrate-binding proteins of transporters are an outstanding group for goitrin docking followed by glutathione and translation-related proteins (Figure 7c). The summery illustrates the importance of the bacterial envelope and the substrate-binding proteins of transporters in toxic compound recognition. The Venn diagram presents the number of proteins with changed abundancy in the different proteomes and the shared occurrence of proteins in the proteomes (Figure 7d). According to our analysis, only very few affected proteins are found in all three bacterial proteomes. The nodules and F1 share only 37 proteins with altered abundancy, and with F2, 13 proteins are shared.

### 2.5. Molecular Docking Simulation Analysis

Molecular docking allows to predict the binding affinity of a ligand, here goitrin, to receptor proteins. For a rough estimation of goitrin binding to the defined sites of possible target proteins, we performed in silico molecular docking studies using SWISSDock [88], (Table S26). The results indicate considerable differences between the symbiotic and the free-living *M. loti* (Tables S1A–S14A, S17A, and S18C–S25C; Figures S1A,B and S2). The most candidates were found in the transporter group, for instance, amino acid binding proteins of the corresponding transporters, with eight in the free-living bacteria and four in the symbiont. The latter four candidates are proteins occurring in the symbiont and the free-living *M. loti* (Q98MK0; Q985D9; Q98CD0; and Q98EE6). Seven substrate-binding proteins of sugar transporters were identified as candidates in the free-living *M. loti*. Many candidates were proteins acting in glutathione-related processes with estimated ΔG values falling in the range of −7.35 to −6.65 kcal/mol, similar to the transporter proteins.

The most promising candidates are Q98EE6 (ABC transporter, amino acid binding protein), Q98LU5 (hydroxymethyl glutathione dehydrogenase), and Q98H18, (Tables S4A and S21C, Figure S1) existing in the proteomes of free-living *M. loti* and the symbiont. Q98H18 functions in the transport of small molecules (thiamine) and in the activation of signaling proteins including chemoreceptors and sensor kinases [81,89]. This protein could be regarded as a key protein in eliciting signal transduction responses during the early phase of goitrin exposure. The high number of candidates belonging to ABC transporters potentially designates the bacterial envelope as a dominant site of goitrin binding.

It is noteworthy that the binding energies for protein folding were higher, as observed for most of the tested proteins, (including ribosomal proteins and some chaperones). This suggests that goitrin is unlikely to form stable complexes with these proteins. However, we also considered proteins with estimated G values between −6.65 and −6.5 kcal/mol, as candidates for weaker, possibly temporarily existing docking complexes. Although unstable, they could, nevertheless, hinder protein functioning. Under this assumption, three identified amino acid-tRNA ligases in the free-living bacteria are weak candidates and two additional ones are stronger candidates for goitrin docking (Table S19C). A few candidates were found with proteins of the functional classes of transcription, primary metabolic processes, polyamine, drug efflux, and oxireductases. Except for glycolytic kinase Q98FJ1, none of the identified kinases, proteases, and peptidases identified in the free-living bacteria formed stable or weak complexes with goitrin (Tables S18C–S25C and S26), and only a component of the Clp protease (Q982V5) in the symbiont may complex with goitrin. The identified proteases seemed to not be inhibited by goitrin. Symbiont protein Q989L9, another interesting candidate, is a P450 monooxygenase involved in GA9 biosynthesis, with a ΔG value of −6.80 kcal/mol.

While the search for protein–goitrin complex formation emphasized on *M. loti* proteins, we identified only a few host proteins as docking candidates (Tables S1B–S17B). Within this group of plant proteins, a cysteine protease and three peroxidases have estimated ΔG values within the range of −6.95 to −6.72 kcal/mol. Additional candidates are annexin, profilin, subtilisin-like protease 4, and isoflavone reductase.

To note, changes in the protein abundancies did coincide in some cases with goitrin molecular docking simulations. The molecular mocking simulation analysis provided good hints for the formation of protein–goitrin complexes that inactivate proteins or hinder their functions otherwise.

## 3. Discussion

### 3.1. Consequences of Goitrin Poisoning in the Nodules

Reactive molecule species ROS, RNS, and RSS have important functions in nodule formation but also play a role in nodule senescence [90,91]. Oxidative stress elicited by goitrin treatment is regarded as a key initiator for dysfunctions of ROS homeostasis, activating ROS sensing systems and signal transduction cascades [92]. Although the microsymbionts are protected to some degree in the nodules from direct contact with goitrin, the proteome is, nevertheless, impaired, indicating that perhaps only a handful goitrin molecules are sufficient to elicit stress reactions in the partners. A highly increased abundancy of the two important *M. loti* chaperones GroES 2 and GroEL 2, points to an increased capacity for protein repair, but also to a forced modulation of NodD activity and the promotion of the nodulation process [93]. Essential functions of chaperones in nodulation are long known. For instance, when *Medicago sativa* was inoculated with groELc knockout mutants of *Ensifer meliloti*, a delay in nodulation was observed, leading to nodules being unable to perform N fixation [94].

The increase in chaperones GroES 2 and GroEL 2 in the large nodules is regarded as an attempt to increase stress tolerance, however, without success. The significantly upregulated *M. loti* superoxide dismutase and the many secreted host peroxidases indicate excessive ROS in the nodules, but a return to ROS homeostasis obviously failed. Nitrogenase activity is inhibited by ROS and together, with the downregulated energy production in the symbiont, nitrogen fixation is reduced, or not possible anymore. The consequences are downregulated proteins and an imbalanced protein composition of the N-fixation machinery. A stress-induced decline in N-fixation leads to premature nodule senescence [95]. In the nodules, a reduced abundancy of leghemoglobins was not noticed except for atypical leghemoglobin 2-1. However, the stress-induced reduction in N-fixation is known to occur prior to leghemoglobin decline [92,96].

In healthy symbiosis, the host cells suppress defense responses against the microsymbiont. Berrabah et al. assume unfavorable environmental conditions as reasons for a disturbed immunity/senescence balance, causing the loss of immunity suppression. The activation of defense responses in the host starts, among other reasons, with the upregulation of PR-10 gene expression and an increase in phenolic compounds [97]. In the proteome of the nodules from goitrin-treated plants, seven proteins belonging to family 10 of plant pathogenesis-related proteins (PR-10) and seven proteins with function in flavonoid/phytoalexin biosynthesis were upregulated, indicating that the suppression of defense reaction by the host was abandoned. I3S5I6, a protein possessing chitinase activity, was upregulated. Chitinase is regarded as a defense marker. On the other hand, the upregulation of PR-10 and chitinase gene expression were also assigned to senescence. [44]. Goitrin imbalances not only lead to ROS homeostasis but activate a defense reaction in the host cells, resulting in the abrogation of symbiosis and finally, nodule senescence is initiated. The induction of senescence processes is also indicated by the upregulation of host cysteine protease I3SZ47. The abscisic-acid-activated signaling pathway is most important in the goitrin response as indicated by the many upregulated stress- and defense-related proteins activated by ABA for the termination of symbiosis.

There are hints that *M. loti* unsuccessfully tries to counteract the imminent loss of the symbiotic existence. In the microsymbiont, mainly serine-type proteases were upregulated, but together with a significantly increased PepSY domain-containing protein. Proteins with a PepSY domain regulate proteases and prevent cell lysis. Many proteins with stress managing functions were upregulated, for instance, members of the two-component/phosphorelay signal transduction systems, or citrate synthase. LPS-assembly protein LptD acting in lipopolysaccharide assembly was significantly upregulated. LPS structures can trigger the host immune response. An increase in IAA-Ala conjugate hydrolase activity for auxin release and in components for Nod factor biosynthesis, point to attempts to stimulate the plant for a positive response and nodulation-related activities. The fight for survival of the microsymbiont, the increasing defense reaction in the host, and the reorganization of the metabolism are probably the reasons for the enhanced protein biosynthesis, preferentially in the host but to a lower degree, also in the microsymbiont. The excessive upregulation of many *M. loti* transporters indicates either highly enhanced export and import activities related to starting senescence, or the higher abundance is due to an increased biosynthesis to substitute damaged transporters.

The question of what the primary targets of goitrin binding are, is difficult to answer. We suggest that the cell walls of the host and the microsymbiont envelope function as first targets. To note, the presented molecular docking study considers hydrophilic, hydrophobic, van der Waals, electrostatic, and hydrogen bonding. Covalent adducts with isothiocyanates via cysteine have been described for trypsin-digested purified eukaryotic tubulin previously treated with several ITCs but not with goitrin. Also, the intact glucosinolates glucobrassicin and epiprogoitrin have antimicrotubular activity [98]. We identified significantly downregulated profilin, a protein that binds to actin, as a putative plant candidate for goitrin complex formation, while tubulin was not affected. It is unclear whether covalent adduct formation with goitrin affects the proteolysis of some of the complexed proteins. This point needs further investigation.

Free goitrin was not yet detected in the nodules. Here, detoxification via glutathione conjugation cannot be excluded, as diverse host and some microsymbiont glutathione-S-transferases were upregulated. However, many of these proteins are candidates for goitrin docking. The search for goitrin–glutathione conjugates and their possible derivates was not the aim of this study.

### 3.2. Goitrin Damage in Free-Living Mesorhizobium loti

Regarding the proteomes of the free-living bacteria directly exposed to goitrin, we found a considerably stronger downregulation of proteins compared to the symbiont. The protein interactions of impaired proteins may potentiate implications due to knock-on effects, even when the fold changes of single proteins were low or not yet significant. As presented above, the most affected functional classes are chaperones, ribosomal proteins, transporters, and proteins related to transcription and translation. Thus, the entire metabolism is impaired by goitrin with consequences for viability, compound transport, membrane integrity, cell division, and the mobility of *M. loti*.

The highest up- and downregulated proteins in F1 may give hints to key metabolic responses. The damage to outer membrane integrity and the loss of the proper function of transporters is certainly a key event. Membrane integrity is of outstanding importance for survival. Recently, the damaged proteins of the periplasm, cell wall, and outer membrane were found to increase the death rate in starving *E. coli* [99]. Since proteins localized to the cell envelope are among the most affected proteins, we assume that continuing exposure to goitrin preferentially damages the bacterial envelope and membrane integrity. We hypothesize that the affected transporter proteins elicit subsequent deleterious events in the cells, most likely attributed to ROS. Surprisingly, superoxide dismutase was almost unchanged in F1.

Dysfunction in the TCA cycle is another key event. Highly increased citrate synthase Q98MC9 and the downregulation of succinate-CoA ligase subunit alpha may have indicated TCA dysfunction or initiated metabolic breakdown. Citrate activates the glyoxylate cycle and inhibits the bacterial tricarboxylic acid cycle. Citrate synthase is a moonlighting enzyme not only with functions in the central energy metabolism, but it acts also in cell cycle regulation [100]. Recently, an increase in citric acid was described to be related to antibiotic tolerance, concomitant with a reduced energy metabolism [101]. Respiratory chain NADH-quinone oxidoreductase subunit C (Q98KQ7) was upregulated. The reason for the high upregulation of Q98KQ7 is presently unclear. A function in antioxidant defense, such as reducing quinone substrates or H_2_O_2_, is only known for water-soluble quinone oxidoreductases [102]. The significantly downregulated ribonuclease E (Q98NB6) for t-RNAs maturation and mRNA degradation together, with the imbalance in ribosomal proteins encloses malfunctions in translation processes.

The many unchanged or downregulated glutathione-S-transferases being candidates for goitrin complex formation and other proteins involved in glutathione-related processes would be concordant with impossible goitrin–glutathione conjugate formation. Many isothiocyanates form covalent adducts with proteins, such as glutathione-S-transferase, and with nucleic acids. According to Kumari et al. [103], proteins are the dominant targets for covalent adduct formation with ITCs. While the strongest binding sites are ionized thiol groups of cysteine; binding to lysine, arginine, proline, serine, threonine, and tyrosine occur as well.

We found differences and similarities of goitrin-stressed and general stress responses known to occur in bacteria. Known stress responses in bacteria encompass a reduction in translation, an inhibition of the synthesis of ribosomal proteins, ribosomal RNA down regulation, ribosome silencing, and ribosome hibernation [34]. However, when exposed to goitrin, only the reduced abundancy of many ribosomal proteins and some AA-tRNA ligases are in line with known stress reactions. The reduced abundancy of ribosomal proteins and the loss of the proteins necessary for energy production in goitrin-treated *M. loti*, seem more related to imminent death. Thus, many important strategies for coping stress are surrendered. The failure of stress adaption is accompanied by extensive proteolytic processes in the cells and leads to the dramatical loss of proteins in fraction 2.

It is doubted that *M. loti*, when impacted by goitrin, can perform normal invasion and nodule formation. As discussed by Simms and Taylor, plants avoid establishing a mutualistic interaction with microorganisms that suffer from reduced fitness [104].

### 3.3. Are Caffeic Acid Derivatives Linked with Goitrin Disappearance?

The malodorous volatile compounds in the bacterial cultures are produced during goitrin disappearance and the accumulation of caffeic acid derivatives. A goitrin-Cys-Gly conjugate or related derivatives could not be identified. To our best knowledge, a pathway for complete goitrin degradation is presently unknown. Therefore, a possible pathway in goitrin degradation is discussed in more detail.

As mentioned in the introduction [25], goitrin can be converted to the corresponding 1,3-oxazolidine-2-one in presence of H_2_O_2_. The conversion to 1,3-oxazolidine-2-one, concomitant with the release of a sulfur compound, probably H_2_S, requires intermediate ring opening. It is possible that complete goitrin degradation starts from an unstable, open-ring intermediate, while the breakdown products could again be bioactive molecules, such as H_2_S or mercaptans. In this case, only a small portion is finally converted to the corresponding oxazolidine-2-one, if at all. This compound was undetectable in the culture media.

**Figure 8:**
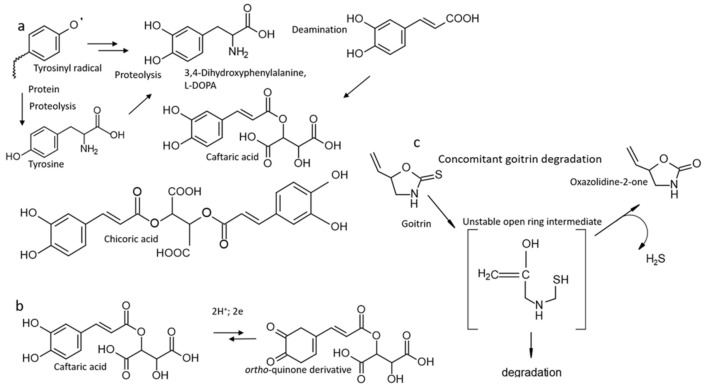
(**a**) Hypothetical formation of caftaric acid starts from the protein-bound tyrosinyl radical, which is converted to protein-bound L-DOPA, released by proteolysis. Tyrosine can also directly be released by proteolysis. Aspartate transaminase may catalyze the production of caffeic acid from L-DOPA. Conjugation of caffeic acid with tartrate yields caftaric acid and the esterification of caftaric acid with a second caffeic acid leads to cichoric acid. (**b**) For instance, the oxidation of caftaric acid results in an *ortho*-quinone derivative, which acts as a pro-oxidant agent. (**c**) Reductive ring opening of goitrin may result in an unstable intermediate as a starter molecule for complete degradation, or to the conversion to oxazolidine-2-one in an oxygen-rich environment.

Caffeic acid, caftaric acid, and cichoric acid, where the latter is esterified with a second caffeic acid molecule, have antioxidant activity while oxidized to *ortho*-quinone products [105]. The released electrons can be used for reductions such as reductive ring opening, for instance, of the goitrin heterocycle (Figure 8). When the compounds turn to pro-oxidant agents, the initial beneficial, damage reducing effects of caftaric and chicoric acid shift to fatal ones, causing lipid peroxidation and DNA damage. When turning to pro-oxidant agents, the compounds acquire antibiotic properties and could accelerate the death of *M. loti*. If the oxidation of caftaric acid is reversible, for instance by cellular constituents with reducing properties [105], the mode of action of the derivatives may oscillate. This scenario needs further investigation, as well as the origin of the caffeic acid derivatives.

Although speculative, we would like to present the possibilities of caftaric and cichoric acid formation. The unexpected presence of these compounds, accumulating during goitrin disappearance, requires the production of the precursor caffeic acid, which could be provided by tyrosine/phenylalanine conversion. However, phenylalanine ammonia lyase PAL and tyrosine ammonia lyase TAL exist only in a few bacteria. Neither TAL nor PAL were found in the *M. loti* proteomes. In the F1 proteome, aspartate transaminase Q98H83 was also identified, but the enzyme was downregulated. Bacterial aspartate transaminase was recently described to possess a broad substrate specificity, including TAL and PAL activity [106]. Thus, the conversion of tyrosine and phenylalanine to the corresponding phenolic compounds does not necessarily depend on TAL and PAL.

We preferentially assume another rather interesting possibility without the necessity of a further hydroxylation step by producing caffeic acid via DOPA (3,4-dihydroxyphenylalanine). In bacteria, protein-bound tyrosine is converted to phosphotyrosine, which occurs upon H_2_O_2_ stress [107]. Following this, phosphotyrosine (p-Tyr) is H_2_O_2_ dependently oxidized by a peroxidase, and converted to protein-bound 3,4-dihydroxyphenylalanine (DOPA). This process consumes H_2_O_2_. DOPA could be released by proteolysis. Free DOPA could be immediately converted into caffeic acid by aspartate transaminase. The suggested pathway still has to be proved. Also, the origin of tartaric acid is presently unclear. Recently, dihydrofumarate was assumed to function as an intermediate for tartrate production [108]. Also, tartrate generation from ascorbate breakdown is discussed [109].

The contaminating species are assumed to be responsible for goitrin degradation and the synthesis of caffeic acid derivatives. The *Shingomonas* and *Ochrobactrum* species exist in soil as endophytes in plants and the rhizosphere, but also occur as subsidiary members of nodule microbiomes. The species composition of the microbiome is known to change during the lifetime of the nodule [110,111,112,113]. Members of the accessory microbiome of the nodules have been recently recognized as producers of specialized metabolites with antibiotic and other properties [114].

### 3.4. Relevance in Agriculture

Glucosinolate-derived degradation products belong to the few allelochemicals with visible impacts on natural plant communities. In agricultural systems, the Brassicacea species are used as biofumigants to reduce fungal pathogens and root–knot nematodes in the soil [115,116]. The intercropping of legumes with crop species, such as wheat or potato, is a common practice in agriculture to increase the N availability in the soil and to reduce the use of mineral fertilizers. In most studies, only the positive effect of the legume on the yield of cereals was considered. Shanmugam et al. studied N use efficiency of faba bean and cabbage (*Brassica oleracea* var. *capitata* cv. *Conica*) grown in monocropping and intercropping systems [117]. Faba bean pod yield was lower in the intercropping system, but cabbage productivity increased. No information is given about faba bean nodulation. In another study, *Brassica napus* var. Boheme was grown in mixture systems with different legumes (lupine, vetch, and clover). The harvest was after three months of growth. The dry weights of all three legumes were significantly lower in the intercropping systems [118]. Nodulation of the three legumes was not addressed in this study. These and other comparable studies indicate a negative effect of *Brassica* species on legumes when intercropped but the reasons are unclear. Allelopathic interaction, including goitrin as one causative agent that negatively influences nodulation, should be taken into consideration.

It is noteworthy that goitrin is not only toxic to certain microorganisms, but also to mammals because of the strong antithyroid activity. Goitrin inhibits thyroid peroxidase, lactoperoxidase, tyrosine iodination, and thyroxine (T4) formation [119,120]. The colonic microbiome in mammals can contribute to the degradation of progoitrin, as do some *Lactobacillus* spp. and a strain of *Enterobacterium* spec. [121]. Moreover, the thyreostat 2-thiouracil was identified after Brassicaceae crop feeding in the urine and feces of livestock and of humans. 2-Thiouracil production, formed by the gut bacteria *Lactobacillus reuteri* and *E. coli* strains, seems to be related to glucosinolate degradation products, and may present a bacterial stress reaction.

As a bottom line, goitrin is a powerful metabolite of the Brassicaceae. The compound potentially modulates plant species composition in the neighborhood via the suppression of the Rhizobiaceae species and other microorganisms.

## 4. Materials and Methods

### 4.1. Plant Culture

*Lotus japonicus* seeds were collected in a 1.5 mL microcentrifuge tube and 1 mL of sterilizing solution (12% NaOCl 2% (*v*/*v*), Tween 20 0.01% (*v*/*v*)) was added. After 20 min of incubation under gentle agitation, the sterilization solution was discarded, and the seeds were rinsed five times with sterile dH_2_O. Washed seeds were kept in 1 mL of sterile dH_2_O in the dark, overnight, at room temperature. The following day, the seeds were placed on water agarose plates under sterile conditions and kept for 24 h at 4 °C for stratification. Following this, the plates were exposed to light at 24 °C for 12 days. The seedlings were cultured in pots (diameter of 15 cm) and filled with quartz sand. Previously, the quartz sand had been washed 10 times with tap water followed by 10 additional washing steps with dH_2_O. The autoclaved sand was distributed in pots and three seedlings/pot were planted. Over the first days of culturing, the trays were covered with a transparent hood. Trays were placed in a phytochamber with 160 μmol m^−2^ s^−1^ light, 25 °C, and in 65% humidity. Plants were watered every 2 days with 40 mL of dH_2_O. After the first week of growth, the plants were also watered once a week with 0.05 P/5N fertilizer (40 mL/pot) to prevent wild nodulation with *Rhizobia*. After 8 weeks of growth, the plants were inoculated with *Mesorhizobium loti* WT strain Lj7a (stock BN125). Further fertilization was without N.

### 4.2. Sequencing of Mesorhizobium loti Stock BN125 and BN 154

The identity of the microorganisms in stocks BN125 and BN154, previously isolated from *L. japonicus* nodules developed after inoculation with WT strain Lj7a, was confirmed prior to culturing and inoculation by sequencing. The bacterial 16S rRNA V4–V7 region was amplified by colony PCR. The primer of 16S rRNA V4–V7 region (799F: AACMGGATTAGATACCCKG and 1992R: ACGTCATCCCCACCTTCC) is described by Schütz et al. [122]. PCR products were purified and sequenced by Sanger sequencing. In the stocks, *Mesorhizobium loti* was the only bacterium identified by this method. However, rare contaminants can be overseen even if DNA high throughput culture sequencing is performed [123].

### 4.3. Cultures Mesorhizobium loti and Inoculation of Lotus japonicus

For the inoculations and proteome studies, the *M. loti* stock BN125 was used. Three-day-old *M. loti* cultures grown in TY medium were diluted to a final OD_600_ of 0.05 with dH_2_O. Inoculation of 8-week-old plants was performed with 40 mL of the diluted cultures. Control plants were watered without *M. loti*. One week after inoculation, the plants were treated with 40 mL of 0.5 mM (860 μg/treatment) goitrin (Santa Cruz Biotechnology, Dallas, TX, USA) every three days for four weeks (total dosage, 7.74 mg goitrin within 4 weeks). After the treatment, the plants were harvested, washed with dH_2_O, their phenotypes were documented, and harvestable nodules were collected with forceps, and transferred to ice-cold 1.5 mL caps. The nodules were frozen in liquid nitrogen and stored at −80 °C for further analysis.

### 4.4. Cultures of M. loti Stock BN154 with Goitrin, Caftaris Acid, and Chicoric Acid

Growth in TY medium was monitored for 2 days. A total of 50 μL of a preculture was used to inoculate flasks containing 15 mL of TY medium. Serial goitrin incubations were performed by transferring a 100 μL aliquot of a two-day-old goitrin culture to fresh medium, which was used after 2 days as a source for the next incubation. The series encompassed four subsequent cultures. Pelleted cells from culture 4 were resuspended in fresh TY medium to an OD_600_ of 0.05 and incubated with 500 μM and 2.0–2.5 mM goitrin. The high concentrations of goitrin were used for the identification of new compounds.

The bacteria growing after serial incubation were identified as *Sphingomonas* sp. and *Brucella* sp. by search against the NCBI Nucleotide database using BLASTN. The Gene-Bank accession number of *Spingomonas* sp. is PQ299522, and of *Brucella* sp., PQ299523 (NCBI Nucleotide database).

Additional growth studies with goitrin, caffeic acid, caftaric acid, and chicoric acid were performed with *M. loti* from stock BN154. The stock was again purified before use. For purification, cells were repeatedly plated on TY agar, and grown for days at 27 °C. Single colonies were picked, cultured, and used for PCR. The PCR products were purified and sequenced as described above. As before, only *Mesorhizobium loti* was identified (accession number PQ299524). Cultures of the purified stock were incubated with chicoric acid, caffeic acid, or caftaric acid and with mixtures of chicoric acid/goitrin, caftaric acid/goitrin, and caffeic acid/goitrin. Bacterial growth was monitored for two days. The compounds were added at the start of culturing.

### 4.5. Identification of Caftaric Acid and Cichoric Acid

Because of the instability of the thermolabile compounds, the supernatant of the BN154 goitrin cultures was used for compound identification after sterile filtration without further processing.

Liquid chromatography was performed on a Thermo Fisher UltiMate 3000 UHPLC (Waltham, MA, USA) built from a binary RS pump, an XRS open autosampler, a temperature-controllable RS column department, and a diode array detector. Sample separation was achieved at 30 °C on an ACQUITY UPLC CSH column (2.1 × 100 mm, 1.7 μm particle size). Eluent A consisted of H_2_O and eluent B consisted of acetonitrile; both were acidified with 0.1% formic acid. The following gradient was applied at a constant flowrate of 0.45 mL: (i) 5% B isocratic until 0.0–1 min; (ii) linear increase to 30% B until 6 min; (iii) ramping to 100% until 9.0 min; (iv) holding 100% B until 11.0 min; (v) change until 11.1 min to the starting conditions of 5% B; and (vii) equilibration for 2.9 min resulting in a total flow time of 14 min until the next measurement run.

The mass analysis was conducted on a Thermo Fisher Scientific Q Exactive hybrid quadrupole-Orbitrap mass spectrometer (Waltham, MA, USA) equipped with a heated ESI source at position B in the negative ionization mode and a voltage of 3.5 kV. Sheath, auxiliary, and sweep gas (N_2_) flow rates were fixed at 50, 13, and 3 (arbitrary units), respectively. The capillary temperature amounted 263 °C and the auxiliary gas heater temperature was 425 °C. The S-lens RF level was set at 55.0. Mass spectra were acquired in the mass range between *m*/*z* 50 and 750, 70,000 resolutions at *m/z* 200, and with an AGT target of 1e6. The mass analyzer was calibrated for mass accuracy once a day according to the manufacturer’s instructions.

### 4.6. Protein Extraction and Proteomics

*M. loti* BN125 (500 μL of a pre-culture in TY, OD600 = 0.1) was cultured in TY medium in the presence and absence of 500 μM goitrin (control) for two days at 28 °C (final ODs between 0.15 and 2.0) and 100 rpm. For protein extractions, 9 cultures were sampled, resulting in one sample. The combined cultures were centrifuged at 2000× *g*. The pellet, suspended in 1 mL of culture medium, (fraction 1) and the supernatant (fraction 2), were divided for obtaining three samples used for protein extraction and proteomic analyses.

The harvested nodules from the control and goitrin-treated plants (see above) were grinded with 600 μL of lysis buffer/10 mg nodules using a Polytron homogenizer. The homogenate was centrifuged (500× *g*, 4 °C for 5 min) and the proteins were purified by chloroform/methanol precipitation as described by Wessel and Flügge [124]. The final pellet was washed with ice-cold methanol and centrifuged. The further procedure was performed according to Tatsukami et al. [69]. *Mesorhizobium loti* cells were extracted, and the samples prepared as described by Tatsukami et al. [69].

For MS analysis of the samples, 1 μg of the desalted samples were loaded onto a C18 reverse phase capillary trap column (Acclaim PepMap C18, 75 μm × 2 cm, 3 μm particle size, ThermoFisher, Langerwehe, Germany) and separated using a C18 reverse phase analytical column (Acclaim PepMap C18, 75 μm × 25 cm, 2 μm particle size, 100 Å, ThermoFisher, Langerwehe, Germany) coupled to an UltiMate3000 nano RSLC system (Thermo). A 90 min gradient from 5 to 32% (*v*/*v*) acetonitrile 0.1% formic acid in H_2_O was used to elute the peptides at a flow rate of 300 nL min^−1^. The nano-LC system was on-line coupled to an Impact II high-resolution quadrupole-time of flight tandem mass spectrometer using a CaptiveSpray nano-electrospray source [125]. The MS spectra were acquired at a range from *m/z* 200 to 1750 at 4 Hz. For fragmentation, the 17 most intense precursor ions of each MS scan were selected (Top 17 method). MS/MS spectra of the fragmented precursor ions were recorded in a mass range from *m/z* 300 to 1750 at an intensity-dependent collection rate of 2 to 20 Hz spectrum.

Peptides were identified and quantified using the MaxQuant software package, version 1.6.10.43 [126] using the Andromeda search engine [127]. Generic settings for Bruker Q-TOF instruments were used to match spectra to protein sequences to the *Mesorhizobium japonicum* reference proteome uniprot-proteome_UP000000552_Mesorhizobium _japonicum. fasta [128], and *Lotus japonicum* reference proteome [129]. Search parameters included precursor mass tolerance of ±10 ppm, fragment ion mass tolerance of ±20 ppm, and semi-tryptic peptides with up to one missed cleavage; static modifications were cysteine carboxyamidomethylation (+57.02 Da) and methionine oxidation (+15.99 Da). A false discovery rate of 0.01 was applied both for spectrum-to-sequence matching and protein identification.

### 4.7. Molecular Docking Analysis

Protein structures were downloaded as pdb-files from either AlphaFold [130,131] or MODBASE [132]. Docking was performed using SwissDock in accurate mode but without any flexibility option [33]. As a ligand, the ZINC ID of R-Goitrin (ZINC5226611) was inputted via the HTML Interface of SwissDock for all queries. The exact parameters of each query were as follows: PASSIVEFLEXIBILITYDISTANCE = 0.0, WANTEDCONFS = 5000, NBFACTSEVAL = 5000, NBSEEDS = 250, SDSTEPS = 100, ABNRSTEPS = 250, CLUSTERINGRADIUS = 2.0, MAXCLUSTERSIZE = 8. Docking results were viewed and evaluated using UCSF Chimera [133], and developed by the Resource for Biocomputing, Visualization, and Informatics (University of California, San Francisco). It is worth noting that SwissDock predicted ligand binding sites overlapped with experimentally determined native binding sites in 64% of docking experiments [131].

### 4.8. Determination of Gibberellin GA4 and GA9 in the Nodules

Extraction of the gibberellins GA4 and GA9 from 50 mg nodules was performed as described by Tatsukami and Ueda [52]. Following extraction, phytohormones were dissolved in 0.1 mL of methanol/H_2_O (1:1, *v*/*v*, with 0.1% formic acid) and then subjected to separation on a reverse-phase C18 Gemini HPLC Column (5 μm particle size, 150 × 2.00 mm, Phenomenex) for analysis using HPLC-ESI-MS/MS. The quantification of phytohormones through mass spectrometry utilized a QTRAP 6500+ LC-MS/MS system (Sciex, Darmstadt, Germany) equipped with a Turbo V ion source and an Agilent 1260 Infinity quaternary pump. Detection of phytohormones occurred in the negative ion mode, employing specific instrument settings: curtain gas at 25 psi, collision gas at medium, ion spray voltage at −4500 V, ion source temperature at 150 °C, and nebulizer and heater gas at 25 psi. The parameters for multiple reaction monitoring (MRM) were as follows: declustering potential at −80 V, entrance potential at −10 V, cell exit potential at −8 V; collision energies were optimized for each phytohormone (Table S27). Data analysis was performed using MultiQuant 3.0.2 software. Identities of GA4 and GA9 were confirmed with RT and MRMs of pure commercial standards.

### 4.9. Statistics

Data was used from Tables S1–S19. Volcano plots and boxplots were analyzed by multiple unpaired *t*-tests for significance using GraphPad Prism version 10.1.2. Further methods are directly mentioned in the text.

## 5. Conclusions

The findings demonstrate the toxic potential of cyclic isothiocyanate goitrin derived from progoitrin, a wide-spread glucosinolate in Brassicaceae. Free-living *M. loti* are severely damaged by the compound. In already existing nodules, the symbiotic lifestyle is destroyed and injured free-living bacteria are unable to perform proper nodulation. Under natural conditions, the survival of *M. loti* certainly depends on co-existing microorganisms that are competent in degrading goitrin into non-toxic and short-lived products. This underlines the importance of a high microbial diversity in the soil and healthy soil microbiomes. Other microorganisms, even members of the endo-microbiomes, respond with the production of antibiotics. The sustainable use of allelopathy in agriculture demands much more knowledge of the complicated interactions between organisms on the molecular level for developing sophisticated culture systems.

## Data Availability

The datasets generated and/or analyzed during the current study are available from P.H. and the corresponding author upon reasonable request.

## Supplementary Materials

The following supporting information can be downloaded at: https://www.mdpi.com/article/10.3390/plants13202897/s1. Tables S1A,B–S17A,B series: Goitrin treated large nodules versus control nodules. Tables S18C–S25C: Fold change (FC) of down and up regulated proteins in fraction 1 (F1) and fraction 2 (F2). Table S26: -ΔG values of Proteins (ID) Goitrin Docking Analysis. Table S27: MRM parameters for phytohormones. Figure S1: Interaction site of R-goitrin(ligand) and target proteins Q98EE6, Q98H18, and Q98LU5. Figure S2: GA9 contents in the large nodules (50 mg) and nodules of the control plants (50 mg).

## References

[1] Velasco P., Cartea M.E., Gonzalez C., Vilar M., Ordas A. (2007). Factors affecting the glucosinolate content of kale (*Brassica oleracea acephala* group). J. Agric. Food Chem..

[2] Lankau R.A., Nuzzo V., Spyreas G., Davis A.S. (2009). Evolutionary limits ameliorate the negative impact of an invasive plant. Proc. Natl. Acad. Sci. USA.

[3] Trejo-Téllez L.I., Estrada-Ortiz E., Gómez-Merino F., Becker C., Krumbein A., Schwarz D. (2019). Flavonoid, nitrate and glucosinolate concentrations in brassica species are differentially affected by photosynthetically active radiation, phosphate and phosphite. Front. Plant Sci..

[4] Jiang Y., Li J., Caldwell C.D. (2016). Glucosinolate Content of *Camelina* Genotypes as Affected by Applied Nitrogen and Sulphur. Crop Sci..

[5] Pant B.D., Pant P., Erban A., Huhman D., Kopka J., Scheible W.R. (2015). Identification of primary and secondary metabolites with phosphorus status-dependent abundance in *Arabidopsis*, and of the transcription factor PHR1 as a major regulator of metabolic changes during phosphorus limitation. Plant Cell Environ..

[6] Frerigmann H., Piotrowski M., Lemke R., Bednare P., Schulze-Lefert P. (2021). A Network of Phosphate Starvation and Immune-Related Signaling and Metabolic Pathways Controls the Interaction between *Arabidopsis thaliana* and the Beneficial Fungus *Colletotrichum tofieldiae*. Mol. Plant-Microbe Interact..

[7] Jeon B.W., Oh M.-H., Kim H.S., Kim E.O., Chae W.B. (2022). Glucosinolate variation among organs, growth stages and seasons suggests its dominant accumulation in sexual over asexual-reproductive organs in white radish. Sci. Hortic..

[8] Hofmann D., Thiele B., Siebers M., Rahmati M., Schütz V., Jeong S., Cui J., Bigler L., Held F., Wu B. (2023). Implications of Below-Ground Allelopathic Interactions of *Camelina sativa* and Microorganisms for Phosphate Availability and Habitat Maintenance. Plants.

[9] Plaszkó T., Szúcs Z., Vasas G., Gonda S. (2022). Interactions of fungi with non-isothiocyanate products of the plant glucosinolate pathway: A review on product formation, antifungal activity, mode of action and biotransformation. Phytochemistry.

[10] Vaughn S.F., Boydston R.A. (1997). Volatile Allelochemicals Released by Crucifer Green Manures. J. Chem. Ecol..

[11] Cipollini D. (2016). A review of garlic mustard (*Alliaria petiolata*, Brassicaceae) as an allelopathic plant. J. Torrey Bot. Soc..

[12] Lankau R.A. (2011). Resistance and recovery of soil microbial communities in the face of *Alliaria petiolata* invasions. New Phytol..

[13] Lankau R.A. (2012). Interpopulation variation in allelopathic traits informs restoration of invaded landscapes. Evol. Appl..

[14] Hansen J.C., Schillinger W.F., Sullivan T.S., Paulitz T.C. (2020). Decline in soil microbial abundance when camelina introduced into a monoculture wheat system. Front. Microbiol..

[15] Hansen J.C., Schillinger W.F., Sullivan T.S., Paulitz T.C. (2019). Soil microbial biomass and fungi reduced with canola introduced into long-term monoculture wheat rotations. Front. Microbiol..

[16] Siebers M., Rohr T., Ventura M., Schütz V., Thies S., Kovacic F., Jaeger K.-E., Berg M., Dörmann P., Schulz M. (2018). Disruption of microbial community composition and identification of plant growth promoting microorganisms after exposure of soil to rapeseed derived glucosinolates. PLoS ONE.

[17] Hu P., Wu L., Hollister E.B., Wang A.S., Somenahally A.C., Hons F.M., Gentry T.J. (2019). Fungal community structural and microbial functional pattern changes after soil amendments by oilseed meals of *Jatropha curcas* and *Camelina sativa*: A Microcosm Study. Front. Microbiol..

[18] Portales-Reyes C., Van Doornik T., Schultheis E.H., Suwa T. (2015). A novel impact of a novel weapon: Allelochemicals in *Alliaria petiolata* disrupt the legume-rhizobia mutualism. Biol. Invasions.

[19] Jensen J., Styrishave B., Gimsing A.L., Bruun Hansen H.C. (2010). The toxic effects of benzyl glucosinolate and its hydrolysis product, the biofumigant benzyl isothiocyanate, to *Folsomia fimetaria*. Environ. Toxicol. Chem..

[20] Hanschen F.S., Yim B., Winkelmann T., Smalla K., Schreiner M. (2015). Degradation of Biofumigant Isothiocyanates and Allyl Glucosinolate in Soil and Their Effects on the Microbial Community Composition. PLoS ONE.

[21] Miklavčič Višnjevec A., Tamayo Tenorio A., Steenkjær Hastrup A.C., Hansen N.M.L., Peeters K., Schwarzkopf M. (2021). Glucosinolates and Isothiocyantes in Processed Rapeseed Determined by HPLC-DAD-qTOF. Plants.

[22] Vieites-Outes C., López-Hernández J., Lage-Yusty M.A. (2016). Modification of glucosinolates in turnip greens (*Brassica rapa* subsp. rapa L.) subjected to culinary heat processes. CYTA J. Food.

[23] Possenti M., Baima S., Raffo A., Durazzo A., Giusti A.M., Natella F., Mérillon J.M., Ramawat K. (2016). Glucosinolates in Food. Glucosinolates. Reference Series in Phytochemistry.

[24] Pasini F., Verardo V., Cerretani L., Caboni M.F., D’Antuono L.F. (2011). Rocket salad (*Diplotaxis* and *Eruca* spp.) sensory analysis and relation with glucosinolate and phenolic content. J. Sci. Food Agric..

[25] Agerbirk N., Matthes A., Erthmann P.Ø., Ugolini L., Cinti S., Lazaridi E., Nuzillard J.M., Müller C., Bak S., Rollin P. (2018). Glucosinolate turnover in Brassicales species to an oxazolidin-2-one, formed via the 2-thione and without formation of thioamide. Phytochemistry.

[26] Pandit N., Singla R.K., Shrivastava B. (2012). Current updates on oxazolidinone and its significance. Int. J. Med. Chem..

[27] Bozdogan B., Appelbaum P.C. (2004). Oxazolidinones: Activity, mode of action, and mechanism of resistance. Int. J. Antimicrob. Agents.

[28] Foti C., Piperno A., Scala A., Giuffrè O. (2021). Oxazolidinone Antibiotics: Chemical, Biological and Analytical Aspects. Molecules.

[29] Li J., Shi Y., Xu Y., Yang L., Wang Z., Han H., Wang R. (2019). Metabolic profiles and pharmacokinetics of goitrin in rats through liquid chromatography combined with electrospray ionization–tandem mass spectrometry. Biomed. Chromatogr..

[30] Jeschke V., Zalucki J.M., Raguschke B., Gershenzon J., Heckel D.G., Zalucki M.P., Vassão D.G. (2021). So Much for Glucosinolates: A Generalist Does Survive and Develop on Brassicas, but at What Cost?. Plants.

[31] Coudert E., Gehant S., de Castro E., Pozzato M., Baratin D., Neto T., Sigrist C.J.A., Redaschi N., Bridge A. (2023). UniProt Consortium. Annotation of biologically relevant ligands in UniProtKB using ChEBI. Bioinformatics.

[32] Wall E.A., Majdalani N., Gottesman S. (2020). IgaA negatively regulates the Rcs Phosphorelay via contact with the RcsD Phosphotransfer Protein. PLoS Genet..

[33] Wang L., Xu C., Wang C., Wang Y. (2012). Characterization of a eukaryotic translation initiation factor 5A homolog from *Tamarix androssowii* involved in plant abiotic stress tolerance. BMC Plant Biol..

[34] Njenga R., Boele J., Öztürk Y., Koch H.G. (2023). Coping with stress: How bacteria fine-tune protein synthesis and protein transport. J. Biol. Chem..

[35] Henderson B., Allan E., Coates A.R. (2006). Stress wars: The direct role of host and bacterial molecular chaperones in bacterial infection. Infect. Immun..

[36] Mezzina M.P., Pettinari M.J. (2016). Phasins, Multifaceted Polyhydroxyalkanoate Granule-Associated Proteins. Appl. Environ. Microbiol..

[37] Brown B., Immethun C., Wilkins M., Saha R. (2022). Biotechnical applications of phasins: Small proteins with large potential. Renew. Sustain. Energy Rev..

[38] Lee H.J., Jung H.J., Kim B., Cho D.H., Kim S.H., Bhatia S.K., Gurav R., Kim Y.-G., Jung S.-W., Park H.J. (2023). Enhancement of polyhydroxybutyrate production by introduction of heterologous phasin combination in *Escherichia coli*. Int. J. Biol. Macromol..

[39] Paskevicius T., Farraj R.A., Michalak M., Agellon L.B. (2023). Calnexin, More Than Just a Molecular Chaperone. Cells.

[40] Arashida H., Odake H., Sugawara M., Noda R., Kakizaki K., Ohkubo S., Mitsui H., Sato S., Minamisawa K. (2022). Evolution of rhizobial symbiosis islands through insertion sequence-mediated deletion and duplication. ISME J..

[41] Solmi L., Rossi F.R., Romero F.M., Bach-Pages M., Preston G.M., Ruiz O.A., Gárriz A. (2023). Polyamine-mediated mechanisms contribute to oxidative stress tolerance in *Pseudomonas syringae*. Sci. Rep..

[42] Janczarek M. (2011). Environmental Signals and Regulatory Pathways That Influence Exopolysaccharide Production in *Rhizobia*. Int. J. Mol. Sci..

[43] Acosta-Jurado S., Fuentes-Romero F., Ruiz-Sainz J.-E., Janczarek M., Vinardell J.-M. (2021). Rhizobial Exopolysaccharides: Genetic Regulation of Their Synthesis and Relevance in Symbiosis with Legumes. Int. J. Mol. Sci..

[44] Chungopast S., Hirakawa H., Sato S., Handa Y., Saito K., Kawaguchi M., Tajima S., Nomura M. (2014). Transcriptomic profiles of nodule senescence in *Lotus japonicus* and *Mesorhizobium loti* symbiosis. Plant Biotechnol..

[45] Kovács Á.T. (2023). Plant–microbe interactions: Plant-exuded myo-inositol attracts specific bacterial taxa. Curr. Biol..

[46] Braun H.P., Binder S., Brennicke A., Eubel H., Fernie A.R., Finkemeier I., Klodmann J., König A.-C., Kühn K., Meyer E. (2014). The life of plant mitochondrial complex I. Mitochondrion.

[47] Forchhammer K., Selim K.A., Huergo L.F. (2022). New views on PII signaling: From nitrogen sensing to global metabolic control. Trends Microbiol..

[48] Flemetakis E., Efrose R.C., Desbrosses G., Dimou M., Delis C., Aivalakis G., Udvardi M.K., Katinakis P. (2004). Induction and spatial organization of polyamine biosynthesis during nodule development in *Lotus japonicus*. Mol. Plant Microbe Interact..

[49] Hernandez-Benitez E.M., Martínez-Romero E., Aguirre-Noyola J.L., Ledezma-Tejeida D. (2024). Putrescine acts as a signaling metabolite in the transition from nodulation to nitrogen fixation in *Rhizobium phaseoli*. bioRxiv.

[50] Lahiri K., Chattopadhyay S., Ghosh B. (2004). Correlation of endogenous free polyamine levels with root nodule senescence in different genotypes in *Vigna mungo* L. J. Plant Physiol..

[51] Wu X., Wang Y., Bian Y., Ren Y., Xu X., Zhou F., Ding H. (2022). A critical review on plant annexin: Structure, function, and mechanism. Plant Physiol. Biochem..

[52] Tatsukami Y., Ueda M. (2016). Rhizobial gibberellin negatively regulates host nodule number. Sci. Rep..

[53] Nett R.S., Bender K.S., Peters R.J. (2022). Production of the plant hormone gibberellin by rhizobia increases host legume nodule size. ISME J..

[54] Pieterse C.M.J., Van der Does D., Zamioudis C., Leon-Reyes A., Van Wees S.C.M. (2012). Hormonal Modulation of Plant Immunity. Annu. Rev. Cell Dev. Biol..

[55] Wei Z., Li J. (2020). Regulation of Brassinosteroid Homeostasis in Higher Plants. Front Plant Sci..

[56] Aglas L., Soh W.T., Kraiem A., Wenger M., Brandstetter H., Ferreira F. (2020). Ligand Binding of PR-10 Proteins with a Particular Focus on the Bet v 1 Allergen Family. Curr. Allergy Asthma Rep..

[57] Saxena I., Srikanth S., Chen Z. (2016). Cross Talk between H_2_O_2_ and Interacting Signal Molecules under Plant Stress Response. Front. Plant Sci..

[58] Wen B., Zhao X., Gong X., Zhao W., Sun M., Chen X., Li D., Li L., Xiao W. (2023). The NAC transcription factor MdNAC4 positively regulates nitrogen deficiency-induced leaf senescence by enhancing ABA biosynthesis in apple. Mol. Hortic..

[59] Eckardt N.A. (2001). Transcription factors dial 14-3-3 for nuclear shuttle. Plant Cell.

[60] Zheng Q., Xin J., Zhao C., Tian R. (2024). Role of methylglyoxal and glyoxalase in the regulation of plant response to heavy metal stress. Plant Cell Rep..

[61] Mansoor S., Ali Wani O., Lone J.K., Manhas S., Kour N., Alam P., Ahmad A., Ahmad P. (2022). Reactive Oxygen Species in Plants: From Source to Sink. Antioxidants.

[62] Shomali A., Das S., Arif N., Sarraf M., Zahra N., Yadav V., Aliniaeifard S., Chauhan D.K., Hasanuzzaman M. (2022). Diverse Diverse Physiological Roles of Flavonoids in Plant Environmental Stress Responses and Tolerance. Plants.

[63] Mihailović N., Bogdanović M., Dražić G., Filipović R., Radin D. (1994). Concentration-dependent influence of quercetin on nodulation process and the main characteristics of soybean inoculated with *Bradyrhizobium japonicum*. Plant Soil.

[64] Liu C.-W., Murray J.D. (2016). The Role of Flavonoids in Nodulation Host-Range Specificity: An Update. Plants.

[65] Perret X., Staehelin C., Broughton W.J. (2000). Molecular Basis of Symbiotic Promiscuity. Microbiol. Mol. Biol. Rev..

[66] Takeda N., Sato S., Asamizu E., Tabata S., Parniske M. (2009). Apoplastic plant subtilases support arbuscular mycorrhiza development in *Lotus japonicus*. Plant J..

[67] Pandey D.K., Kumar V., Chaudhary B. (2022). Concomitant Expression Evolution of Cell Wall Cytoskeletal Geneic Triad(s) Controls Floral Organ Shape and Fiber Emergence in Cotton (Gossypium). Front. Plant Sci..

[68] Krishnan K., Moens P.D.J. (2009). Structure and functions of profilins. Biophys Rev..

[69] Tatsukami Y., Nambu M., Morisaka H., Kuroda K., Ueda M. (2013). Disclosure of the differences of *Mesorhizobium loti* under the free-living and symbiotic conditions by comparative proteome analysis without bacteroid isolation. BMC Microbiol..

[70] Maslowska K.H., Makiela-Dzbenska K., Fijalkowska I.J. (2019). The SOS system: A complex and tightly regulated response to DNA damage. Environ. Mol. Mutagen..

[71] Aseev L.V., Boni I.V. (2011). Extraribosomal functions of bacterial ribosomal proteins. Mol. Biol..

[72] Winther K.S., Sorensen M.A., Svenningsen S.L. (2012). Polyamines are required for tRNA anticodon modification in *Escherichia coli*. J. Mol. Biol..

[73] Keller C., Chattopadhyay M., Tabor H. (2019). Absolute requirement for polyamines for growth of *Escherichia coli* mutants (mnmE/G) defective in modification of the wobble anticodon of transfer-RNA. FEMS Microbiol. Lett..

[74] Filloux A. (2022). Bacterial protein secretion systems: Game of types. Microbiology.

[75] Quistgaard E.M., Weininger U., Ural-Blimke Y., Modig K., Nordlund P., Akke M., Löw C. (2016). Molecular insights into substrate recognition and catalytic mechanism of the chaperone and FKBP peptidyl-prolyl isomerase SlyD. BMC Biol..

[76] Fujiyama-Nakamura S., Yoshikawa H., Homma K., Yamauchie Y., Isobe T., Takahashi N. (2009). Parvulin (Par14), a peptidyl-prolyl cis-trans isomerase, is a novel rRNA processing factor that evolved in the metazoan lineage. Mol. Cell. Proteom..

[77] Ünal C.M., Steinert M. (2014). Microbial Peptidyl-Prolyl cis/trans Isomerases (PPIases): Virulence Factors and Potential Alternative Drug Targets. MMBR.

[78] Castanié-Cornet M.P., Bruel N., Genevaux P. (2014). Chaperone networking facilitates protein targeting to the bacterial cytoplasmic membrane. Biochim. Biophys. Acta.

[79] Hartl F.U. (2017). Protein Misfolding Diseases. Annu. Rev. Biochem..

[80] Cerna-Vargas J.P., Sánchez-Romera B., Matilla M.A., Ortega Á., Krell T. (2023). Sensing preferences for prokaryotic solute binding protein families. Microb. Biotechnol..

[81] Matilla M.A., Ortega Á., Krell T. (2021). The role of solute binding proteins in signal transduction. Comput. Struct. Biotechnol. J..

[82] Albrecht R., Zeth K. (2011). Structural basis of outer membrane protein biogenesis in bacteria. J. Biol. Chem..

[83] Doyle M.T., Bernstein H.D. (2019). Bacterial outer membrane proteins assemble via asymmetric interactions with the BamA β-barrel. Nat. Commun..

[84] Sulatskaya A.I., Kosolapova A.O., Bobylev A.G., Belousov M.V., Antonets K.S., Sulatsky M.I., Kuznetsova I.M., Turoverov K., Stepanenko O.V., Nizhnikov A.A. (2021). β-Barrels and Amyloids: Structural Transitions, Biological Functions, and Pathogenesis. Int. J. Mol. Sci..

[85] Santos-Martin C., Wang G., Subedi P., Hor L., Totsika M., Paxman J.J., Heras B. (2021). Structural bioinformatic analysis of DsbA proteins and their pathogenicity associated substrates. Comput. Struct. Biotechnol. J..

[86] Chauviat A., Meyer T., Favre-Bonté S. (2023). Versatility of *Stenotrophomonas maltophilia:* Ecological roles of RND efflux pumps. Heliyon.

[87] Zeller T., Klug G. (2006). Thioredoxins in bacteria: Functions in oxidative stress response and regulation of thioredoxin genes. Naturwissenschaften.

[88] Grosdidier A., Zoete V., Michielin O. (2011). SwissDock, a protein-small molecule docking web service based on EADock DSS. Nucleic Acids Res..

[89] Fukamizo T., Kitaoku Y., Suginta W. (2019). Periplasmic solute-binding proteins: Structure classification and chitooligosaccharide recognition. Int. J. Biol. Macromol..

[90] Minguillón S., Matamoros M.A., Duanmu D., Becana M. (2022). Signaling by reactive molecules and antioxidants in legume nodules. New Phytol..

[91] Kazmierczak T., Yang L., Boncompagni E., Meilhoc E., Frugier F., Frendo P., Bruand C., Gruber V., Brouquisse R. (2020). Legume nodule senescence: A coordinated death mechanism between bacteria and plant cells. Adv. Bot. Res..

[92] Puppo A., Groten K., Bastian F., Carzaniga R., Soussi M., Lucas M.M., De Felipe M.R., Harrison J., Vanacker H., Foyer C.H. (2005). Legume nodule senescence: Roles for redox and hormone signalling in the orchestration of the natural aging process. New Phytol..

[93] Paço A., Brígido C., Alexandre A., Mateos P.F., Oliveira S. (2016). The Symbiotic Performance of Chickpea Rhizobia Can Be Improved by Additional Copies of the clpB Chaperone Gene. PLoS ONE.

[94] Ogawa J., Long S.R. (1995). The *Rhizobium meliloti* groELc locus is required for regulation of early nod genes by the transcription activator NodD. Genes Dev..

[95] Van de Velde W., Guerra J.C., De Keyser A., De Rycke R., Rombauts S., Maunoury N., Mergaert P., Kondorosi E., Holsters M., Sofie Goormachtig S. (2006). Aging in legume symbiosis. A molecular view on nodule senescence in *Medicago truncatula*. Plant Physiol..

[96] Berrabah F., Benaceur F., Yin C., Xin D., Magne K., Garmier M., Gruber V., Ratet P. (2024). Defense and senescence interplay in legume nodules. Plant Commun..

[97] Berrabah F., Bernal G., Elhosseyn A.S., El Kassis C., L’Horset R., Benaceur F., Wen J., Mysore K.S., Garmier M., Gourion B. (2023). Insight into the control of nodule immunity and senescence during *Medicago truncatula* symbiosis. Plant Physiol..

[98] Guan P., Zhou J., Girel S., Zhu X., Schwab M., Zhang K., Wang-Müller Q., Bigler L., Nick P. (2021). Anti—Microtubule activity of the traditional Chinese medicine herb Northern Ban Lan (*Isatis tinctoria*) leads to glucobrassicin. J. Integr. Plant Biol..

[99] Schink S., Ammar C., Chang Y.F., Zimmer R., Basan M. (2022). Analysis of proteome adaptation reveals a key role of the bacterial envelope in starvation survival. Mol. Syst. Biol..

[100] Bergé M., Pezzatti J., González-Ruiz V., Degeorges L., Mottet-Osman G., Rudaz S., Viollier P.H. (2020). Bacterial cell cycle control by citrate synthase independent of enzymatic activity. eLife.

[101] Li X.-S., Xue J.-Z., Qi Y., Muhammad I., Wang H., Li X.-Y., Luo Y.-J., Zhu D.-M., Gao Y.-H., Kong L.-C. (2023). Citric Acid Confers Broad Antibiotic Tolerance through Alteration of Bacterial Metabolism and Oxidative Stress. Int. J. Mol. Sci..

[102] Green L.K., La Flamme A.C., Ackerley D.F. (2014). *Pseudomonas aeruginosa* MdaB and WrbA are water-soluble two-electron quinone oxidoreductases with the potential to defend against oxidative stress. J. Microbiol..

[103] Kumari V., Dyba M.A., Holland R.J., Liang Y.H., Singh S.V., Ji X. (2016). Irreversible Inhibition of Glutathione S-Transferase by Phenethyl Isothiocyanate (PEITC), a Dietary Cancer Chemopreventive Phytochemical. PLoS ONE.

[104] Simms E.J., Taylor D.L. (2002). Partner Choice in Nitrogen-Fixation Mutualisms of Legumes and Rhizobia. ICB.

[105] Newair E.F., Mohamed I.M.A., Garcia F., Al-Anazi A. (2023). Caftaric acid oxidation in the presence of cell signaling regulator glutathione: Electrochemical and chromatographic analyses. Microchem. J..

[106] Huang Y.-H., You W.-C., Chen Y.-J., Ciou J.-Y., Hsieh L.-S. (2023). Insight into the Substrate Specificity of *Lactobacillus paracasei* Aspartate Ammonia-Lyase. Fermentation.

[107] Alvarez L.A., Kovačič L., Rodríguez J., Gosemann J.H., Kubica M., Pircalabioru G.G., Friedmacher F., Cean A., Ghişe A., Sărăndan M.B. (2016). NADPH oxidase-derived H_2_O_2_ subverts pathogen signaling by oxidative phosphotyrosine conversion to PB-DOPA. Proc. Natl. Acad. Sci. USA.

[108] Khan M., Kar S., Wang J., Leszczynski J. (2019). Theoretical study of formate, tartrate, tartronate, and glycolate production from 6-carbon trioxylate intermediate in the citric acid cycle. J. Mol. Model..

[109] Ford C.M., Sweetman C., Fry S.C. (2024). Ascorbate degradation: Pathways, products, and possibilities. J. Exp. Bot..

[110] Xu L., Zhang Y., Wang L., Chen W., Wei G. (2014). Diversity of endophytic bacteria associated with nodules of two indigenous legumes at different altitudes of the Qilian Mountains in China. Syst. Appl. Microbiol..

[111] Meng X., Yan D., Long X., Wang C., Liu Z., Rengel Z. (2014). Colonization by endophytic *Ochrobactrum anthropi* Mn1 promotes growth of Jerusalem artichoke. Microb Biotechnol..

[112] Msaddak A., Mars M., Quiñones M.A., Lucas M.M., Pueyo J.J. (2023). Lupin, a Unique Legume That Is Nodulated by Multiple Microsymbionts: The Role of Horizontal Gene Transfer. Int. J. Mol. Sci..

[113] Hossain M.S., DeLaune P.B., Gentry T.J. (2023). Microbiome analysis revealed distinct microbial communities occupying different sized nodules in field-grown peanut. Front. Microbiol..

[114] Hansen B.L., Pessotti R.C., Fischer M., Collins A., El-Hifnawi L., Liu M.D., Traxler M.F. (2020). Cooperation, Competition, and Specialized Metabolism in a Simplified Root Nodule Microbiome. mBio.

[115] Hanschen F.S., Winkelmann T. (2020). Biofumigation for Fighting Replant Disease- A Review. Agronomy.

[116] Edwards S., Ploeg A. (2014). Evaluation of 31 potential biofumigant brassicaceous plants as hosts for three meloiodogyne species. J. Nematol..

[117] Shanmugam S., Hefner M., Pelck J.S., Labouriau R., Kristensen H.L. (2022). Complementary resource use in intercropped faba bean and cabbage by increased root growth and nitrogen use in organic production. Soil Use Manag..

[118] Génard T., Etienne P., Diquélou S., Yvin J.-C., Revellin C., Laîné P. (2017). Rapeseed-legume intercrops: Plant growth and nitrogen balance in early stages of growth and development. Heliyon.

[119] Felker P., Bunch R., Leung A.M. (2016). Concentrations of thiocyanate and goitrin in human plasma, their precursor concentrations in brassica vegetables, and associated potential risk for hypothyroidism. Nutr. Rev..

[120] Alexander N.M., Zenker N., Medeiros-Neto G., Gaitan E. (1986). Inhibition of Thyroid Peroxidase (TPO) and Lactoperoxidase (LPO) by Goitrin and Ricinine. Frontiers in Thyroidology.

[121] Narbad A., Rossiter J.T. (2018). Gut Glucosinolate Metabolism and Isothiocyanate Production. Mol. Nutr. Food Res..

[122] Schütz V., Frindte K., Cui J., Zhang P., Hacquard S., Schulze-Lefert P., Knief C., Schulz M., Dörmann P. (2021). Differential Impact of Plant Secondary Metabolites on the Soil Microbiota. Front. Microbiol..

[123] Pinevich A.V., Andronov E.E., Pershina E.V., Pinevich A.A., Dmitrieva H.Y. (2018). Testing culture purity in prokaryotes: Criteria and challenges. Antonie Van Leeuwenhoek.

[124] Wessel D., Flügge U.I. (1984). A method for the quantitative recovery of protein in dilute solution in the presence of detergents and lipids. Anal. Biochem..

[125] Beck S., Michalski A., Raether O., Lubeck M., Kaspar S., Goedecke N., Baessmann C., Hornburg D., Meier F., Paront I. (2015). The Impact II, a Very High-Resolution Quadrupole Time-of-Flight Instrument (QTOF) for Deep Shotgun Proteomics. Mol. Cell. Proteom..

[126] Tyanova S., Temu T., Cox J. (2016). The MaxQuant computational platform for mass spectrometry-based shotgun proteomics. Nat. Protoc..

[127] Cox J., Neuhauser N., Michalski A., Scheltema R.A., Olsen J.V., Mann M. (2011). Andromeda: A Peptide Search Engine Integrated into the MaxQuant Environment. Proteome Res..

[128] Kaneko T., Nakamura Y., Sato S., Asamizu E., Kato T., Sasamoto S., Watanabe A., Idesawa K., Ishikawa A., Kawashima K. (2000). Complete genome structure of the nitrogen-fixing symbiotic bacterium *Mesorhizobium loti*. DNA Res..

[129] Dam S., Dyrlund T.F., Ussatjuk A., Jochimsen B., Nielsen K., Goffard N., Ventosa M., Lorentzen A., Gupta V., Andersen S.U. (2014). Proteome reference maps of the *Lotus japonicus* nodule and root. Proteomics.

[130] Jumper J., Evans R., Pritzel A., Green T., Figurnov M., Ronneberger O., Tunyasuvunakool K., Bates R., Žídek A., Potapenko A. (2021). Highly accurate protein structure prediction with AlphaFold. Nature.

[131] Varadi M., Anyango S., Deshpande M., Nair S., Natassia C., Yordanova G., Yuan D., Stroe O., Wood G., Laydon A. (2022). AlphaFold Protein Structure Database: Massively expanding the structural coverage of protein-sequence space with high-accuracy models. Nucleic Acids Res..

[132] Pieper U., Eswar N., Davis F., Braberg H., Madhusudhan M.S., Rossi A., Marti-Renom M., Karchin R., Webb B.M., Eramian D. (2006). MODBASE: A database of annotated comparative protein structure models and associated resources. Nucleic Acids Res..

[133] Pettersen E., Goddard T., Huang C., Couch G.S., Greenblatt D.M., Meng E.C., Ferrin T.E. (2004). UCSF Chimera-a visualization system for exploratory research and analysis. J. Comput. Chem..

