# Supplementary material for: Cyclic Isothiocyanate Goitrin Impairs Lotus japonicus Nodulation, Affects the Proteomes of Nodules and Free Mesorhizobium loti, and Induces the Formation of Caffeic Acid Derivatives in Bacterial Cultures
Source: Plants (Basel). 2024 Oct 16;13(20):2897. doi: 10.3390/plants13202897 (PMC11511026; doi:10.3390/plants13202897)
Supplement: Supplementary file 1 [file plants-13-02897-s001.zip › plants-3242909-supplementary.pdf]

Supplementary Materials:

Cyclic Isothiocyanate Goitrin Impairs *Lotus japonicus* Nodulation, Affects the Proteomes of Nodules and free *Mesorhizobium loti* and Induces the Formation of Caffeic acid Derivatives in the Bacterial Cultures

Seungwoo Jeong [1], Vadim Schütz [1,†], Fatih Demir [2], Matthias Preusche [1,3], Pitter Huesgen [4], Laurent Bigler [5], Filip Kovacic [6,‡], Katharina Gutbrod [1], Peter Dörmann [1] and Margot Schulz [1,*]

[1] IMBIO Institute of Molecular Biotechnology, University of Bonn, 53115 Bonn, Germany; s6sejeon@uni-bonn.de (S.J.); v_schuetz@snu.ac.kr (V.S.); m.preusche@hs-osnabrueck.de (M.P.); gutbrod@uni-bonn.de (K.G.); doermann@uni-bonn.de (P.D.)

[2] Department of Biomedicine, Aarhus University, 8000 Aarhus, Denmark; fatih.demir@bio-med.au.dk

[3] Faculty of Agricultural Sciences and Landscape Architecture, University of Applied Sciences Osnabrueck, 49090 Osnabrueck, Germany

[4] Faculty of Biology, University of Freiburg, 79104 Freiburg, Germany; pitter.huesgen@biologie.uni-freiburg.de

[5] Department of Chemistry, University of Zurich, CH-8057 Zurich, Switzerland; laurent.bigler@chem.uzh.ch

[6] Institute of Molecular Enzyme Technology, Heinrich-Heine-University of Düsseldorf, Forschungszentrum Jülich, 52428 Jülich, Germany; fkovacic@mgh.harvard.edu

* Correspondence: ulp509@uni-bonn.de; Tel.: +49-(0)-228-735121

† Current address: Research Center of Plant Plasticity, Seoul National University, Seoul 08826, Republic of Korea.

‡ Current address: Massachusetts General Hospital & Harvard Medical School, Massachusetts, Boston, MA 02114, USA.

Tables Large Nodule Proteome

Tables S1-17, A and B series. Goitrin treated large nodules versus control nodules. Fold change (FC) of down and up regulated host and microsymbiont proteins. The $-\log_{10}$ q values > 1.5 are significant, and FCs of changed protein abundancies (specified range: < 0.3; > -0.3 log2 FC, with exceptions) are listed. IDs of goitrin docking candidates are in bold, ΔG: estimated -ΔG (kcal/mol). S-Tables A series: microsymbiont; B series: host; Table S16: flavonoids.

Table S1A: DNA, Transcription and Purine Metabolism, Microsymbiont

ID	**Protein function, Symbiotic *Mesorhizobium loti***	**FC**	**$-\log_{10}$ q values**	**ΔG**
	**DNA, transcription and purin metabolism**			
Q988T3	Histidine kinase, phosphorelay sensor kinase activity (complex variation of a two-component regulatory system), Signal transduction, regulation of DNA transcription	1.76	0.44	
Q987Y5	Two-component, response regulator, sequence-specific DNA binding, phosphorelay signal transduction system	-0.51	0.64	
Q983B3	Transcriptional repressor NrdR, negative regulation of DNA-templated transcription	1.26	0.82	
Q98FB9	Protein possesses domain found at the N-terminus of the Polyhydroxyalkanoate (PHA) synthesis regulators regulation of DNA-templated transcription [GO:0006355]	1.05	2.52	
Q98M65	Glycerol-3-phosphate regulon repressor, transcription regulation	0.84	0.89	
Q98FN0	RNA polymerase sigma factor, transcription regulation	0.59	0.77	

Q98BH0	Beta sliding clamp, DNA polymerase III complex, DNA strand elongation involved in DNA replication	1.47	0.49	
Q982Z7	Integration host factor subunit alpha, structural constituent of chromatin, regulation of DNA-templated transcription	1.33	0.64	
Q985Y6	Exodeoxyribonuclease 7 small subunit, degrades single-stranded DNA into large acid-insoluble oligonucleotides	1.30	0.91	
Q98M41	Single-stranded DNA-binding protein, DNA repair, DNA recombination, DNA replication	1.23	1.20	
Q982V3	DNA-binding protein HRm, DNA-binding, DNA condensation	0.30	1.16	
Q98FX1	Protein Q98FX1 possesses IAA-Ala conjugate hydrolase activity, releases free IAA	0.92	0.58	
Q98M59	Probable transcription regulator	0.89	2.15	
Q98NN0	Phosphoribosylformylglycinamidine synthase subunit PurQ, purine biosynthetic pathway.	-0.39	1.46	

Table S1B: DNA and Transcription, Host

ID	**Host Protein Function, *Lotus japonicus* Nodule**	**FC**	**–log$_{10}$ q values**	**ΔG**
	**DNA and Transcription**			
I3RZH4	Variant histone H3 which replaces conventional H3 in a wide range of nucleosomes in active genes.	2.32	1.21	
I3SLS9	Histone H2B, Core component of nucleosome	1.82	0.55	
I3SZ07	Histone H2A, the nucleosome is a histone octamer containing two molecules each of H2A, H2B, H3 and H4	1.40	0.55	

Table S2A: Ribosome and Translation, Microsymbiont

ID	**Protein function, Symbiotic *Mesorhizobium loti***	**FC**	**–log$_{10}$ q values**	**ΔG**
	**Ribosome and Translation**			
Q98N70	Large ribosomal subunit protein uL11, Forms part of the ribosomal stalk which helps the ribosome interact with GTP-bound translation factors. GTP: binding of a new amino bound tRNA to the A-site of the ribosome.	1.59	1.41	
Q98FN3	Membrane protein, ribosome binding	1.19	0.83	
Q98FZ7	Large ribosomal subunit protein bL28	1.29	0.81	
Q98N51	Small ribosomal subunit protein uS3	-0.31	0.38	
Q98N43	Small ribosomal subunit protein uS8, one of the primary rRNA binding proteins	-0.46	2.49	
Q98N54	Large ribosomal subunit protein uL2	-0.47	1.42	
Q98N50	Large ribosomal subunit protein uL16	1.19	0.51	
P58168	Large ribosomal subunit protein bL19	1.05	0.34	
Q98BI8	Translation initiation factor IF-2, essential component for the initiation of protein synthesis	1.02	0.33	
Q98N39	uL30, probably no essential ribosomal protein	0.97	0.43	
Q98N47	Large ribosomal subunit protein uL14, rRNA binding	0.92	0.44	
Q98N32	Large ribosomal subunit protein bL17	0.91	0.42	
Q98N44	Small ribosomal subunit protein uS14	0.85	0.41	
Q98BI3	Polyribonucleotide nucleotidyl-transferase, involved in mRNA degradation.	0.83	0.38	
Q98GS6	Ribosome hibernation promoting factor, required for dimerization of active 70S ribosomes into 100S ribosomes in stationary phase	0.79	0.60	
Q98N56	Large ribosomal subunit protein uL4	-0.28	0.94	
Q98N59	Elongation factor G, ribosome disassembly	-0.49	0.87	
Q98NP5	Small ribosomal subunit protein uS4	-0.50	1.35	
Q981F7	Elongation factor Tu, GTP-dependent binding of aminoacyl-tRNA to the A-site of ribosomes	-0.59	2.63	
Q98CP5	Translation initiation factor IF-3	1.31	0.94	
Q98NB6	Ribonuclease E, central role in RNA processing and decay, maturation of 5S and 16S rRNAs and the majority of tRNAs, involved in the degradation of most mRNAs.	-0.39	1.35	
Q98HX2	Probable chaperone, tyrosine-tRNA ligase activity	1.00	1.06	

Q98KS5	Proline-tRNA ligase	-0.43	1.33

Table S2B: Ribosome and Translation, Host

ID	Host Protein Function, *Lotus japonicus* Nodule	FC	–log10 q values	ΔG
	**Ribosome and Translation**			
I3SR66	Large ribosomal subunit protein uL11; significant role during initiation, elongation, and termination of protein synthesis	2.60	1.13	
I3SR06	40S ribosomal protein S17, ribonucleoprotein complex	2.06	1.21	
I3SGN7	Ribosomal protein L3, RNA binding	1.80	1.12	
I3SX58	Large ribosomal subunit protein uL15/eL18 domain-containing protein, mRNA binding	1.76	1.04	
I3SHC8	60S ribosomal protein L7a, maturation of LSU-rRNA	1.67	0.66	
I3S5S4	40S ribosomal protein S27, RNA binding	1.61	1.27	
I3T5Q5	Large ribosomal subunit protein eL14 domain-containing protein, RNA binding	1.61	1.00	
I3SZQ1	KOW domain-containing protein, structural constituent of ribosome, KOW motif: in many ribosomal proteins and bacterial transcription antitermination proteins NusG	1.59	1.14	
I3S397	40S ribosomal protein S6, structural constituent of ribosome	1.49	0.59	
I3T869	Plectin/eS10 N-terminal domain-containing protein, cytosolic small ribosomal subunit	1.46	0.81	
I3S7K3	Large ribosomal subunit protein uL15/eL18 domain-containing, mRNA binding	1.47	1.23	
I3SBE6	Ribosomal protein L19	1.41	0.82	
I3S3B3	Ribosomal protein L3, RNA binding	1.31	1.27	
I3T8K2	Ribosomal protein L22e	1.13	0.53	
I3T8Q2	Small ribosomal subunit protein uS15	1.09	1.27	
I3SWF1	60S ribosomal protein L21	1.00	0.59	
I3SGZ8	40S ribosomal protein S24	1.00	0.41	
I3T8M4	Ribosomal protein L5 C-terminal domain	0.98	0.41	
I3T7R3	Ribosomal protein L35A	0.97	0.71	
I3SWB6	60S ribosomal protein L18a	0.90	0.35	
I3SIQ8	40S ribosomal protein S8	0.93	0.39	
I3S9R2	Ribosomal protein L30 ferredoxin-like fold domain-containing	0.83	1.10	
I3SG74	Small ribosomal subunit protein uS7, mRNA, rRNA binding	0.76	0.57	
I3SPM6	Eukaryotic translation initiation factor 5A	0.53	2.23	
I3SF98	PUA domain-containing protein, formation of translation preinitiation complex	1.01	0.66	
I3SEW9	Small ribosomal subunit protein uS17 N-terminal domain-containing protein	1.29	0.98	
I3SRW3	60S ribosomal protein L36	1.27	0.60	
I3S2A7	Ribosomal protein S11	1.26	0.48	
I3SAF6	Large ribosomal subunit protein uL6, rRNA binding	1.24	0.53	
I3SJC9	Small ribosomal subunit protein uS10 rRNA binding	1.23	0.80	
I3S6L4	Large ribosomal subunit protein uL6, rRNA binding	1.22	0.42	

Table S3A: Chaperone, Microsymbiont

ID	Protein function, Symbiotic *Mesorhizobium loti*	FC	–log10 q values	ΔG
	**Chaperone**			
Q988T6	Probably phasin related, chaperone activity	2.23	2.24	
Q98MA2	Phasin family protein, chaperone function	2.81	1.72	
Q988Y4	Phasin related (Q988Y4-1)	1.45	2.22	
Q98IH9	Chaperonin GroEL 2	4.18	2.35	
Q98II0	Co-chaperonin GroES 2	5.47	2.18	
Q98GQ5	Protein GrpE	0.58	2.61	
Q98IV5	Chaperonin GroEL 1, with its co-chaperonin GroES, plays an essential role in assisting protein folding.	0.06	0.87	
Q981K0	Co-chaperonin GroES 5, acts with the chaperonin GroEL	0.20	0.66	
Q981J9	Chaperonin GroEL 5, acts with its co-chaperonin GroES	0.38	0.41	
Q983S4	Chaperonin GroEL 4, acts with its co-chaperonin GroES	0.23	0.58	
**Q98AX9**	Chaperonin GroEL 3, protein folding	-0.09	0.32	-6.71

**Q98JB6**	Chaperone protein HtpG, stress protection	1.91	1.69	
Q98G96	Chaperone protein ClpB, acts in cooperation with DnaK, DnaJ and GrpE	1.80	0.93	
Q98IV4	Co-chaperonin GroES 1, GroES binds to the apical surface of the GroEL ring	0.72	0.43	
Q98DD1	Chaperone protein DnaK,	0.79	0.16	
Q983S3	Co-chaperonin GroES 4	0.39	0.52	
Q98LE8	peptidyl-prolyl cis-trans isomerase, involved in protein export. Acts as a chaperone	0.80	0.31	
Q98M53	peptidyl-prolyl cis-trans isomerase, acts as chaperone	-0.96	2.42	
Q985Z8	Heat shock protein DnaJ, chaperone	0.92	0.65	

Table S3B: Chaperone, Host

ID	Host Protein Function, *Lotus japonicus* Nodule	FC	$-\log_{10}$ q values	ΔG
	**Chaperone**			
I3SFP8	Co-chaperone protein p23, Hsp90 protein binding, response to stress	1.29	0.69	
I3SW99	10 kDa chaperonin, chaperone cofactor-dependent protein refolding	0.93	0.78	
I3SKH6	ATP-dependent protein folding chaperone	1.19	1.02	
V9VGV1	Hsp70 protein binding, chaperone cofactor-dependent protein refolding	0.88	0.60	
I3SD49	Calnexin homolog, ER membrane, chaperone, signaling	1.97	2.16	

Table S4A: Transporter, Microsymbiont

ID	Protein function, Symbiotic *Mesorhizobium loti*	FC	$-\log_{10}$ q values	ΔG
	**Transporter**			
Q98MC3	Outer membrane protein assembly factor BamA, Bam A family	0.77	2.51	
Q98D26	Branched-chain amino acid ABC transporter, periplasmic amino acid-binding protein, leucine-binding protein family	0.53	2.61	
Q98MN8	Probable ABC transporter substrate-binding protein, inner membrane, BMP family ABC transporter substrate-binding protein	0.60	2.33	
**Q98MK0**	ABC transporter, amino acid binding protein, bacterial solute-binding protein 3 family	0.95	2.62	-6.65
Q98IL8	type of VI secretion system-associated lipoprotein, outer membrane	0.79	0.97	
Q987M5	Putrescine transport, periplasmic space, bacterial solute-binding protein PotD/PotF family	1.87	1.55	
Q98M72	Periplasmic binding protein-like II, transporter	2.37	1.50	
**Q98GF4**	Periplasmic binding protein component of ATP transporter, phosphate/phosphite/phosphonate binding protein family	2.83	1.00	-6.57
Q982N3	ABC transporter, carbohydrate binding protein, bacterial solute-binding protein 2 family	1.03	1.35	
**Q985D9**	ABC transporter, probably amino acid binding protein, leucine-binding protein family	0.39	2.35	-6.56
Q987A7	Amino acid ABC transporter protein, solute-binding component, solute-binding protein family 3 domain	1.68	2.23	
**Q98CD0**	Amino acid ABC transporter, bacterial solute-binding protein 3 family	1.54	2.09	-7.00
Q98DM1	ATP transporter, membrane	-1.03	0.91	
Q98DQ2	Manganese ion transmembrane transporter activity, Rubrerythrin family protein, oxidative stress defense	1.50	1.68	
Q98H21	ATP-binding cassette (ABC) transporter complex(sulfate)	1.49	1.21	
Q98LD6	Protein-export membrane protein SecF, inner membrane, SecD/SecF family	1.47	1.00	
Q983Q2	Iron (III) ABC transporter, periplasmic-binding protein	1.20	2.35	
Q985H2	ABC transporter, (polyamine) binding protein	1.20	2.11	
Q98D21	Amino acid ABC transporter, periplasmic binding protein, bacterial solute-binding protein 3 family	1.15	0.52	

**Q98EE6**	Amino acid ABC transporter, periplasmic amino acid-binding protein	0.46	2.53	-7.11
Q98IG6	Outer membrane protein	2.21	2.02	
Q98HU7	Acid phosphatase, outer membrane-bounded periplasmic space	0.85	2.30	
Q98GC1	ABC transporter oligopeptide-binding protein, bacterial solute-binding protein 5 family	1.01	1.35	
Q983A7	ABC transporter, periplasmic substrate-binding protein	1.0	2.37	
**Q98H18**	ABC transporter substrate/thiamine-binding protein, linked to iron transport	0.87	1.85	-7.35
Q988Z1	Putrescine-binding periplasmic protein, bacterial solute-binding protein, PotD/PotF family	1.87	1.55	
Q98L42	Dipeptide-binding protein of ABC transporter, bacterial solute-binding protein 5 family	1.73	2.01	
Q986J3	ABC-type glycine betaine transport system substrate-binding	1.18	2.08	
Q98JX0	Spermidine/putrescine ABC transporter substrate-binding protein	1.15	1.62	
Q98HG0	Putrescine-binding periplasmic protein, polyamine transport, bacterial solute-binding protein PotD/PotF family	0.24	0.96	
Q98FL2	Phosphate-binding protein PstS, phosphate ion transmembrane transport, PstS family, enhanced under phosphate starvation	0.72	2.16	
Q98GS3	Lipopolysaccharide transport, cell outer membrane	1.44	1.33	
Q98JY5	Binding protein component of sugar ABC transporter, bacterial solute-binding protein 2 family	0.98	2.51	
Q98JB0	Spermidine/putrescine binding protein of ABC transporter	0.92	2.37	
**Q98LA8**	Outer membrane protein, efflux transmembrane transporter activity, NodT candidate, outer membrane factor (OMF) (TC 1.B.17) family	0.34	2.28	-6.51
Q98KI1	Molybdenum transport protein ModA, outer membrane-bounded periplasmic space, bacterial solute-binding protein ModA family	1.29	1.33	
Q98GK5	RND efflux membrane fusion protein, transmembrane transporter, response to antibiotics, membrane fusion protein (MFP) (TC 8.A.1) family.	1.28	0.68	
Q98K26	Sulfate binding protein of ABC transporter, periplasmic space	0.60	0.23	

Table S4B: Transporter, Host

ID	Host Protein Function, *Lotus japonicus* Nodule	FC	$-\log_{10}$ q values	ΔG
	**Transporter**			
I3SJ57	Guanosine nucleotide diphosphate dissociation inhibitor, protein transport, vesicle-mediated transport, small GTPase-mediated signal transduction, Rab GDP-dissociation inhibitor activity, cell dynamics and bacterial infection	1.80	1.35	
I3T6D5	ADP/ATP translocase, ATP:ADP antiporter activity, mitochondrial ATP transmembrane transport	0.35	2.26	
I3T9V3	Nuclear pore central transport channel, RNA, protein transport	1.26	0.66	

Table S5A: Carbohydrates, Microsymbiont

ID	Protein function, Symbiotic *Mesorhizobium loti*	FC	$-\log_{10}$ q values	ΔG
	**Carbohydrate metabolism**			
Q98C71	Glucosyltransferase, succinoglycan biosynthesis, anionic exopolysaccharide	2.34	2.52	
Q98MZ3	Enolase, glycolysis, 2-Phosphoglycerat - phosphoenolpyruvat	1.50	0.42	
Q984S4	Assembly protein of lipopolysaccharide (LPS) at the surface of the outer membrane	1.54	1.89	
Q98GS3	Lipopolysaccharide transport, cell outer membrane	1.44	1.33	
Q984T1	Endolytic murein transglycosylase, peptidoglycan biosynthetic process, cell wall organization	1.34	1.02	
Q98C78	Endo-1,3-1,4-beta-glycanase ExoK	1.87	2.18	
Q983H6	Cyclic beta 1-2 glucan synthetase, membrane	1.02	0.81	
Q98HX4	Probable glycosyl transferase, UDP-sulfoquinovose:DAG sulfoquinovosyltransferase activity, sulfolipid biosynthetic process	0.92	0.94	
Q98MY4	Dihydrolipoyl dehydrogenase, maintenance of glycolysis and TCA	0.61	0.37	
Q986N8	Fructose-bisphosphate aldolase activity, glycolytic process, reductive pentose-phosphate cycle	0.53	2.01	

Q98ME7	Triosephosphate isomerase, glyconeogenese	0.27	0.86	
Q98FJ0	Fructose-bisphosphate aldolase	-0.26	2.14	
Q98ED5	Dihydrolipoyl dehydrogenase, maintenance of glycolysis and TCA	-0.51	2.05	
Q986N5	Ribose-5-phosphate isomerase, carbohydrate metabolic process	1.62	1.69	
Q98CW1	Glucuronate isomerase, transformation to D-fructuronate inositol catabolic process,<br>Inositol as a C-source, plant-microbe interaction	1.29	2.29	

Table S5B: Carbohydrates, Host

**ID**	**Host Protein function, *Lotus japonicus* nodule**	**FC**	**–log$_{10}$ q values**	**ΔG**
	**Carbohydrate metabolism**			
B2CP01	Starch synthase	2.07	1.12	
**I3S413**	Alpha-L-fucosidase, hydrolysis of fucose-containing glycolipids and glycoproteins	-0.54	1.98	-6.57
I3SY05	Pectin acetylesterase, cell wall organization	1.61	1.96	
Q684K1	Alkaline/neutral invertase, decomposes sucrose into fructose and glucose	1.50	1.20	
I3SZG4	Triosephosphate isomerase, gluconeogenesis	0.86	0.50	
I3SW64	Glyceraldehyde 3-phosphate dehydrogenase, glycolytic process	0.45	2.08	
I3S9T1	Oxireductase, Glyceraldehyde 3-phosphate dehydrogenase NAD(P) binding domain-containing protein	0.31	1.56	
I3SDW4	Fructose-bisphosphate aldolase, glycolytic process	0.38	1.86	
I3SUT5	Triosephosphate isomerase, glycolytic process	0.31	2.07	
I3T0F4	Fructose-bisphosphate aldolase, glycolytic process	0.27	1.20	
I3SYQ7	Glyceraldehyde-3-phosphate dehydrogenase, glycolytic process	0.25	2.22	
I3SY55	Phosphoglycerate kinase, glycolytic process, gluconeogenesis	-0.49	1.51	

Table S6A: TCA, Microsymbiont

**ID**	**Protein function, Symbiotic *Mesorhizobium loti***	**FC**	**–log$_{10}$ q values**	**ΔG**
	**TCA**			
Q98MC9	Citrate synthase, moonlighting	1.17	0.30	
Q98FC3	Citrate synthase, moonlighting	0.99	0.64	
Q98EA3	Aconitate hydratase, citrate metabolic process	-0.76	2.66	
Q98MY9	Production of acetyl-CoA for TCA cycle, pyruvate dehydrogenase E1 component subunit alpha, part of pyruvate dehydrogenase complex which catalyzes the overall conversion of pyruvate to acetyl-CoA and CO_2	-0.55	1.55	
Q98E51	3-isopropylmalate dehydratase small subunit	1.35	0.87	
**Q98E57**	3-isopropylmalate dehydrogenase	0.96	0.38	

Table S6B: TCA, Host

**ID**	**Host Protein function, *Lotus japonicus* nodule**	**FC**	**–log$_{10}$ q values**	**ΔG**
	**TCA**			
I3S630	Dihydrolipoyllysine-residue succinyltransferase, lysine degradation, TCA	1.33	0.69	
I3T5L9	Isocitrate dehydrogenase, isopropylmalate dehydrogenase-like domain-containing protein	1.66	1.28	
I3SMU1	Succinate-CoA ligase	1.47	0.66	
I3SPT9	Isopropylmalate dehydrogenase-like domain-containing protein, isocitrate dehydrogenase	1.26	1.05	
I3T024	Malate dehydrogenase	0.47	2.32	
Q8H945	phosphoenolpyruvate carboxylase, formation of oxaloacetate for the tricarboxylic acid cycle	2.08	0.96	
I3SAF2	Aconitase A/isopropylmalate dehydratase small subunit swivel	1.22	1.54	
I3SFE2	Lactate/malate dehydrogenase C-terminal domain-containing	1.20	1.01	
D0VY45	2-isopropylmalate synthase	0.49	0.40	
I3SFA8	malate dehydrogenase	0.57	0.38	

Table S7A: Energy, Microsymbiont

ID	Protein function, Symbiotic *Mesorhizobium loti*	FC	–log$_{10}$ q values	ΔG
	**Energy, Respiratory chain and ATP synthesis**			
Q98NC7	Involved in respiratory chain complex I assembly, NADH dehydrogenase [ubiquinone] 1 alpha subcomplex subunit, response to oxidative stress	0.40	1.54	
Q98EV9	ATP synthase epsilon chain, inner membrane	-0.50	0.44	
Q98EV6	ATP synthase subunit alpha	-0.54	2.28	
Q987Z1	Ubiquinol oxidase polypeptide II	-0.37	1.67	
Q985X2	Cytochrome c oxidase subunit 2, respiratory chain	-0.98	2.51	
Q98HU5	Cytochrome b, Component of the ubiquinol-cytochrome c reductase complex, respiratory chain	-0.84	1.35	
Q98CD7	NADH dehydrogenase (Ubiquinone), respiratory chain	-0.37	2.56	
Q986D3	ATP synthase subunit c, plasma membrane, lipid binding	-1.81	1.25	
Q98EV8	ATP synthase subunit beta, proton-transporting ATP synthase complex,	-0.54	2.30	
Q98EV7	ATP synthase gamma chain, ATP synthesis	-0.95	2.18	

Table S7B: Energy, Host

ID	Host Protein function*, Lotus japonicus* nodule	FC	–log$_{10}$ q values	ΔG
	**Energy, Respiratory Chain, ATP synthesis**			
I3SSB5	NADH dehydrogenase [ubiquinone] 1 alpha subcomplex subunit 13, respiratory chain	1.51	1.04	
I3SGC8	NADH dehydrogenase activity	1.48	0.97	
I3SPJ0	Probably belonging to the mitochondrial respiratory chain complex	1.39	2.03	
I3S3T7	Probably belonging to the mitochondrial respiratory chain complex	1.21	0.84	
I3SVI7	Probably belonging to the mitochondrial respiratory chain complex	1.15	0.80	
G9JLU0	NADH dehydrogenase [ubiquinone] iron-sulfur protein 3, mitochondrial respiratory chain complex	0.82	1.33	
G9JLT6	ATP synthase subunit alpha, mitochondrial inner membrane	1.04	1.42	
I3S617	ATP synthase subunit d, mitochondrial	1.00	1.21	
I3S1V0	ATPase inhibitor	0.53	0.77	
I3SCF3	ATP synthase 24 kDa subunit, mitochondrial	1.52	0.87	

Table S8A: Amino acid metabolism, Microsymbiont

ID	Protein function, Symbiotic *Mesorhizobium loti*	FC	–log$_{10}$ q values	ΔG
	**Amino acid metabolism and Polyamines Biosynthesis**			
Q98AR2	Asparagine synthase (glutamine-hydrolyzing), generates asparagine from aspartate	1.07	0.77	
Q98AL8	Diaminobutyrate-2-oxoglutarate transaminase, conversion of L-aspartate beta-semialdehyde to L-2,4-diaminobutyrate, amine and polyamine biosynthesis; ectoine biosynthesis	-0.56	2.21	
Q98KB6	d-Ala-d-Ala ligase, peptidoglycan-biosynthesis	-1.17	1.32	
Q98AR6	L-aspartate:2-oxoglutarate aminotransferase	-0.47	2.28	
Q982X2	Agmatinase, putrescine biosynthetic process from arginine, using agmatinase	0.40	0.43	
**Q98H51**	Glutamate synthase large subunit, ammonia assimilation cycle, glutamate biosynthetic process	1.13	0.29	-6.76
Q98DQ4	Putative threonine dehydratase, isoleucin biosynthesis, L-serine ammonia-lyase activity, threonine deaminase activity	-0.31	2.08	
Q98NN0	Phosphoribosylformylglycinamidine synthase subunit PurQ, glutaminase activity, purine biosynthesis	-0.39	1.46	
Q98K89	Ornithine decarboxylase, putrescine biosynthetic process from ornithine	0.61	0.85	
Q98KK7	Acetolactate synthase small subunit, first common step of the biosynthetic pathway of the branched-amino acids	1.80	0.73	
Q98E51	3-isopropylmalate dehydratase small subunit, L-leucine biosynthesis	1.35	0.87	
Q98E57	3-isopropylmalate dehydrogenase, L-leucine biosynthesis	0.96	0.38	
Q98E71	Chorismate mutase, biosynthesis of phenylalanine and tyrosine	0.74	1.51	

Table S8B: Amino acid metabolism, Host

ID	Host Protein function, *Lotus japonicus* nodule	FC	–log_{10} q values	ΔG
	**Amino Acid Metabolism**			
I3T7L0	cyanoalanine nitrilase, release of ammonium ions from cyanoalanine	0.42	1.21	
I3T279	Aspartate aminotransferase	0.49	1.32	
D0VY45	2-isopropylmalate synthase, L-leucine biosynthesis	0.49	0.40	
I3SNI5	Chorismate mutase, aromatic amino acid family biosynthetic process	0.43	1.33	
Q43781	Aspartate aminotransferase	0.41	0.48	

Table S9A: Lipids, Microsymbiont

ID	Protein function, Symbiotic *Mesorhizobium loti*	FC	–log_{10} q values	ΔG
	**Lipids and Fatty Acid Metabolism**			
Q984T3	Acyl carrier protein AcpP, carrier of the growing fatty acid chain in fatty acid biosynthesis.	0.46	1.96	
Q98CP4	Palmitoyl-protein thioesterase, involved in cleavage of palmitate residues from acetylated proteins	-0.56	0.97	
Q98NC3	Biotin carboxylase, fatty acid biosynthetic process	1.33	0.50	
Q98FX7	Acetyl-coenzyme A carboxylase carboxyl transferase subunit alpha, fatty acid biosynthetic process	0.69	0.31	
Q98CY0	NodE, chain-elongation step of dissociated (type II) fatty-acid biosynthesis, nodEF leads to the synthesis of Nod factors containing a polyunsaturated C18 fatty acid side chain, fatty acid biosynthetic process [GO:0006633]	0.44	0.32	
Q984S4	LPS-assembly protein LptD, involved in the assembly of lipopolysaccharide (LPS) at the surface of the outer membrane	1.54	1.89	
Q98GS3	Lipopolysaccharide export system protein lpta 1	1.44	1.33	
Q98L53	Acyl carrier protein AcpXL	1.05	0.78	
Q988Y6	Acetoacetyl-CoA synthetase, lipid metabolic process	0.59	0.36	
Q98FS8	Protein with cytosolic phospholipase A2 catalytic domain, lipid degradation	0.56	1.52	
Q983N8	Acid phosphatase, phospholipid degradation	-0.80	2.18	
Q983L8	3-Deoxy-D-manno-octulosonic acid transferase, bacterial outer membrane biogenesis; LPS core biosynthesis	-1.19	1.15	
Q98ET6	Fatty acid synthase cyclopropane-fatty-acyl-phospholipid synthase	-1.28	1.28	
Q98E95	Acyl-CoA thioesterase, lysophospholipase activity	0.41	0.99	
Q98EG9	Acyl-CoA thioesterase II, fatty acyl-CoA hydrolase activity	-0.37	0.34	

Table S9B: Lipids, Host

ID 6	Host function, *Lotus japonicus* nodule	FC	–log_{10} q values	ΔG
	**Lipids and Fatty Acids**			
I3SY44	Non-specific lipid-transfer protein	2.01	0.82	
I3SA64	MD-2-related lipid-recognition domain-containing protein, intracellular sterol transport	1.08	1.38	
**I3SXY1**	Annexin, calcium/phospholipid-binding	0.99	0.56	-6.74
I3S9D4	Phospholipase D, phospholipid catabolic process	0.82	0.95	
I3T5Z3	3-Hydroxyacyl-[acyl-carrier-protein] dehydratase, unsaturated fatty acids biosynthesis	0.60	0.46	
I3S0B9	Plastid lipid-associated protein/fibrillin conserved domain-containing protein, accumulates in fibrillar-type plastids under stress conditions	0.41	1.14	

Table S10A: Hormone related processes, Microsymbiont

ID	Protein function, Symbiotic *Mesorhizobium loti*	FC	–log_{10} q values	ΔG
	**Hormone Related Processes**			
Q98FX1	Hippurate hydrolase, IAA-Ala conjugate hydrolase activity, release of auxin from inactive conjugates, auxin metabolic process, auxin contributes to early nodule development	0.92	0.58	
**Q989L9**	Oxidation of ent-kaurene into ent-kaurenoic acid, P450 monooxygenase involved in GA9 biosynthesis	-0.68	2.29	-6.80
Q989M2	Monooxygenase, converts GA12 into GA9	-0.88	2.29	

Table S10B: Hormone related processes, Host

ID 12	Host Protein function, *Lotus japonicus* nodule	FC	–log10 q values	ΔG
	**Hormones/Hormone induced, Stress**			
I3SM85	Delta (24)-sterol reductase, synthesis of campesterol, an early precursor of brassinolide.	-1.04	0.95	
I3SRT2	Bet v I/Major latex protein domain-containing protein abscisic acid-activated signaling pathway, pathogen defense response, PR-10 group (allergenic)	0.93	2.55	
I3SQK0	Bet v I/Major latex protein domain-containing protein, abscisic acid-activated signaling pathway, defense response, induction of PR genes (PR10), stress marker	2.30	1.55	
I3SGW8	Bet v I/Major latex protein domain-containing protein, abscisic acid-activated signaling pathway, pathogen defense response, PR-10 group (allergenic)	1.50	1.20	
I3T2N7	Bet v I/Major latex protein domain-containing protein, PR-10 group, abscisic acid-activated signaling pathway, pathogen defense response (allergenic)	0.91	1.58	
I3SL56	Bet v I/Major latex protein domain-containing protein, PR-10 group, abscisic acid-activated signaling pathway, pathogen defense response	1.44	2.29	
I3SPM5	Bet v I/Major latex protein domain-containing protein, abscisic acid-activated signaling pathway, alkaloid metabolic process, pathogen defense response, PR-10 group	0.62	0.76	
I3SSP7	Bet v I/Major latex protein domain-containing protein, abscisic acid-activated signaling pathway, pathogen defense response, PR-10 group	0.42	1.35	
Q3C1F4	Anaerobic nitrite reductase Glb1-1, arbuscular mycorrhizal association, response to abscisic acid, cytokinin, ethylene, response to symbiotic bacterium and fungi	0.21	1.35	
I3SF38	Dehydrin, response to abscisic acid, postulated to protect lipid membranes against peroxidation, water and cold stress	1.53	1.10	
I3S1F2	Water stress and hypersensitive response domain-containing	1.93	2.06	
I3S5I6	chitinase activity, defense marker	1.35	0.99	

Table S11A: Nitrogen, Microsymbiont

ID	Protein function, Symbiotic *Mesorhizobium loti*	FC	–log10 q values	ΔG
	**Nitrogen Fixation and Nitrogen Related Processes**			
Q98AP5	Nitrogenase molybdenum-iron protein beta chain	-1.07	2.36	
Q98AP7	Nitrogenase iron protein, key enzymatic reactions in nitrogen fixation iron component	-0.44	1.57	
Q98AU2	nitrogen fixation, NifT	0.13	0.50	
Q98AP3	Nitrogenase iron-molybdenum cofactor biosynthesis protein NifN (here mentioned because of the function)	-0.04	0.04	
Q98AT5	Nitrogen fixation protein FixB (here mentioned because of the function)	-0.14	0.14	
Q98AS8	Nitrogen fixation, iron-sulfur protein ferredoxin	-0.26	1.10	
Q98AT7	Nitrogen fixation, iron-sulfur protein ferredoxin	-0.37	1.33	
Q98AP2	Nitrogenase molybdenum-iron protein NifX	1.31	0.51	
Q98AT8	Nif-specific regulatory protein	-0.51	0.45	
Q98MX7	Nitrogen fixation; regulation of DNA-templated transcription	-0.67	0.80	
Q98AP6	Nitrogenase molybdenum-iron protein alpha chain	-0.74	2.23	
Q98EH0	Nitrogen regulatory protein P-II	1.86	2.14	

Table S11B: Nitrogen, Host

ID 5	Host Protein function, *Lotus japonicus* nodule	FC	–log10 q values	ΔG
	**Nitrogene fixation and Nitrogen metabolism**			
Q9FY16	Ferredoxin-nitrite reductase, chloroplastic, nitrate assimilation [GO:0042128] Nitrogen metabolism; nitrate reduction	1.11	2.32	
I3SL57	Heme oxygenase 1, plastid, required for efficient symbiotic nitrogen fixation (SNF) in root nodules, prevents H_2O_2 production under	0.54	1.22	

	stressful conditions, catalyses biliverdin production in senescing nodules			
Q3C1F3	Anaerobic nitrite reductase Glb1-2, Required for general plant development and during nodulation, especially for the onset of symbiosis	1.58	2.55	
I3SX86	Atypical leghemoglobin 2-1, reversibly binds oxygen O_2 facilitates the diffusion of oxygen to the bacteroids while preventing the bacterial nitrogenase from being inactivated by O_2, NO, CO	-0.52	1.58	
Q9SPM8	Nod factor binding lectin-nucleotide phosphohydrolase present on the surface of the root hairs, role in establishing rhizobium–legume symbiosis	1.15	1.67	

Table S12A: Signal transduction, Microsymbiont

**ID**	**Protein function, Symbiotic *Mesorhizobium loti***	**FC**	**$–log_{10}$ q values**	**ΔG**
	**Further Signal Transduction Related Processes**			
Q988T3	Histidine kinase, phosphorelay sensor kinase activity (complex variation of a two-component regulatory system), Signal transduction, regulation of DNA transcription	1.76	0.44	
Q98FM8	Sensory transduction regulatory protein, phosphorelay signal transduction system [GO:0000160], stress response regulator protein,	1.20	0.64	
Q987Y5	Two-component, response regulator, sequence-specific DNA binding, phosphorelay signal transduction system	-0.51	0.64	

Table S12B: Signal transduction, Host

**ID 9**	**Host Protein function, *Lotus japonicus* nodule**	**FC**	**$–log_{10}$ q values**	**ΔG**
	**Signal transduction, Special proteins**			
I3T321	14-3-3 domain-containing protein, interaction with phosphoserine on interacting protein, signal transduction	1.31	2.21	
I3S2P9	14-3-3 domain-containing protein, signal transduction	0.62	1.65	
I3SRT2	Bet v I/Major latex protein domain-containing protein abscisic acid-activated signaling pathway, pathogen defense response, PR-10 group (allergenic)	0.93	2.55	
I3SQK0	Bet v I/Major latex protein domain-containing protein, defense response, induction of PR genes (PR10), stress marker	2.30	1.55	
I3SGW8	Bet v I/Major latex protein domain-containing protein, abscisic acid-activated signaling pathway, pathogen defense response, PR-10 group (allergenic)	1.50	1.20	
I3T2N7	Bet v I/Major latex protein domain-containing protein, PR-10 group, abscisic acid-activated signaling pathway, pathogen defense response (allergenic)	0.91	1.58	
I3SL56	Bet v I/Major latex protein domain-containing protein, PR-10 group, abscisic acid-activated signaling pathway, pathogen defense response	1.44	2.29	
I3SPM5	Bet v I/Major latex protein domain-containing protein, abscisic acid-activated signaling pathway, alkaloid metabolic process, pathogen defense response, PR-10 group	0.62	0.76	
I3SSP7	Bet v I/Major latex protein domain-containing protein, abscisic acid-activated signaling pathway, pathogen defense response, PR-10 group	0.42	1.35	

Table S13A: Glutathione, Sulfur, Microsymbiont

**ID**	**Protein function, Symbiotic *Mesorhizobium loti***	**FC**	**$–log_{10}$ q values**	**ΔG**
	**Glutathione and Sulfur related processes**			
Q98DA7	N-methylhydantoinase A mikimopine-lactam hydrogenase 5-oxoprolinase (ATP-hydrolyzing) activity. γ-glutamyl cycle-like pathway to metabolize glutathione	1.21	1.04	
Q983T8	Probable glutathione S-transferase	2.13	1.02	
Q987U9	Glutamate-cysteine ligase, glutathione biosynthetic process	1.43	0.61	
Q98HM6	Glutathione transferase activity	1.00	0.57	
Q98NN1	Glutathione transferase	0.99	0.54	

Q984P7	Glutathione transferase activity, possesses also maleylacetoacetate isomerase activity, a key enzyme in the glutathione supported degradation of phenylalanine and tyrosine	0.58	1.06	
**Q98B64**	Glutathione hydrolase proenzyme, glutathione metabolic processes	0.30	1.23	-6.75
**Q98LU5**	Glutathione dependent detoxification of formaldehyde, S-(hydroxymethyl)glutathione dehydrogenase	0.65	0.22	-7.10
**Q98NF2**	Lactoylglutathione lyase, methylglyoxal degradation	-0.39	2.29	-6.54

Table S13B: Glutathione, Sulfur, Host

ID	Host protein function, *Lotus japonicus* nodule	FC	–$\log_{10}$ q values	ΔG
	**Glutathione related processes**			
I3SS38	Glutathione transferase, glutathione metabolic process	1.90	1.46	
I3SB85	Glutathione transferase, toxin catabolic process	1.56	1.64	
I3T0P3	Lactoylglutathione lyase, methylglyoxal degradation, glutathione release	1.40	0.78	
I3ST14	S-(hydroxymethyl)glutathione dehydrogenase, formaldehyde catabolic process	1.36	0.87	
I3SE57	Glutathione dehydrogenase (ascorbate), ascorbate glutathione cycle, plays a key role for H_2O_2 detoxification	1.17	0.69	
I3S9Z3	GST C-terminal domain-containing protein, glutathione transferase activity	1.05	0.82	
I3SCH3	Metallo-beta-lactamase domain-containing protein, sulfur dioxygenase activity, glutathione metabolic process	0.94	1.03	
I3SGQ7	S-formylglutathione hydrolase, hydrolyzes S-formylglutathione to glutathione and formate	0.74	0.42	
I3S4F4	GST N-terminal domain-containing protein, glutathione transferase activity	0.72	2.62	
I3T078	Glutathione-disulfide reductase, glutathione metabolic process	0.58	0.38	
I3SPG5	Glutathione S-transferase	0.52	1.72	
I3SRG4	Hydroxyacylglutathione hydrolase, methylglyoxal degradation, glutathione release	0.48	2.09	

Table S14A: ROS, Microsymbiont

ID	Protein function, Symbiotic *Mesorhizobium loti*	FC	–$\log_{10}$ q values	ΔG
	**ROS detoxification, superoxide dismutases, peroxidases, stress**			
Q985K3	Superoxid dismutase	0.66	2.55	
Q98EG4	Peroxidase activity, [response to oxidative stress GO:0006979]	-0.51	0.99	
Q98AY3	Cytochrome c peroxidase	-0.78	2.33	

Table S14B: ROS, Host

ID 18	Protein function, *Lotus japonicus* nodule	FC	–$\log_{10}$ q values	ΔG
	**ROS detoxification, superoxide dismutases, peroxidases, stress**			
Q56VR0	Superoxide dismutase, mitochondrion	0.52	1.45	
I3SA50	Superoxide dismutase, copper chaperone activity, cytosol	-0.60	0.86	
Q53D71	Superoxide dismutase, plastid	-1.65	2.57	
I3SIA9	Peroxidase, secreted	1.80	1.36	
I3SIJ1	Peroxidase, secreted	1.28	1.36	
I3T9D2	Peroxidase, secreted	1.26	1.11	
**I3T056**	Peroxidase, secreted	0.68	2.28	-6.72
I3T832	Plant heme peroxidase family, oxidation of toxic (phenolic) reductants, auxin catabolism, response to environmental stresses	0.41	2.28	
**I3T173**	L-ascorbate peroxidase, cellular response to oxidative stress, membrane	0.13	1.96	-6.73
**I3T6Q8**	Peroxidase, oxidation of toxic reductants, response to environmental stresses, extracellular region	0.07	0.47	-6.73
**Q56VS3**	(phospholipid-hydroperoxide) glutathione peroxidase, peroxidation-inhibiting protein activity, cytosol, mitochondrium, nucleus	0.37	1.36	

Q98HW0	Non-heme chloroperoxidase	0.02	1.36	
I3SQA4	thioredoxin-dependent peroxiredoxin, response to oxidative stress	0.88	0.04	
Q56VU1	Glutathione peroxidase, cytosol, response to oxidative stress	0.23	1.41	
Q987S0	Catalase-peroxidase, cellular response to hydrogen peroxide, cytosol	0.01	1.29	
I3T276	Catalase immune-responsive domain-containing protein	1.09	0.02	
I3T2A1	Ozone-responsive stress-related protein	0.53	2.29	
Q93VA1	Selenium binding protein, probably related to stress tolerance	0.41	2.32	

Table S15A: Proteolysis, Microsymbiont

ID	Protein function, Symbiotic *Mesorhizobium loti*	FC	–log10 q values	ΔG
	**Proteolytic processes**			
**Q982V5**	ATP-dependent Clp protease ATP-binding subunit ClpX, ATP-dependent specificity component of the Clp protease.	1.26	0.65	-6.67
Q984X4	Proline iminopeptidase, Release of N-terminal proline from a peptide.	1.25	0.87	
Q98KI9	Protein HflC, regulates a protease	0.95	1.10	
Q98H72	Oligoendopeptidase F, metalloendopeptidase	0.85	0.94	
Q983M2	Metallopeptidase	0.59	0.28	
Q98A65	Outer membrane serine protease	0.45	0.21	
Q98F02	Carboxyl-terminal protease, outer membrane-bounded periplasmic space	0.38	0.51	
Q98CT8	ATP-dependent protease subunit HslV, protease subunit of a proteasome-like degradation complex	0.31	1.33	
Q982V6	ATP-dependent Clp protease proteolytic subunit 2, Hydrolysis of proteins to small peptides in the presence of ATP and magnesium. Protein quality control for misfolded or incompletely synthesized proteins	-0.21	0.84	
Q986E5	Aminopeptidase, different Co-factors (Co^{2+}, Mg^{2+}, Zn^{2+}), exopeptidase	-0.41	2.34	
Q98GS1	Protease IV, serine-type peptidase, membrane	-0.59	2.09	
Q985F9	Probable periplasmic serine endoprotease DegP-like	1.20	1.32	
Q98J87	Probable serine protease, periplastic space	1.19	0.91	
Q98N31	Probable periplasmic serine endoprotease DegP-like, acts on substrates that are at least partially unfolded, cleavage pair of hydrophobic residues, such as Val-Val. efficient in the degradation of transiently denatured and unfolded proteins which accumulate in the periplasm following stress conditions.	1.16	0.45	
Q985A7	Signal peptidase I, serine-type endopeptidase activity, membrane	-0.35	1.11	
Q98ML7	PepSY domain-containing protein, probably a protease inhibitory function	1.19	2.50	

Table S15B: Proteolysis, Host

ID	Host protein function, *Lotus japonicus* nodule	FC	–log10 q values	ΔG
	**Proteolytic processes, Ubiquitin system**			
**A9QY38**	Subtilisin-like protease 4, required for arbuscular mycorrhiza (AM) development	0.38	1.67	-6.55
I3SPC4	Proteasome subunit beta	0.32	1.72	
I3SEA8	proteasome-mediated ubiquitin-dependent protein catabolic process	1.16	0.54	
**I3SZ47**	Cysteine protease, endopeptidase hydrolyses a polypeptide chain by a mechanism in which the sulfhydryl group of a cysteine residue at the active center acts as a nucleophile, extracellular space	0.28	1.60	-6.95
B0BLB2	ubiquitinyl hydrolase 1	-0.43	0.26	
I3SYA1	Serine carboxypeptidase-like 50	-0.54	0.38	
I3SLH5	cysteine-type endopeptidase inhibitor activity	-0.42	0.81	

Table S16: Flavonoids, Host

ID	Host protein function, *Lotus japonicus* nodule	FC	–log10 q values	ΔG
	**Flavonoid synthesis**			

**A0A411P2Y4**	Isoflavone reductase, a key enzyme in the biosynthesis of isoflavonoid phytoalexin, stress response, also lignan biosynthetic process	2.76	2.33	-6.67
Q8H0F6	Chalcone-flavanone isomerase 3, isomerization of chalcone into naringenin	1.42	2.33	
Q84KK4	Isoflavone 4'-O-methyltransferase	0.97	1.99	
I3STD3	Chalcone-flavonone isomerase family protein	0.50	2.01	
Q8H0G1	Chalcone-flavanone isomerase 2	0.38	0.17	
Q05JY0	Pterocarpan reductase, synthesis of pterocarpan-based isoflavonoid phytoalexins, response to biotic and abiotic stresses such as pathogen infection	1.55	1.21	
Q9MBE4	Cytochrome P450, Monooxygenase, oxidoreductase activity, involved in phytoalexin biosynthesis	2.43	1.29	

Table S17A: Miscellaneous, Microsymbiont

ID	Protein function Symbiotic *Mesorhizobium loti*	FC	$–\log_{10}$ q values	ΔG
	**Miscellaneous**			
Q98ER6	Secreted alkaline phosphatase, supplies a source of inorganic phosphate	1.87	1.31	
Q98HM3	Probable pyridoxine 5'-phosphate oxidase like enzyme, extracellular space, DUF domain protein	-1.2	2.65	
Q98C22	Probable oxidoreductase	1.16	0.46	
Q986E8	Probable ketosteroid isomerase, degradation of the steroid core.	0.95	2.29	

Table S17B: Miscellaneous, Host

ID 5	Host protein function, *Lotus japonicus* nodule	FC	$–\log_{10}$ q values	ΔG
	**Miscellaneous (selected)**			
I3SXK0	2-Oxoadipate dioxygenase/decarboxylase, uncharacterized function	0.48	2.34	
I3T816	Transmembrane protein, unknown function	1.0	1.62	
I3S8J3	Monodehydroascorbate reductase (NADH)	0.31	1.36	
**I3T443**	Profilin, binds to actin and affects the structure of the cytoskeleton. At high concentrations, profilin prevents the polymerization of actin, enhances it at low concentrations.	-0.85	2.57	-6.67
**I**3RZN5	Tubulin/FtsZ 2-layer sandwich domain-containing protein, has weak sequence homology to tubulin, GTPase, involved in polymer formation, microtubule cytoskeleton organization	1.62	0.84	

Tables Proteomes of Goitrin Treated free-living *Mesorhizobium loti*, Fraction 1 and 2

Tables S18-25 C. Fold change (FC) of down and up regulated proteins in fraction 1 (F1) and fraction 2 (F2).

Table S18 C. Transcription and pyrimidine/purine metabolism. F1: fraction 1, F2: fraction 2; FC: fold change; -log10 p values < 1.5 are significant. ID in bold: candidate for molecular docking; ΔG: estimated ΔG (kcal/mol).

ID	Protein function	F1/FC	-log p	F2/FC	-log p	ΔG
Q98N33	DNA-directed RNA polymerase alpha unit	0.01	0.01	-3.98	2.13	-6.49
Q98M41	Single-stranded DNA-binding protein	-0.95	0.30	-3.71	2.27	
Q98DY8	Transcription termination factor Rho	0.07	0.03	-3.34	1.80	
Q98MA8	Possesses endoribonuclease domain	nd		-3.31	2.70	
Q98I49	Transcription elongation factor GreA	-0.06	0.02	-2.55	1.67	
Q98BJ0	Transcription (anti)termination protein NusA	1.14	0.51	-2.73	1.52	
Q98N71	Transcription (anti)termination protein NusG	-1.49	0.36	-2.66	1.72	
Q987R3	GMP synthetase	-0.97	0.54	-2.41	1.00	
**Q98BI3**	Polyribonucleotide nucleotidyltransferase, mRNA degradation	0.24	0.33	-1.94	0.00	-6.50
Q98H62	Nucleotide catabolism	-1.42	1.69	-2.34	2.89	

**Q98F97**	Adenylosuccinate synthetase, purine nucleotide biosynthesis	0.22	0.47	-2.44	0.90	-6.62
Q984N8	Nucleoside triphosphate synthesis	-1.19	0.40	-2.70	1.16	
Q98NB6	Ribonuclease E, maturation of 5S and 16S rRNAs and the majority of tRNAs, degradation of most mRNAs	-2.87	1.80	nd		
**Q98NQ8**	Pseudouridine-5'-phosphate glycosidase	-1.52	0.94	nd		-6.58
Q988L2	Member of the two-component signal transduction system, alters gene expressions	1.44	0.36	nd		
Q98HM2	transcriptional regulator NolR	1.77	0.42	nd		
Q98CL8	Two-component system response regulator	1.13	0.41	nd		
Q98KC3	DNA repair protein RecN, probably involved in recombinational repair of damaged DNA, double-strand break repair	0.86	1.86	nd		
Q983K1	Transcriptional regulator	1.10	2.02	nd		
Q98I77	Probable cold shock protein, wide role in stress tolerance, regulation of gene expression	1.82	0.39	-5.26	1.55	
**Q98NQ6**	Product of repair gene recA, ATP-dependent DNA damage sensor activity, SOS response	-0.94	0.57	-1.19	3.29	-6.59

Table S19 C. Ribosomal proteins and proteins with functions in translation. F1: fraction 1, F2: fraction 2; FC: fold change; -log10 p values < 1.5 are significant. ID in bold: candidate for molecular docking; ΔG: estimated ΔG (kcal/mol). ID in bold: candidate for molecular docking; ΔG: estimated ΔG (kcal/mol).

**ID**	**Protein function**	**F1/ FC**	**-log p**	**F2/ FC**	**-log p**	**ΔG**
**Q98HV7**	bL25, binds to the 5S rRNA, stress protein CTC	nd		-6.15	2.28	-6.50
**Q98N55**	uL23, early assembly protein, binds 23S rRNA, docking site for trigger factor	-1.09	0.35	-4.50	3.03	-6.56
Q98N45	uL5, attachment of the 5S RNA into the large ribosomal subunit, contacts the P site tRNA	-0.88	0.29	-4.49	1.43	
Q984T8	bS6, binds together with bS18 to 16S rRNA	1.06	0.47	-2.82	0.00	
Q98N53	uS19, complex with S13 binds to 16S r RNA.	2.22	0.39	-4.21	3.66	
**Q98N46**	uL24, assembly initiator protein, binds to the 5'-end of the 23S rRNA	-0.73	0.14	-3.21	3.93	-6.5
Q98N49	uL29, interactions with L23 and with the 23S rRNA.	nd		-2.97	3.16	
Q98N54	uL2, primary rRNA binding protein. Required for association of the 30S and 50S subunits, for tRNA binding and peptide bond formation.	-1.88	0.41	-2.61	1.43	
Q98MB2	uS2, acts as an anchor for the polymerase	2.04	0.50	-3.82	1.35	
Q982W9	30S ribosomal protein S9, structural constituent of ribosome	2.16	0.75	-0.64	0.00	
Q98N38	uL15, binds the 5S rRNA, required for the late stages of subunit assembly, essential for 5S rRNA assembly onto the ribosome.	1.20	0.38	-2.67	1.14	
Q98N39	uL30, probably no essential ribosomal protein	0.60	0.10	-3.05	1.90	
Q98E70	bS16, unknown function	-1.69	0.35	nd		
P58168	bL19, binds directly to 23S rRNA, and is necessary for the assembly process of the 50S ribosomal subunit (in vitro).	1.40	0.70	3.74	1.36	
**Q983E9**	Aspartate-tRNA (Asp/Asn) ligase	-0.34	0.09	-2.53	1.80	-6.52
**Q98MV8**	Methionine-tRNA ligase	0.22	0.17	-2.11	1.75	-6.67
**Q98CQ1**	Phenylalanine-tRNA ligase beta subunit	nd		-1.73	3.74	-6.60
Q98FZ7	bL28, unknown function	-2.30	0.46	-2.73	2.34	
Q98CP8	bL20, binds to 23S ribosomal RNA	-3.07	0.69	nd		
Q98N69	uL1, binds to 23S rRNA, function in E site tRNA release	-0.80	1.57	-0.64	0.00	
Q98N61	uS12, role in translational accuracy	-1.86	0.66	nd		
**Q98N44**	30S ribosomal protein S14	-2.29	1.04	nd		-6.24
Q98N48	30S ribosomal protein S17, primary rRNA binding protein, binds specifically to the 5'-end of 16S ribosomal RNA	0.03	0.01	-3.51	1.97	

**Q98LB1**	Valine-tRNA ligase	-1.36	0.51	nd		-6.64
**Q983N4**	Isoleucine-tRNA ligase	-1.14	0.96	nd		-6.75
Q98N67	50S ribosomal protein L7/L12, interacts with GTP-bound translation factors	0.06	0.01	-0.27	0.00	
Q98HV5	Ribosome-binding ATPase YchF, binds to the large subunit	-2.31	0.90	nd		
Q98MB8	Ribosome-recycling factor	-1.30	0.57	-1.10	0.00	
Q98MB3	Elongation factor Ts	-0.61	0.39	-4.50	2.64	
Q98N59	Elongation factor G, catalyzes the GTP-dependent ribosomal translocation step during translation elongation	0.34	0.09	-2.28	0.00	
Q98F60	Elongation factor P 1, involved in peptide bond synthesis	0.03	0.01	-1.09	1.36	
Q98JB0	Spermidine/putrescine binding protein of ABC transporter, indicates reduced translation	0.62	0.37	-1.59	2.54	

Table S20 C. Chaperones. F1: fraction 1, F2: fraction 2; FC: fold change; -log10 p values < 1.5 are significant. ID in bold: candidate for molecular docking; ΔG: estimated ΔG (kcal/mol).
ID in bold: candidate for molecular docking; ΔG: estimated ΔG (kcal/mol).

**ID**	**Protein function**	**F1/FC**	**-log p**	**F2/FC**	**-log p**	**ΔG**
Q983S3	Co-chaperonin GroES 4, Protein folding	-1.63	0.3	-7.26	2.48	
**Q98AX9**	Chaperonin GroEL 3, Protein folding	-1.40	0.49	-3.47	1.84	-6.71
Q98GQ5	Protein GrpE, protein folding; preventing the aggregation of stress-denatured proteins	nd		-3.24	2.07	
Q98KJ1	Probable periplasmic serine endoprotease, chaperone activity	0.09	0.11	-2.49	1.74	
Q98DD1	Chaperone protein DnaK	-0.62	2.03	-0.90	0.00	
Q98G96	Chaperone protein ClpB	-1.40	1.25	nd		
Q98JB6	Chaperone protein HtpG	-1.56	0.84	nd		
Q98DU8	**Cytoplasmic protein-export protein SecB, protein folding, export of periplasmic and outer membrane proteins**	-1.86	0.43	nd		
Q98G68	Peptidyl-prolyl cis-trans isomerase, regulating protein folding at proline residues	-1.09	0.43	nd		
Q98LE8	peptidyl-prolyl cis-trans isomerase, regulating protein folding at proline residues	-1.38	0.50	-0.44	0.00	
Q98ME5	peptidyl-prolyl cis-trans isomerase, regulating protein folding at proline residues, PpiD chaperone family	1.86	0.57	-1.76	2.31	
Q988Y4	Phasin family protein, chaperone like function	-1.00	0.26	-2.91	2.10	

Table S21 C. Transporters, cell wall and outer membrane proteins (OMPs). F1: fraction 1, F2: fraction 2; FC: fold change; -log10 p values < 1.5 are significant. ID in bold: candidate for molecular docking; ΔG: estimated ΔG (kcal/mol).

**ID**	**Protein function**	**F1/FC**	**-log p**	**F2/FC**	**-log p**	**ΔG**
**Q98MK0**	ABC transporter, amino acid binding protein, bacterial solute-binding protein 3 family	-0.74	1.17	-6.31	4.60	-6.65
**Q98FL9**	ABC transporter, xylose binding protein of transport system XylF, bacterial solute-binding protein 2 family	-1.21	0.5	-5.20	3.75	-6.75
**Q98H18**	ABC transporter, periplasmic extracellular solute binding protein, thiamine binding/ transport, linked to iron transport	-0.06	0.04	-5.13	2.80	-7.35
**Q98BA6**	ABC transporter, Sugar binding protein bacterial solute-binding protein 2 family	-1.27	0.37	-4.92	2.77	-6.71
**Q98H33**	Periplastic protein. Part of sugar transport system, bacterial solute-binding protein 1 family	-0.53	0.17	-4.46	1.02	-6.93
Q98FL2	Part of the ABC transporter complex PstSACB involved in phosphate import, PstS family	-0.70	0.23	-4.44	2.66	
**Q98FA8**	ABC transporter, Amino acid binding protein, bacterial solute-binding protein 3 family	-2.02	0.81	-4.36	2.17	-6.70

**Q98GG9**	ABC transporter, Periplasmic ribose-binding protein, bacterial solute-binding protein 2 family	-0.34	0.46	-3.75	1.89	-6.65
**Q98MD4**	ABC transporter, (periplasmic) substrate-binding lipoprotein related	-1.52	0.53	-3.51	1.30	-6.85
**Q98CK3**	ABC transporter, sugar binding protein bacterial solute-binding protein 1 family	-1.72	0.69	-3.40	2.17	-6.64
**Q98EE6**	ABC transporter, Amino acid binding protein Solute-binding protein family 3 domain	-0.54	0.33	-3.00	2.48	-7.11
Q983A7	ABC transporter, Periplamic binding protein, betaine transport system, signal domain	nd		-2.26	1.39	
**Q98JL6**	ABC transporter, Ribose binding protein bacterial solute-binding protein 2 family	-0.13	0.05	-1.73	3.38	-6.54
**Q986W4**	ABC transporter, Amino acid binding protein leucine-binding protein family	nd		-1.04	2.99	-6.88
**Q985D9**	ABC transporter, probably amino acid binding protein, leucine-binding protein family	-1.44	0.29	-3.34	0.00	-6.56
**Q985N6**	ABC transporter, Peptide binding protein bacterial solute-binding protein 5 family	-0.92	0.91	-3.29	0.01	-6.61
Q985P8	ABC transporter, Sugar binding protein bacterial solute-binding protein 2 family	0.15	0.03	-3.20	1.29	
**Q98CD0**	ABC transporter, Amino acid binding protein, bacterial solute-binding protein 3 family	-2.01	0.90	-3.18	1.03	-7.00
**Q98BM2**	ABC transporter, Oligopeptide binding protein, Solute-binding protein family 5 domain	-1.05	0.53	-3.17	0.22	-6.62
**Q98LA8**	NodT candidate efflux transmembrane transporter activity, outer membrane factor (OMF) (TC 1.B.17) family	0.08	0.03	-2.65	1.41	-6.51
**Q982U6**	ABC transporter, Arabinose binding protein	-0.85	0.18	-3.15	0.37	-6.50
Q98NR5	ABC transporter ATP-binding protein, ABC transporter superfamily. Ycf16 family	0.01	0.01	-1.47	1.42	
Q98G45	Iron-binding periplasmic protein, mediates transport of iron, bacterial solute-binding protein 1 family	-2.21	1.41	-2.86	2.22	
Q98CF9	Periplasmic mannitol-binding protein, mediates transport	-0.95	0.41	-2.50	0.63	
Q98J19	ABC transporter, Ribose-binding protein bacterial solute-binding protein 2 family	-0.39	0.11	-2.43	1.46	
Q98JB0	Spermidine/putrescine binding protein of ABC transporter	0.62	0.37	-1.59	2.54	
Q98KC1	Outer membrane protein assembly factor BamD	-0.26	0.32	-1.66	1.65	
Q98NC0	Outer membrane protein, oxidoreductase activity, thioredoxin- like	-0.25	0.11	-1.01	2.23	
Q98MC3	Outer membrane protein assembly factor BamA	-1.32	1.18	-2.99	0.00	
Q984P5	Outer membrane protein, unknown function	-3.52	1.23	0.40	0.00	
Q98F85	Peptidoglycan-associated lipoprotein, part of the Tol-Pal system, maintaining outer membrane integrity.	-1.46	0.31	-3.91	1.15	
**Q98F84**	Tol-Pal system protein TolB, protein import, periplastic space	-0.23	0.30	-3.34	0.18	-6.64
Q98KI1	Molybdenum transport protein ModA, outer membrane-bounded periplasmic space, bacterial solute-binding protein ModA family	nd		-1.62	1.55	

Table S22 C. Primary Metabolic Processes. F1: fraction 1, F2: fraction 2; FC: fold change; -log10 p values < 1.5 are significant. ID in bold: candidate for molecular docking; ΔG: estimated ΔG (kcal/mol).

**ID**	**Protein function**	**F1/FC**	**-log p**	**F2/FC**	**-log p**	**ΔG**
**Amino Acid, Carbohydrate and Lipid Metabolism**						
Q983B6	Serine hydroxymethyltransferase 1	-1.10	0.33	-3.68	1.44	
Q98KM7	Ketol-acid reducto-isomerase, biosynthesis of branched-chain amino acids isoleucine, valine	0.59	0.18	-3.47	1.38	
**Q98A73**	Methionine biosynthesis	0.86	0.32	-3.65	0.87	-6.75
Q98N18	Nitrogen regulatory protein P-II, regulation of nitrogen utilization	-0.96	0.47	-1.70	1.96	
Q98I67	Aminotransferase	-1.14	0.62	-2.22	2.70	
Q98C77	UDP-glucose 6-dehydrogenase	-0.55	0.27	-3.23	1.47	
**Q98FJ1**	Phosphoglycerate kinase, glycolysis	-1.15	0.45	-2.54	2.43	-6.60

Q98CS6	Pyrophosphate-fructose 6-phosphate 1-phosphotransferase, first step of glycolysis	-1.28	1.63	-2.96	3.24	
Q98ME7	Triosephosphate isomerase, glycolysis	-1.19	0.84	-1.72	2.96	
Q98MY8	Oxireductase, pyruvate to acetyl-CoA, linking glycolysis and TCA cycle	0.06	0.01	-2.37	4.00	
Q98EK1	6-Phosphogluconate dehydrogenase, pentose phosphate pathway	-1.17	0.75	nd		
Q98C78	Endo-1,3-1,4-beta-glycanase ExoK	1.27	1.26	nd		
Q98N63	Galactose 1-dehydrogenase, galactose utilization	-2.65	0.90	nd		
	Glycerol kinase, glycerophospholipid biosynthesis	-0.83	0.94	nd		
Q98BH8	3-Hydroxydecanoyl-[acyl-carrier-protein] dehydratase, cis-unsaturation into fatty acids	-2.08	0.78	nd		
Q984T2	3-oxoacyl-[acyl-carrier-protein] synthase 2	1.67	1.37	nd		
**Tricarboxylic acid cycle (TCA), Respiratory Chain and Energy**						
**Q98EC8**	Succinate-CoA ligase subunit alpha, TCA	-3.27	0.73	-5.03	3.82	-6.68
Q98NQ2	Isocitrate dehydrogenase, TCA	-1.43	0.32	-4.18	1.98	
**Q98EC5**	Succinate-CoA ligase subunit beta TCA	-0.60	0.76	-3.53	1.75	-6.61
**Q98EV7**	ATP synthase gamma chain, ATP synthesis	nd		-3.26	2.64	-6.60
Q986D1	ATP synthase subunit b 2, ATP synthesis	nd		-1.81	2.64	
Q98EV8	ATP synthase subunit beta, ATP synthesis	-0.79	0.47	-1.63	0.09	
Q98MC9	Citrate synthase, moonlighting function: cell cycle regulation	3.99	3.17	nd		
Q98KQ7	NADH-quinone oxidoreductase subunit C	3.46	1.02	nd		
Q98KQ9	NADH-ubiquinone dehydrogenase chain E1, respiratory chain complex	-1.02	0.42	nd		
**Further Kinases and ATP binding Proteins**						
Q98HL8	Histidine kinase-like ATPase domain	-1.94	1.20	-0.96	2.86	
Q984N8	Nucleoside diphosphate kinase	-1.19	0.40	-2.07	1.16	
Q98M73	Glycerol kinase, glycerophospholipid biosynthesis	-0.83	0.94	nd		
Q98DC0	Probable N-acetylglucosamine kinase	-0.79	1.21	nd		
Q98C17	pfkB carbohydrate kinase family	nd		1.17	2.0	
Q98HW3	Ribose-phosphate pyrophosphokinase	0.66	0.34	nd		
Q98D76	Acetylglutamate kinase	1.27	0.32	nd		

Table S23 C. ROS detoxification and miscellaneous proteins. F1: fraction 1, F2: fraction 2; FC: fold change; -log10 p values < 1.5 are significant. ID in bold: candidate for molecular docking; ΔG: estimated ΔG (kcal/mol).

**ID**	**Protein function**	**F1/FC**	**-log p**	**F2/FC**	**-log p**	**ΔG**
Q987S0	Catalase/peroxidase	0.30	0.08	-1.36	0.95	
Q985K3	Superoxide dismutase	-0.53	0.17	-0.93	0.00	
**Q98M03**	Kinesin-like protein	-1.11	0.23	-2.62	1.38	-6.70
Q98F90	Related to antifreeze protein	-2.74	1.13	-3.68	2.52	
Q98EQ7	Putative LemA-like domain, unknown function	-0.81	0.49	-1.68	5.74	
Q98HD0	Flagellin, component of flagella	nd		-3.57	0.87	
Q98MA5	Probable Hit-like protein involved in cell-cycle regulation	1.16	1.72	nd		
**Q98FG0**	Non-mevalonate pathway of isoprenoid biosynthesis	-1.14	0.57	nd		-6.62
Q98D72	Phenylalanine-4-hydroxylase	1.70	0.40	nd		
Q98H83	Aspartate aminotransferase	-1.54	1.41	nd		

Table S24 C. Glutathione/Sulfur related Proteins. F1: fraction 1, F2: fraction 2; FC: fold change; -log10 p values < 1.5 are significant. ID in bold: candidate for molecular docking; ΔG: estimated ΔG (kcal/mol).

**ID**	**Protein function**	**Log2 FC**		**-log p**	
		**F1**	**F2**	**F1**	**F2**
Q98N11	Cysteine biosynthetic process from serine, O-acetylserine (thiol)lyase	-0.43	0.42	0.34	1.43
**Q98G02 ΔG -6.51**	Breakdown of methylglyoxal via S-lactoylglutathione, glutathione is regenerated in the 2. step, hydroxyacylglutathione hydrolase	-0.9	nd	0.97	
Q98I71	Probable glutathione S-transferase	0.21	nd	0.06	
Q98CX9	Glutathione S-transferase	-1.14	nd	0.53	

**Q98DE8 ΔG -6.70**	Glutathione synthetase	-0.31	nd	0.28	
**Q98DP1 ΔG -6.54**	L-cysteine biosynthesis, Cysteine synthase	-0.08	-1.47	0.18	1.43
Q98BJ4	S-adenosylmethionine biosynthesis, methionine adenosyl-transferase	0.11	-1.75	0.24	0.00
**Q98NF2 ΔG -6.54**	Lactoylglutathione lyase, release of glutathione and 2-oxopropanal from lactoylglutathione	1.22	nd	0.28	
Q985U5	Glutathione S-transferase kappa catalyzes the conjugation of glutathione to a wide range of hydrophobic substates for detoxification, thioredoxin-like	-0.52	nd	0.28	
**Q98HZ1 ΔG -6.59**	Protein-tyrosine sulfotransferase, protein, postranslational modification of protein-tyrosine, sulfatation	-0.50	nd	0.33	
Q98HV1	Probable thioesterase	-0.48	nd	0.40	
**Q98J86 ΔG -6.81**	Glutathione S-transferase	0.07	nd	0.09	
Q98K26	Sulfate binding protein of ABC transporter	0.12	nd	0.04	
Q987U9	Glutamate-cysteine ligase, first rate-limiting enzyme of glutathione synthesis	-0.22	nd	0.35	
Q98M60	Circularly permuted type 2 ATP-grasp protein, Glutathione synthetase ATP-binding domain-like	-0.75	nd	0.78	
**Q98LU5 ΔG -7.10**	Glutathione dependent detoxification of formaldehyde, S-(hydroxymethyl)glutathione dehydrogenase	-1.52	nd	0.32	
Q985V8	Glutathione-dependent peroxiredoxin thiol-specific peroxidase, GSH is used as the reductant	-0.09	-3.64	0.07	2.09
Q98CM9	Thioredoxin, protein-disulfide reductase activity, cell redox homeostasis, serves as a general protein disulphide oxidoreductase	-1.83	-3.40	0.39	1.51
Q98E31	Thioredoxin, protein-disulfide reductase activity, cell redox homeostasis, serves as a general protein disulphide oxidoreductase	-0.17	-2.54	0.10	1.54

Table S25 C. Proteases, Peptidases and Protease Inhibitors. F1: fraction 1, F2: fraction 2; FC: fold change; -log10 p values < 1.5 are significant. ID in bold: candidate for molecular docking; ΔG: estimated ΔG (kcal/mol).

ID	Protein function		Sig		Sig
		F1FC	F1	F2FC	F2
**Q982V5 ΔG -6.67**	Component of the Clp protease complex, heat shock protein involved in DNA repair, ATP-dependent Clp protease ATP-binding subunit ClpX, facilitates unfolding	2.62	0.58	nd	
Q98K54	N-carbamoyl-beta-alanine amidohydrolase, exopeptidase	-0.19	0.05	nd	
Q98EC1	Outer membrane lipoprotein omp19 homolog, N-palmitoyl cysteine lipidation, alkaline proteinase inhibitor	0.25	0.13	nd	
**Q98CU1**	ATP-dependent protease ATPase subunit HslU, ATPase subunit of a proteasome-like degradation complex; has chaperone activity.	0.72	0.23	-0.60	0.10
Q98EQ1	Peptidyl-dipeptidase	-0.29	0.10	nd	
Q984S1	Probable cytosol aminopeptidase	0.01	0.01	nd	
Q983M2	metallopeptidase activity	0.12	0.12	nd	
Q98KI8	Protein HflK, encode or regulate a protease	-0.53	0.21	nd	
Q98F53	Metal-dependent carboxypeptidase	-0.70	0.26	nd	
Q98MF5	Proline iminopeptidase	-0.26	0.26	nd	
Q985A7	Signal peptidase I, cleavage of N-terminal signal or leader sequences from secreted and periplasmic proteins.	-0.60	0.54	nd	
Q98HW5	Probable hydrolase/peptidase	-0.72	0.24	nd	
Q98KC8	Aminopeptidase P	-0.98	0.60	nd	
Q98F88	ATP-dependent zinc metalloprotease FtsH, quality control of integral membrane proteins	0.30	0.11	0.4	1.58
Q98CT8	ATP-dependent protease subunit HslV	-0.37	0.22	nd	
Q98FP3	Membrane dipeptidase	1.15	0.77	nd	
Q98BF0	Leucine aminopeptidase	-0.58	1.53	nd	

Q98F02	Carboxyl-terminal protease	-0.52	0.53	nd
Q98CJ2	Dipeptidyl-peptidase activity	0.78	0.81	nd
Q98KI9	Protein HflC, peptidase activity,	-0.79	0.36	nd
Q986C4	Serine-type peptidase activity	0.33	0.54	nd
Q98NB9	Metalloendopeptidase activity	-0.04	0.02	nd

Table S26: -ΔG values of Proteins (ID) Goitrin Docking Analysis.

	**UniProtKB ID**	**ΔG [kJ/mol]**	**UniProtKB ID**	**ΔG [kJ/mol]**	**UniProtKB ID**	**ΔG [kJ/mol]**
1	Q98H18	-7.296	Q982U6	-6.508	Q98BY4	-6.313
2	Q98EE6	-7.108	Q98HV7	-6.501	Q98BJ0	-6.311
3	Q98LU5	-7.101	Q98N46	-6.499	Q98HR5	-6.311
4	Q98CD0	-6.989	Q98FL9	-6.491	Q98FL2	-6.31
5	Q986W4	-6.879	Q989M2	-6.489	Q98MJ3	-6.306
6	Q98MD4	-6.846	Q98LA8	-6.487	Q983S3	-6.3
7	Q98J86	-6.818	Q98CX9	-6.482	Q98M41	-6.298
8	Q98MF6	-6.813	Q98MA8	-6.48	Q98HL8	-6.295
9	Q98MB1	-6.803	Q98HV1	-6.47	Q98BJ4	-6.294
10	Q98CQ1	-6.781	Q98BI3	-6.468	Q98NC0	-6.292
11	Q989L9	-6.773	Q985V8	-6.468	Q984P5	-6.289
12	Q98H51	-6.766	Q988L2	-6.464	Q98HM2	-6.287
13	Q98B64	-6.756	Q98I71	-6.458	Q98M60	-6.284
14	Q98A73	-6.752	Q98IH9	-6.456	I3T2A1	-6.284
15	Q983N4	-6.749	Q98NQ2	-6.449	Q98N18	-6.283
16	I3SXY1	-6.743	I3SM85	-6.449	P58168	-6.275
17	Q98AX9	-6.71	Q98MB2	-6.44	Q98N71	-6.271
18	Q98MO3	-6.699	Q98DY8	-6.44	I3SJ57	-6.27
19	Q98FA8	-6.696	I3RZN5	-6.44	Q98N68	-6.263
20	Q98DE8	-6.694	Q98B45	-6.431	Q98N44	-6.242
21	Q98EC8	-6.683	Q98M73	-6.422	Q98MC9	-6.235
22	Q98MV8	-6.674	Q98EQ7	-6.421	Q98HX8	-6.234
23	Q982V5	-6.672	Q98N55	-6.417	Q98FP3	-6.233
25	A0A411P2Y4	-6.671	Q98HV5	-6.407	Q98ME5	-6.225
26	Q98G69	-6.656	Q98ER6	-6.407	Q987U9	-6.207
27	Q98MK0	-6.65	Q984N8	-6.403	Q983A7	-6.203
28	Q98LB1	-6.643	Q98N11	-6.402	Q988L3	-6.178
29	Q98CK3	-6.641	Q98N54	-6.401	Q98E31	-6.166

30	Q98F97	-6.628	Q98BF0	-6.4	Q98K26	-6.142
31	Q98BM2	-6.625	Q98N53	-6.393	Q985K3	-6.129
32	Q98FG0	-6.624	Q98N33	-6.389	Q98I3S	-6.114
33	Q985N6	-6.618	Q98DU8	-6.388	I3SD49	-6.114
34	I3T443	-6.618	Q98C77	-6.385	Q98KC1	-6.112
35	Q98EC5	-6.615	Q98H33	-6.377	Q98GS3	-6.111
36	Q98F84	-6.611	Q98EB1	-6.377	Q98GQ5	-6.093
37	Q98FJ1	-6.60	Q983B6	-6.375	Q98CM9	-6.066
38	Q98EV7	-6.598	Q98N45	-6.365	Q98NM7	-6.058
39	Q98HZ1	-6.59	Q98EV8	-6.362	I3SPM6	-6.051
40	Q98NQ6	-6.589	Q98G45	-6.36	I3RZH4	-6.025
41	Q98NQ8	-6.582	Q98ME7	-6.36	Q985U5	-6.018
42	I3S413	-6.569	Q98CS6	-6.36	Q98IL8	-5.989
43	Q98GF4	-6.566	Q985P8	-6.358	Q98I49	-5.96
44	Q985D9	-6.563	Q98F90	-6.357	Q98N67	-5.959
45	A9QY38	-6.548	Q98BA6	-6.344	Q984T8	-5.954
46	Q98NF2	-6.545	Q98KM7	-6.33	Q98N49	-5.951
47	Q98JL6	-6.545	Q98G68	-6.327		
48	Q98DP1	-6.541	Q987B0	-6.323		
49	Q987R3	-6.525	Q98FL2	-6.32		
50	Q983E9	-6.519	Q98II0	-6.317		
51	Q98G02	-6.514	Q98N57	-6.316		

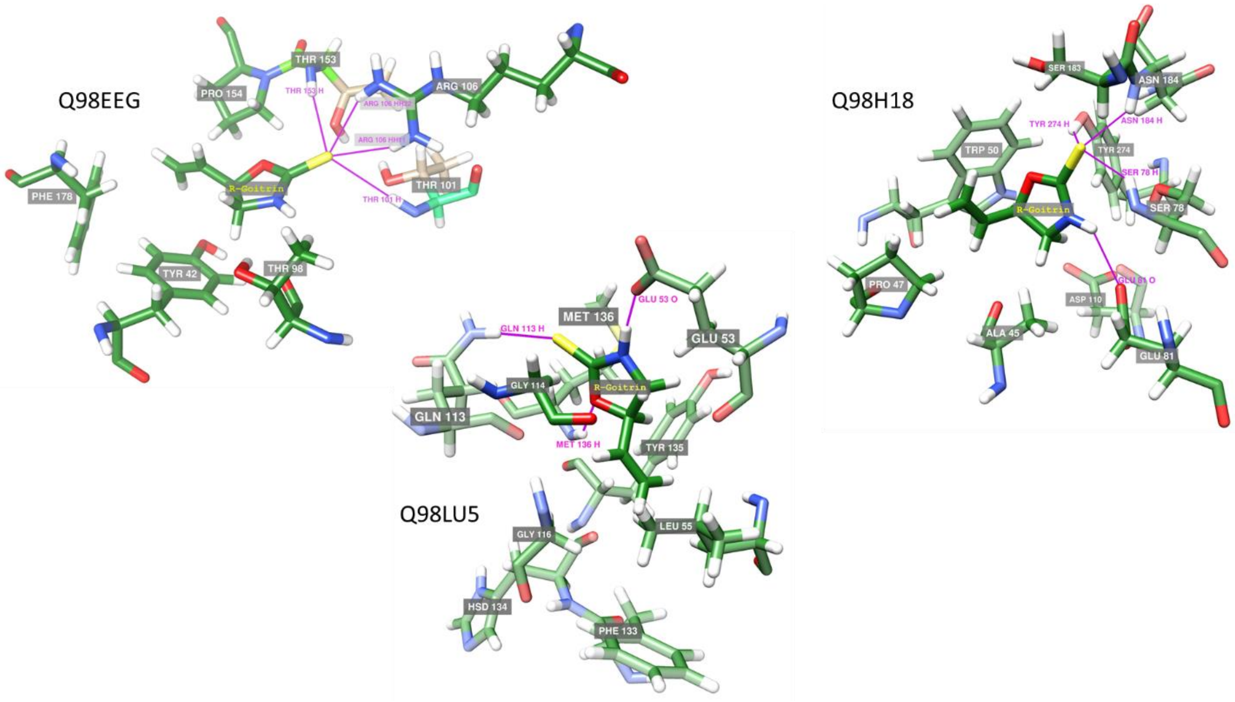

Figure S1 Interaction site of R-goitrin (ligand) and target proteins Q98EE6, Q98H18, and Q98LU5. Goitrin is represented as sticks. Only AAs within a maximum distance of 3Å are shown and labelled with three letter codes. Potential H-bonds between the ligand and respective AA residues are indicated by purple lines. Carbon atoms are colored in green; oxygen and

nitrogen atoms are depicted in red and blue, respectively, and sulfur atoms are shown in yellow. The presented models highlight the thione group as a special part of the goitrin molecule that strongly contributes to complex formation. The 2-thione group complexes with one bond to Q98LU8), with three to Q98H18 and with four bonds to Q98EE6.

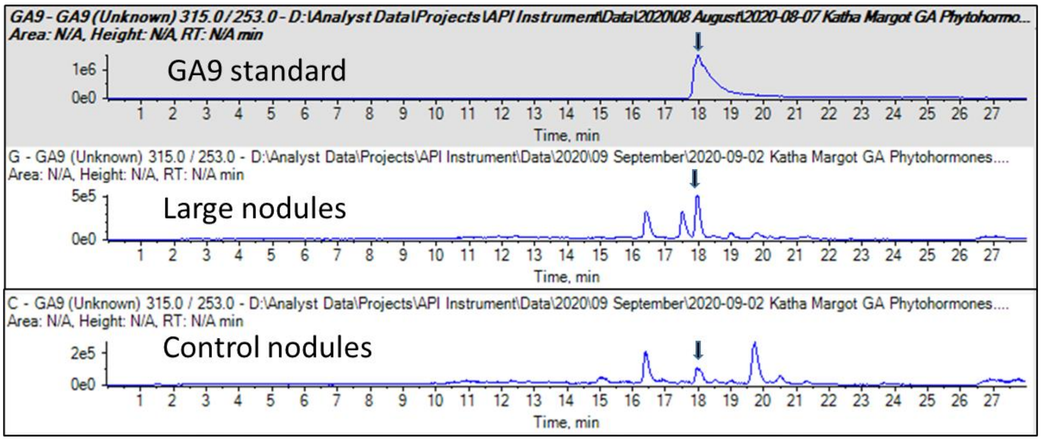

Figure S2 GA9 contents in the large nodules (**50mg**) and nodules of the control plants (**50mg**). The content in the goitrin treated nodules were 3.5fold higher than in the control.

Table S27: MRM parameters for phytohormones.

Phytohormones				
**Analyte**	RT [min]	Q1	Q3	Q2 (V)
**GA4**	18.0	331.1	212.7	-24
**GA9**	16.7	315.0	253.0	-24
